# Orogeny and topography influenced Jurassic–Cretaceous terrestrial ecosystem evolution in northeastern Asia

**DOI:** 10.1093/nsr/nwag100

**Published:** 2026-02-13

**Authors:** Nan Wang, Zhiyong Zhang, Peter Luffi, Zhiheng Li, Robert A Spicer, Zhiqiang Yu, Bo Wan, Jing-Jing Zhu, Jien Zhang, Songjian Ao, Dongfang Song, Dunfeng Xiang, Chao Guo, Wenjiao Xiao

**Affiliations:** State Key Laboratory of Lithospheric and Environmental Coevolution, Institute of Geology and Geophysics, Chinese Academy of Sciences, Beijing 100029, China; State Key Laboratory of Critical Mineral Research and Exploration, Institute of Geochemistry, Chinese Academy of Sciences, Guiyang 550081, China; State Key Laboratory of Lithospheric and Environmental Coevolution, Institute of Geology and Geophysics, Chinese Academy of Sciences, Beijing 100029, China; Faculty of Geology and Geophysics, University of Bucharest, Bucharest 010041, Romania; Sabba Stefanescu Institute of Geodynamics, Bucharest 020032, Romania; Geological Institute of Romania, Bucharest 012271, Romania; Key Laboratory of Vertebrate Evolution and Human Origins, Institute of Vertebrate Paleontology and Paleoanthropology, Chinese Academy of Sciences, Beijing 100044, China; Laboratory of Tropical Forest Ecology, Xishuangbanna Tropical Botanical Garden, Chinese Academy of Sciences, Mengla 666303, China; School of Environment, Earth and Ecosystem Sciences, The Open University, Milton Keynes MK7 6AA, UK; State Key Laboratory of Lithospheric and Environmental Coevolution, Institute of Geology and Geophysics, Chinese Academy of Sciences, Beijing 100029, China; State Key Laboratory of Lithospheric and Environmental Coevolution, Institute of Geology and Geophysics, Chinese Academy of Sciences, Beijing 100029, China; State Key Laboratory of Critical Mineral Research and Exploration, Institute of Geochemistry, Chinese Academy of Sciences, Guiyang 550081, China; State Key Laboratory of Lithospheric and Environmental Coevolution, Institute of Geology and Geophysics, Chinese Academy of Sciences, Beijing 100029, China; State Key Laboratory of Lithospheric and Environmental Coevolution, Institute of Geology and Geophysics, Chinese Academy of Sciences, Beijing 100029, China; State Key Laboratory of Lithospheric and Environmental Coevolution, Institute of Geology and Geophysics, Chinese Academy of Sciences, Beijing 100029, China; State Key Laboratory of Lithospheric and Environmental Coevolution, Institute of Geology and Geophysics, Chinese Academy of Sciences, Beijing 100029, China; Xinjiang Research Center for Mineral Resources, Xinjiang Institute of Ecology and Geography, Chinese Academy of Sciences, Urumqi 830011, China; State Key Laboratory of Lithospheric and Environmental Coevolution, Institute of Geology and Geophysics, Chinese Academy of Sciences, Beijing 100029, China; Department of Geology, University of Vienna, Vienna 1090, Austria; Xinjiang Research Center for Mineral Resources, Xinjiang Institute of Ecology and Geography, Chinese Academy of Sciences, Urumqi 830011, China

**Keywords:** Northeastern Asia, paleo-elevation estimation, paleo-temperature reconstruction, high-relief landscapes, ecosystem evolution

## Abstract

Tectonic processes are often invoked to explain ecosystem changes, but their precise effects remain elusive. This study focuses on Jurassic–Cretaceous Northeastern Asia, linking the flourishing of the globally exceptional Yanliao and Jehol Biotas, which are temporally successive biotas with distinct species composition, to a prominent tectonic transition from crustal shortening to extension. We estimated paleo-elevation and paleo-temperature variations using whole-rock chemical parameters from Jurassic–Early Cretaceous continental arcs. Combined with published paleoclimate and paleontological records, our findings suggest that Mid–Late Jurassic plate convergence in Northeastern Asia created high elevations and complex topography with vertically zoned micro-environments, promoting the emergence of Yanliao Biota. In the Early Cretaceous, following recovery from a warm and arid climate interval across the Jurassic–Cretaceous transition, enhanced topographic ruggedness due to tectonic extension and local topographic/climate heterogeneity, diversified the Jehol Biota. This highly sculpted Northeastern Asia upland (2.0–4.5 km) hosted a wide spectrum of ecological niches under a relatively cool but heterogeneous climate, creating a key cradle for biodiversification.

## INTRODUCTION

At active continental margins, a growing body of work proposes that plate tectonic and landscape dynamics play a significant role in the evolution of the climate, environment, and biosphere [1,2]. Intense tectonic activity results in regional mountain uplift, exhumation, and erosion, and generates complex topographical features [1,3]. The resultant topographic heterogeneity, in turn, profoundly influences regional temperature, precipitation, soil types, and biodiversity [2–5].

Along the Northeastern (NE) Asian continental margin, the Mid–Late Jurassic Yanliao and Early Cretaceous Jehol Biotas represent two of the most celebrated terrestrial lagerstätten worldwide [6–9]. The Yanliao Biota contains diverse vertebrates and abundant insect and plant species, providing critical fossil records for the origin and radiation of early mammals and for the evolutionary transition from dinosaurs to birds [8]. The Jehol Biota, often referred to as the ‘Mesozoic Pompeii,’ includes exceptionally well preserved terrestrial vertebrates, invertebrates, and plants, and records the origins and diversification of major Cretaceous lineages, including birds, eutherian mammals, and flowering plants [6,7,10].

Recent studies highlight that the rise and fall of these biotas are intricately linked to regional climate fluctuations [11–13]. For instance, the Mid–Late Jurassic climate transition coincides with the

explosion of the Yanliao Biota [12], while the transition from the Yanliao to Jehol biota occurred during environmental upheaval spanning the latest Jurassic to earliest Cretaceous [13–15]. However, these remarkable biological transformations unfolded against a backdrop of muted long-term global climate dynamics, with a relatively warm climate prevailing throughout the period [16]. Significantly though, these biological upheavals coincided with a prominent Jurassic–Cretaceous tectonic transition in NE Asia [17,18]. This shift involved a change from crustal shortening and compressional deformation [19,20] to craton extension and destruction [21–23], and is reflected in the evolution of crustal thickness and elevation. Such a geological context prompts an investigation into how tectonic processes influenced topographic and climate change and the evolution of ecosystems at the NE Asian plate margin.

**Figure 1.**
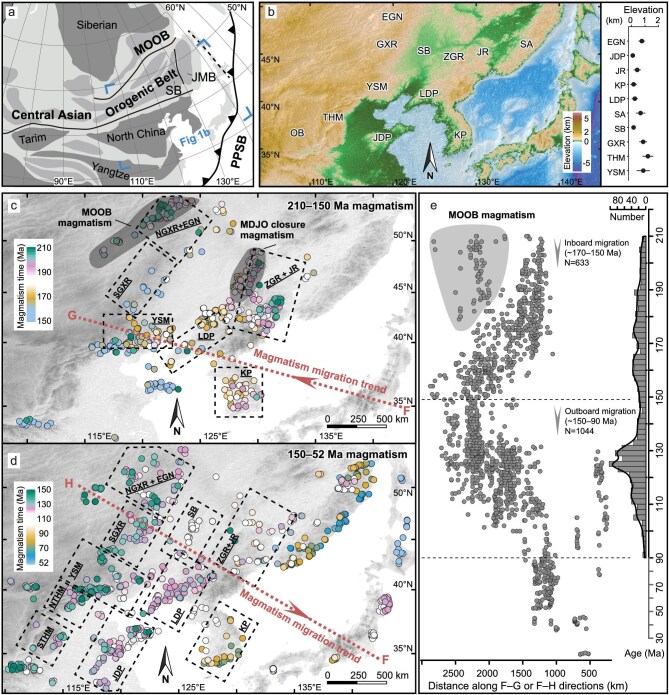
Tectonic subdivision, topography, and magmatic evolution of NE Asia. (a) Schematic tectonic subdivision of NE Asia, modified after Liu *et al.* [47]. PPSB–Paleo-Pacific Subduction Belt; MOOB–Mongol-Okhotsk Collision Belt; SB–Songliao Block; JMB–Jiamusi Block. (b) Present-day topography map of NE Asia, with an inset diagram displaying present-day mean elevation of arc segments in NE Asia. (c, d) Inboard and outboard migration of magmatism in NE Asia (110°E to 145°E) between 210 Ma and 0 Ma. (e) Spatiotemporal distribution of igneous rocks in NE Asia projected along the F-G and F-H sections in panels (c) and (d), with the kernel density estimate (KDE) of zircon U-Pb age distribution between 210 Ma and 90 Ma. Abbreviations: EGN–Erguna Region; GXR–Great Xing’an Range; SGXR–Southern Great Xing’an Range; NGXR–Northern Great Xing’an Range; ZGR–Zhangguangcai Range; JR–Jiamusi Region; SA–Sikhote-Alin; YSM–Yanshan Mountains; LDP–Liaodong Peninsula; JDP–Jiaodong Peninsula; THM–Taihangshan; STHM–Southern Taihangshan; NTHM–Northern Taihangshan; OB–Ordos Basin. The bedrock zircon U-Pb data can be found in Table S1.

Estimates of crustal and lithospheric thicknesses derived from geobarometry on lower crustal xenoliths and mafic magmas, indicate significant crustal and lithospheric thinning of the North China Craton during the Late Mesozoic [24–26]. Several paleo-elevation reconstruction studies of major mountains and basins in NE Asia relying on whole-rock geochemistry, zircon Eu anomaly, carbonate clumped-isotope, and basalt-vesicularity, have revealed elevations of 2.8–4.1 km for the late Early Cretaceous Yanshan Mountains [11], 2.7 ± 0.7 km for the late Early Cretaceous Taihangshan [27], at least 2 km for the early Late Cretaceous Jiaolai Basin [28], 4.7 ± 0.8 km for the Early Cretaceous Inner Mongolia region [29], and ∼4.0–5.0 km for the Early Cretaceous northeastern North China Craton [30], respectively. All above studies suggest a significant contrast between the estimated paleo-elevations and present-day average elevations in NE Asia (<1.2 km; Fig. 1b), implying substantial topographic change in the region since late Mesozoic times.

While the results mentioned above offer valuable constraints on the elevations for specific times and places, they are insufficient to adequately characterize the Jurassic–Cretaceous topographic and climate variability and evolution of NE Asia at a regional scale. To obtain a more systematic and detailed insight into the topographic evolution of the Jurassic–Cretaceous NE Asia, we examined the composition of magmatic rocks sampled across the region.

Whole-rock chemical parameters of igneous rocks formed along convergent plate margins, such as La/Yb and Sr/Y, have long been recognized as proxies for crustal thickness (aka chemical mohometers) in orogens developed within ancient subduction and collision zones [31–35]. Given the isostatic equilibrium observed in magmatic arcs [33,36], these mohometers can successfully predict the paleo-elevations of orogens as well. The novel multi-proxy strategy proposed by Luffi and Ducea [33] improves the precision and robustness of the approach and enables direct quantification of paleo-elevation. Based on regional mean elevation through terrestrial thermal lapse rates [37,38], and latitudinal gradients in solar radiation [16], regional mean annual temperature (MAT) from the geological past can be estimated.

In this contribution, in a first step we employ the multi-proxy strategy of Luffi and Ducea incorporated in the Geochemical Arc Moho Estimator (GAME) app [33] to quantify how elevation variations tracked the continuous inboard and outboard migration of arc magmatism across NE Asia in response to changing dynamics of the subducted Paleo-Pacific slab throughout the Jurassic and Early Cretaceous [21,39,40]. We then reconstruct paleo-temperatures for the Mid–Late Jurassic–Early Cretaceous NE Asia using the estimated paleo-elevations, published paleo-latitude data [15], and Phanerozoic global temperature models [16,41]. Finally, we discuss the impact of these topographic changes on the regional evolution of terrestrial ecosystems, drawing on published paleoclimate and paleontological datasets within the conceptual framework of mountain biodiversity [4,5].

## RESULTS

### Paleo-elevation reconstruction

In order to reconstruct the topographic evolution of the NE Asian orogenic belt, we mainly utilized the chemical compositions of samples representing 100–200 Ma old igneous rocks formed during the inboard migration and retreat of the arc magmatism associated with the Paleo-Pacific subduction (Fig. 1c–e) [21,40]. By grouping these samples based on their geographic distribution, age, and geological context, we defined 26 spatially and temporally delimited chemical datasets representative of the evolution of 10 arc segments. Using these datasets, we then calculated 26 paleo-elevations and crustal thicknesses with the help of GAME. The obtained results are summarized in Table 1 and discussed below; technical details are presented in Table S3 and Fig. S5.

**Table 1.** Paleo-elevation and paleo-crustal thickness estimates for the Jurassic–Early Cretaceous magmatic arc segments in NE Asia.

Arc segments	Time interval (Ma)	Crustal thickness (km) (median)	Error (MAD)	Elevation (km) (median)	Error (MAD)
JDP	133–125	56.8	4.4	3.9	0.6
JDP	125–110	57.3	5.6	4.0	0.7
SB	115–102	44.1	5.0	2.3	0.6
KP	192–179	50.3	5.0	3.1	0.6
KP	179–165	55.9	2.2	3.8	0.3
KP	112–109	58.9	3.1	4.2	0.4
LDP	135–125	56.7	3.7	3.9	0.5
LDP	124–116	56.6	4.3	3.9	0.5
ZGR + JR	210–196	35.0	6.5	1.1	0.8
ZGR + JR	196–184	39.4	6.7	1.7	0.9
ZGR + JR	183–173	50.2	5.3	3.1	0.7
ZGR + JR	113–100	52.8	6.0	3.4	0.8
NGXR + EGN	206–186	43.6	2.5	2.2	0.3
NGXR + EGN	180–160	60.7	2.2	4.4	0.3
NGXR + EGN	143–128	54.3	5.3	3.6	0.7
NGXR + EGN	128–112	55.4	3.9	3.7	0.5
SGXR	160–140	50.1	5.2	3.0	0.7
SGXR	140–130	47.6	3.1	2.7	0.4
SGXR	130–120	47.7	3.2	2.7	0.4
YSM	173–160	60.4	4.4	4.4	0.6
YSM	160–150	57.1	4.0	3.9	0.5
YSM	140–125	59.8	4.1	4.3	0.5
YSM	125–122	60.7	3.1	4.4	0.4
STHM	135–125	50.9	4.5	3.1	0.6
NTHM	146–138	57.4	4.8	4.0	0.6
NTHM	133–120	60.2	4.3	4.3	0.6

Abbreviations: STHM–Southern Taihangshan; NTHM–Northern Taihangshan; YSM–Yanshan Mountains; SB–Songliao Basin; JDP–Jiaodong Peninsula; ZGR–Zhangguangcai Range; SGXR–Southern Great Xing’an Range; NGXR–Northern Great Xing’an Range; KP–Korea Peninsula; EGN–Erguna Region; JR–Jiamusi Region. Note that Moho depth is typically referenced to mean sea level, and crustal thickness is the sum of Moho depth and elevation a.s.l.

The Late Triassic–Early Jurassic Zhangguangcai Range-Jiamusi Region exhibits paleo-elevations increasing over time from 1.1 ± 0.8 km to 3.1 ± 0.7 km. Similarly, during the Early–Middle Jurassic, both the Korea Peninsula and the Northern Great Xing’an Range-Erguna Region exhibit increasing paleo-elevations from 3.1 ± 0.6 km to 3.8 ± 0.3 km, and from 2.2 ± 0.3 km to 4.4 ± 0.3 km, respectively. The Yanshan Mountains achieved high paleo-elevations of between 3.9 ± 0.5 km and 4.4 ± 0.6 km in the Mid–Late Jurassic. During the Early Cretaceous, the Southern Taihangshan, the Southern Great Xing’an Range, the Songliao Basin, and the Jiamusi Region-Zhangguangcai Range were characterized by relatively low paleo-elevations of between 2.3 km and 3.4 km. In contrast, the Liaodong-Jiaodong-Korea Peninsula, the Yanshan Mountains, the Northern Taihangshan, and the Northern Great Xing’an Range-Erguna Region maintained relatively high paleo-elevations of between 3.6 km and 4.4 km.

### Paleo-temperature reconstruction

The two-step paleo-temperature reconstruction approach adopted here primarily relies on paleo-latitude, sea-level temperature, and paleo-elevation for each Jurassic–Early Cretaceous arc segment. Sea-level temperatures at different latitudes for this time interval were derived from Phanerozoic pole-to-equator temperature gradient models [16,41]. Given the abundance of published paleo-latitude constraints for the Jurassic–Early Cretaceous Yanshan Mountains [15,42], this region was used as a reference point, and present-day latitudinal differences between the Yanshan Mountains and other arc segments were applied to estimate paleo-latitudes for each segment.

The paleo-temperature reconstruction results are presented in Tables 2 and S4. During the Mid–Late Jurassic–Early Cretaceous, the mean annual paleo-temperatures (paleo-MAT) of Yanshan Mountains, South Great Xing’an Range, and Korea Peninsula were between 0.4 ± 5.6°C and 6.4 ± 5.1°C, between 6.0 ± 4.0°C and 9.5 ± 5.1°C, and between 3.4 ± 4.9°C and 12.8 ± 5.5°C, respectively. In the north, estimates for the Northern Great Xing’an Range-Erguna Region and Zhangguangcai Range-Jiamusi Region yield paleo-MATs from −1.0 ± 5.5°C to 3.8 ± 5.2°C, and from 3.9 ± 6.1°C to 11.1 ± 5.6°C, respectively. During the Early Cretaceous, Northern and Southern Taihangshan, and Jiaodong and Liaodong Peninsulas exhibit paleo-MATs from 0.7 ± 5.7°C to 8.0 ± 4.9°C, and from 3.3 ± 4.9°C to 7.2 ± 6.4°C, respectively. However, the low-elevation Songliao Basin was characterized by warmer paleo-MATs of 8.8 ± 4.8°C.

**Table 2.** Paleo-temperature estimates for the Jurassic–Early Cretaceous magmatic arc segments in NE Asia.

Location	Time (Ma)	Paleolatitude (°)	Error (°)	Paleo-elevation (km)	Error (km)	MAT (°C)	Error (°C)
JDP	120	36.0	5.1	4.0	0.7	7.2	6.4
JDP	130	37.5	2.3	3.9	0.6	4.6	5.3
KP	110	35.7	3.2	4.2	0.4	3.4	4.9
KP	165	33.7	6.6	3.8	0.3	6.6	4.7
KP	170	35.6	7.1	3.8	0.3	6.7	4.8
KP	179	29.5	5.5	3.1	0.6	12.8	5.5
LDP	120	40.5	5.1	3.9	0.5	6.1	5.6
LDP	130	42.0	2.3	3.9	0.5	3.3	4.9
NGXR + EGN	120	49.7	5.1	3.7	0.5	2.8	5.5
NGXR + EGN	130	51.2	2.3	3.6	0.7	1.2	5.6
NGXR + EGN	140	49.6	4.3	3.6	0.7	0.2	5.8
NGXR + EGN	141	44.0	3.2	3.6	0.7	2.5	5.7
NGXR + EGN	160	42.7	3.2	4.4	0.3	−0.6	4.7
NGXR + EGN	165	47.7	6.6	4.4	0.3	−0.8	5.4
NGXR + EGN	170	49.6	7.1	4.4	0.3	−1.0	5.5
NGXR + EGN	179	41.2	5.5	4.4	0.3	3.8	5.2
NTHM	120	38.7	5.1	4.3	0.6	4.6	6.0
NTHM	130	40.2	2.3	4.3	0.6	0.7	5.7
NTHM	140	38.6	4.3	4.0	0.6	2.4	5.8
NTHM	141	33.0	3.2	4.0	0.6	4.1	5.7
NTHM	147	23.3	3.9	4.0	0.6	5.1	6.0
STHM	130	37.5	2.3	3.1	0.6	8.0	4.9
SB	110	45.5	5.1	2.3	0.6	8.8	4.8
SGXR	120	45.0	5.1	2.7	0.4	9.4	4.4
SGXR	130	46.5	2.3	2.7	0.4	7.0	3.8
SGXR	140	44.9	4.3	2.7	0.4	6.0	4.0
SGXR	141	39.3	3.2	3.0	0.7	6.7	5.2
SGXR	147	29.6	3.9	3.0	0.7	8.1	5.3
SGXR	153	36.5	2.1	3.0	0.7	9.5	5.1
SGXR	155	41.9	5.1	3.0	0.7	7.9	5.4
SGXR	160	38.0	3.2	3.0	0.7	7.2	5.2
YSM	120	41.0	5.1	4.4	0.4	3.5	5.5
YSM	130	42.5	2.3	4.3	0.5	0.8	5.5
YSM	140	40.9	4.3	4.3	0.5	0.4	5.6
YSM	141	35.3	3.2	4.3	0.5	2.2	5.5
YSM	147	25.6	3.9	3.9	0.5	4.7	5.5
YSM	153	32.5	2.1	3.9	0.5	6.4	5.1
YSM	155	37.9	5.1	3.9	0.5	5.1	5.4
YSM	160	34.0	3.2	4.4	0.6	2.3	5.7
YSM	165	39.0	6.6	4.4	0.6	2.6	6.1
YSM	170	40.9	7.1	4.4	0.6	2.6	6.2
ZGR + JR	110	45.2	5.1	3.4	0.8	3.9	6.1
ZGR + JR	179	36.7	5.5	3.1	0.7	11.1	5.6

For abbreviations see Table 1.

## DISCUSSION

### Orogeny drove the development of high-relief landscapes

**Figure 2.**
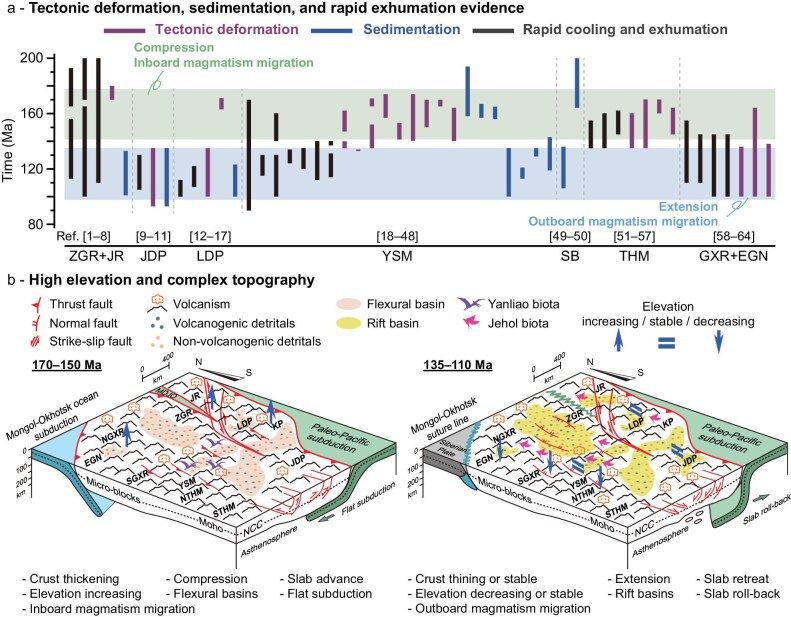
Tectonic transition from compression to extension in NE Asia. (a) Summary of major tectonic deformation, sedimentation, and rapid cooling and exhumation events across the Jurassic–Early Cretaceous of NE Asia. References and details can be found in Table S5. (b) Spatio-temporal reconstruction of tectonic evolution during the Jurassic–Early Cretaceous period. MDJO–Mudanjiang Ocean, other abbreviations are the same as in Fig. 1.

**Figure 3.**
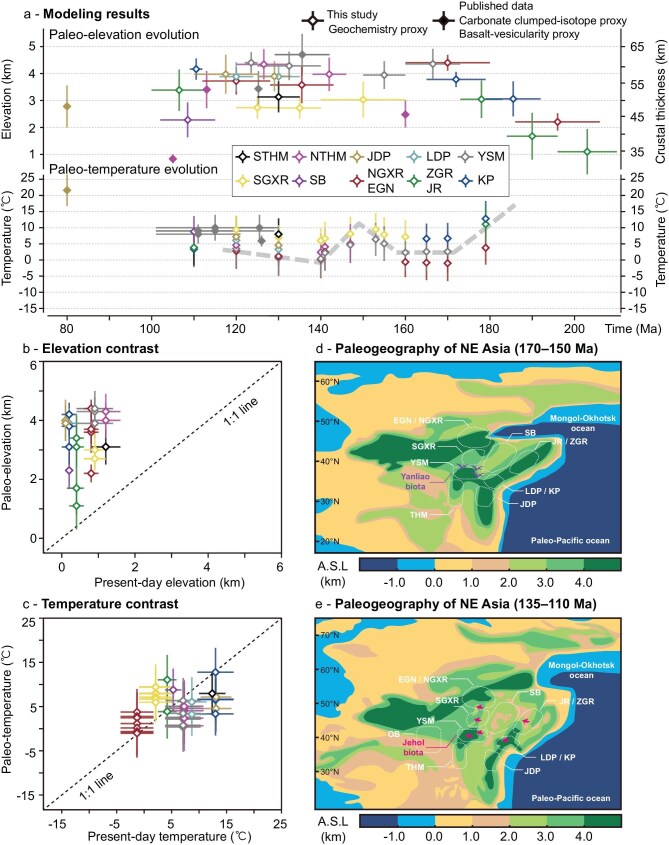
Reconstruction of Jurassic–Early Cretaceous paleo-elevations and paleo-temperatures, comparison with present-day conditions, and paleogeography. (a) Paleo-elevation and paleo-crustal thickness, as well as paleo-MAT evolution across the Jurassic–Early Cretaceous of NE Asia. Other paleo-elevation and paleo-temperature estimates: Zhang *et al.* [11], Zhang *et al.* [28], Wen *et al.* [27], Xia *et al.* [29], Amiot *et al.* [78], and Amiot *et al.* [66]. (b, c) Comparison of paleo-elevations and paleo-MATs during the Jurassic–Early Cretaceous period with present-day elevations and MATs across the studied arc segments. The reconstructed paleo-elevation and present-day elevation uncertainties are reported as the median absolute deviation and standard deviation, respectively. Uncertainties in the reconstructed paleo-MAT account for the combined uncertainties in paleo-latitude and paleo-elevation data. Present-day MAT uncertainties are expressed as the standard deviation of MAT data recorded between 1980 and 2024 by meteorological stations across the examined paleo-arc segment. (d, e) Paleogeography reconstruction in the Middle–Late Jurassic and Early Cretaceous, modified after Scotese *et al.* [16,41]. The abbreviations are the same as in Fig. 1.

Whole-rock chemical proxies from the late Early Cretaceous (∼133–120 Ma) Yanshan Mountains and Northern Taihangshan indicate paleo-elevations of 4.4 ± 0.4 km and 4.3 ± 0.6 km, respectively (Fig. 3a; Table 1), both of which are slightly higher than, but within uncertainties consistent with, the paleosol carbonate clumped-isotope estimates of 2.8–4.1 km and 2.7–4.0 km [11,27], assuming regional terrestrial thermal lapse rates of 4.0–6.0°C/km in East Asia [11,27,28,43]. Moreover, whole-rock proxy results from the Early Cretaceous Yanshan Mountains indicate paleo-elevations of 4.3 ± 0.5 km (Fig. 3a; Table 1), which are consistent with vesicularity-based paleo-altimetry estimates of 4.7 ± 0.8 km from coeval basalts in eastern Inner Mongolia, northwest of the Yanshan Mountains [29]. Paleo-elevation estimates based on whole-rock chemical proxies represent the median paleo-elevation of individual arc segments on a broad regional scale (∼400–500 km in this study), whereas paleosol carbonate clumped-isotope paleo-altimeters sampled from the major intermontane basins likely reflect the paleo-elevation on a localized regional scale (∼100 km for published data in this region). The slight differences of paleo-elevation estimations based on the two methods likely involve variations in elevation along the magmatic arc segments and differences in the locations of the analyzed samples. Assuming same regional terrestrial thermal lapse rates of 4.0–6.0°C/km in East Asia [11,27,28,43], the paleosol or lacustrine carbonate clumped-isotope paleo-altimeter results also indicate paleo-elevations of 1.5–2.3 km for the Middle Jurassic–late Early Cretaceous South Ordos Basin, 2.0–3.0 km and 0.7–1.0 km for the Late Jurassic and mid-Cretaceous Northern Taihangshan and >2.0 km for the early Late Cretaceous Jiaodong Peninsula (Figs 1b and 3a) [27,28,43]. Evidence of permafrost in the North Ordos Basin indicates 3.0–4.0 km paleo-elevations in the Early Cretaceous (Fig. 1b) [44]. In summary, our paleo-elevation estimates along with published paleo-elevation data indicate that a high-elevation complex landscape dominated the NE Asian continental margin during the Middle Jurassic–Early Cretaceous (Figs 2b, 3d, and e; Table 1). Hereafter we shall refer to this large-scale topographic feature as the East Asian Highland.

Several regions of NE Asia experienced significant crustal thickening and elevation increases during the Early–Middle Jurassic (Figs 2, 3a, and d; Table 1), which can be attributed to coeval multi-plate convergence processes, including the scissor-like style closure of the Mongol-Okhotsk Ocean in the northwest [45,46] and the closure of the Mudanjiang Ocean between the Songliao and Jiamusi blocks in the east [47], as well as to the subduction of the Paleo-Pacific Plate in the east [40]. The final closure of the Mongol-Okhotsk and Mudanjiang oceans during the Mid–Late Jurassic [45–47], combined with the flat subduction of the Paleo-Pacific Plate during the Middle Jurassic to earliest Cretaceous (Fig. 1c–e) [18], collectively built the Middle Jurassic–earliest Cretaceous East Asian Highland (Figs 2, 3a, and d; Table 1). A short-lived extensional episode may have occurred during the Late Jurassic, as suggested by a slight decrease in paleo-elevations in the Mid–Late Jurassic Yanshan Mountains (Fig. 3a; Table 1), likely related to changes in the subduction angle of the Paleo-Pacific Plate [48]. These processes are also evidenced by significant intracontinental shortening, sedimentation patterns, and rapid exhumation across NE Asia (Fig. 2a), including in the Yanshan Mountains [19,49], the Taihangshan [50], the Liaodong Peninsula [51], and the Zhangguangcai Range [52]. During this period, the intermontane flexural basins in the Yanshan Mountains and the Liaodong and Korea Peninsulas [39,51], as well as a foreland basin in the Songliao Basin [53] developed and accumulated both volcanogenic and non-volcanogenic detrital materials.

During the Early Cretaceous, the East Asian Highland exhibited a highly heterogeneous topography with significant elevation differences among the arc segments (Figs 2b, 3a, and e; Table 1). The region experienced extensive crustal stretching triggered by the retreat and roll-back of the Paleo-Pacific slab [18,40], which led to the formation of rift basins and metamorphic core complexes [21,22]. At these times, the Southern Taihangshan, the Southern Great Xing’an Range, the Songliao Basin, and the Zhangguangcai Range-Jiamusi Region exhibited decreasing or relatively low mean elevations (Figs 2b, 3a, and e; Table 1). Given that these regions experienced coeval rapid exhumation, basin rifting, and sedimentation (Fig. 2a) [22,52,54], we speculate that intense crustal stretching led to crustal thinning and mean elevation decrease. However, such intense extensional tectonics may not have significantly affected the Yanshan Mountains, Liaodong-Jiaodong-Korea Peninsulas, and Northern Great Xing’an Range-Erguna Region, which largely escaped significant extension and maintained a relatively thick crust and high elevations through much of the Early Cretaceous (Table 1). Large-scale sinistral strike-slip faulting influenced the eastern segment of NE Asia during the Jurassic–Early Cretaceous [55], and the potential development of restraining bends associated with this faulting may have contributed to regional uplift and relief increase [56]. The rapid exhumation and outboard migration of magmatism controlled the development of rift basins across the East Asian Highland (Figs 1e and 2b) [52,54] and their filling with voluminous volcanogenic and non-volcanogenic detrital materials [53]. Additionally, the significant differences between the Early Cretaceous and present-day elevations (Table 1; Fig. 3b) indicate a substantial decrease in elevation after the Early Cretaceous. This trend is loosely reflected in the accumulation of thicker strata in the Late Cretaceous Songliao Basin and the Eocene–Oligocene Bohai Bay Basin [53,57].

### Implications of high-relief landscapes for terrestrial ecosystem evolution

**Figure 4.**
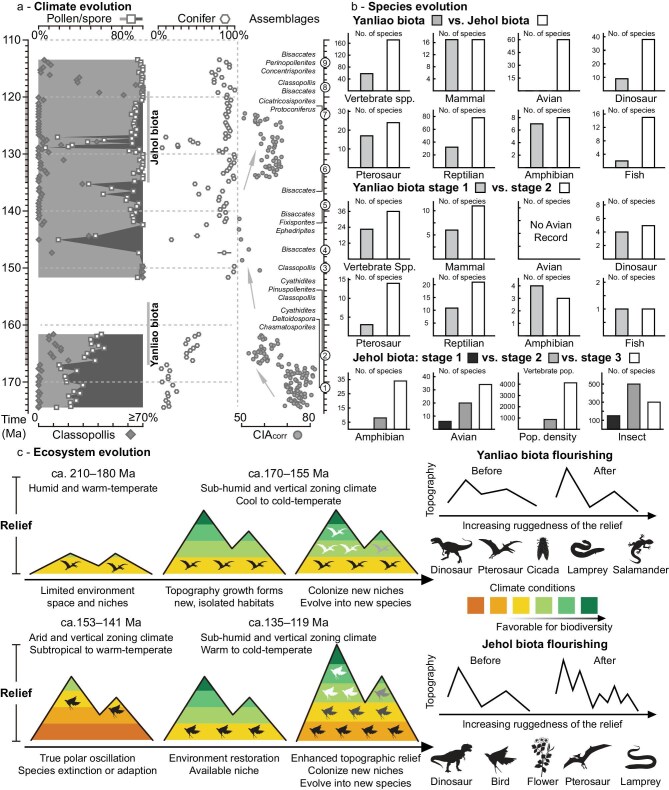
Jurassic–Early Cretaceous ecosystem evolution in NE Asia. (a) Summary of spore/pollen and chemical weathering indices (CIA_corr_) data from major basin successions in the Jurassic–Early Cretaceous Yanshan Mountains. (b) Summary of vertebrate, insect, pollen, and plant species data, along with modeled population density results for the Middle Jurassic–Early Cretaceous Yanshan Mountains. (c) Ecosystem evolution processes during the Late Triassic–Early Cretaceous in NE Asia. Data and references are provided in Table S8.

Our integrated arc-magmatic migration, tectonic deformation, thermochronology, and paleo-elevation data collectively indicate inland migration of topographic rise associated with increasing relief toward the Yanshan Mountains during the Mid–Late Jurassic (∼175–150 Ma; Figs 1 and 2). Recent studies from the eastern Yanshan Mountains indicate a significant increase in gymnosperm pollen (e.g. *Pinuspollenites*) and a decrease in pteridophyte spores in the Mid–Late Jurassic (Fig. 4a) [12]. Concurrently, there is a notable decline in δ^13^C_org_ values, chemical weathering indices (CIA_corr_), and coal seams (Fig. 4a), alongside a facies transition from ever-wet fluvial-delta systems to seasonally active alluvial plains in the Mid–Late Jurassic [12]. These shifts suggest reduced weathering intensity and a decrease in regional mean precipitation and mean temperature. The Yanliao Biota, located in the Yanshan Mountains, generally includes two evolutionary stages, namely the Daohugou and Linglongta stages spanning ∼167–163 Ma and ∼162–156 Ma, respectively (Fig. S6; Table S6). Paleontological evidence from early and late stages of the Mid–Late Jurassic Yanliao Biota shows an increase in vertebrate biodiversity, particularly mammals and reptiles (Fig. 4b). Additionally, floral records from the Yangcaogou (Late Triassic, at least 77 species) and Yanliao Biotas (Mid–Late Jurassic, at least 216 species) in the eastern Yanshan Mountains exhibit pronounced increases in species richness in the Mid–Late Jurassic (Table S8). Taken together, these lines of evidence highlight synchronous shifts in tectonics, topography, climate, and biodiversity. We thus propose a Jurassic ecosystem evolution model for NE Asia in which intense tectonic activity created the East Asian Highland, a 2.5 million km^2^ high-relief landscape (based on the data distribution) with a mean elevation of ∼3.0–4.4 km, and the resulting complex topography significantly influenced regional climate patterns and the flourishing of Mesozoic biomes.

The vertebrate assemblages of the Yanliao Biota comprise a diversity of pterosaurs, the absence of avians, and generally smaller body sizes among dinosaurs, pterosaurs, and mammals, all of which are notably distinct from those of the Jehol Biota [8], and evolved in an environment characterized by forested-mountain-lacustrine and volcanism-influenced features [9]. The dinosaurs from the Yanliao Biota consist mainly of small theropods and some early ornithischians [8,58]. Moreover, the vertebrate assemblages of the Yanliao Biota differ significantly from other contemporary faunas in Eastern Asia (e.g. mamenchisaurids in eastern Asia Fauna) [59] and in North America (e.g. stegosaurus and apatosaurus in Morrison Fauna) [60], all of which have reptiles and mammals with relatively large body sizes. This suggests a unique habitat, geographical isolation, and communication barriers for the Mid–Late Jurassic Yanliao Biota. The occurrence of mountain salamanders and alpine or boreal insects (e.g. Raphidioptera, Prophalangopsidae, and Osmylidae) suggests upland or cool habitats, while cool-water lampreys and fossil woods (e.g. *Xenoxylon* and *Protaxodioxylon*) may reflect relatively cool climatic conditions (Table S7). Paleo-temperature reconstructions for the Yanshan Mountains indicates paleo-MATs from 2.3 ± 5.7°C to 6.4 ± 5.1°C during the Mid–Late Jurassic (Fig. 3a), suggesting a cool to cold-temperate climate comparable to the present-day MAT of 7.1 ± 3.2°C in this region (Fig. 3c). However, some fossil evidence from the Yanliao Biota, including insects, megaflora, and palynology, often points to a relatively warm and seasonal variable climate (Table S7). This discrepancy suggests that different micro/meso-climates coexisted during this period, supporting vertically zoned paleo-environments arising from high topographic relief in this region. Fossil assemblages from the Yanliao Biota, classified into multiple habitat-based communities, also support occupation of diverse micro-environments structured along a vertical gradient [61].

During the Mid–Late Jurassic, the rise of high-elevation and complex topography led to a decrease in regional mean temperature and an increase in both available surface area and vertical elevation gradients (Fig. 4c). These conditions allowed for the coexistence of numerous species with slightly different climatic tolerances, resulting in dense species packing at local scales [4,5]. Both endemic and immigrant species underwent adaptive radiation, evolving and colonizing new available niches (Fig. 4c). Geographic isolation caused by topographic barriers promoted allopatric speciation, giving rise to new endemic species within ‘sky islands’ [4,5]. Moreover, the complex topography prompted vertebrate evolution, driving the development of specialized locomotor adaptations suited to varied ecological environments, such as the early emergence of pennaceous feathers and different winged structure morphologies in theropods [62,63], which further contributed to lineage splitting in dinosaur evolution. Thus, we emphasize that the uplift to form high-elevation and complex landscapes played a critical role in enhancing biodiversity and fostering diverse and dynamic ecosystems in NE Asia during the Mid–Late Jurassic.

The flourishing Mid–Late Jurassic ecosystem experienced a significant disruption during the latest Jurassic–earliest Cretaceous (155–140 Ma) transition, followed by the emergence of the Jehol Biota (135–119 Ma, Fig. 4c). This ecological shift coincides with a regional climate change from relatively humid to highly arid conditions accompanied by rising temperatures [14], as revealed by our paleo-temperature reconstruction (Fig. 3a). Supporting evidence includes the presence of eolian sandstones with large-scale cross-bedding in coeval strata of the Yanshan Mountains [13], a pollen assemblage dominated by drought-tolerant gymnosperms, notably with *Classopollis* comprising ∼90% of the total, and a further decline in chemical weathering indices (Fig. 4a). These changes may be linked to a ∼12° southward latitudinal shift of the East Asian continent between 155 and 147 Ma [15], which likely contributed to the decline or collapse of the Yanliao Biota. Most species became at least locally extinct or dispersed to other regions (e.g. *Fujianvenator* in South China) [64], with only a few taxa persisting through adaptation to newly formed ecological niches [13], as reflected by the taxonomic difference between the Yanliao and Jehol Biotas (Fig. 4b). A subsequent ∼10° northward shift in paleo-latitude of the East Asian continent between 147 and 141 Ma marked the return phase of the true polar wander, facilitating regional environmental recovery and a shift to temperate and sub-humid conditions by 140–130 Ma [15], as indicated by a significant increase in chemical weathering indices (Fig. 4a).

Compared to the latest Jurassic–earliest Cretaceous interval (∼155–140 Ma), the Early Cretaceous (140–119 Ma) exhibits a slight decrease in gymnosperm pollen proportion, dominated by coniferous taxa such as *Picea, Pinus*, and *Podocarpus*, indicative of a warm- to cool-temperate, semi-arid to semi-humid climate (Fig. 4a and Table S8). Relative to the Yanliao Biota, the Jehol Biota contains a greater diversity of vertebrates, including birds, dinosaurs, pterosaurs, and fishes (Fig. 4b). Across the three evolutionary stages of the Jehol Biota, including Dabeigou/Huajiying (135–129 Ma), Yixian (129–126 Ma), and Jiufotang (126–119 Ma), there is a marked increase in species diversity among amphibians, birds, pollen, and insects (Fig. 4b and Table S8). Energy-flow and food-web modeling also suggests a rise in biome densities (Fig. 4b). We therefore propose a new Early Cretaceous ecosystem evolution model for NE Asia, in which a widespread high-relief landscape, locally developed via extensional tectonics of the East Asian Highland under warm to cold-temperate humid climatic conditions, triggered the emergence of the Jehol Biota and contributed to its ecological flourishing (Fig. 4c).

In contrast to the Yanliao Biota, the Jehol Biota thrived in volcanically influenced lacustrine and forest environments and was taxonomically more diverse and geographically more widespread, centered in the Yanshan Mountains but extending across NE Asia (Fig. S6) [6,10]. The emergence of large feathered tyrannosauroids further supports significant ecological and environmental shifts [65]. While many Jehol species appear endemic, suggesting some geographic barriers, other species including dinosaurs, birds, mammals, and amphibians exhibit global distributions and represent relatively early-branching lineages [10]. These factors support the development of an increasingly thriving ecosystem characterized by reduced dispersal barriers, functioning as biodiversity cradles and facilitating widespread radiations. The presence of feathered dinosaurs, cool-water lampreys, cool-adapted insects (e.g. Raphidioptera), and abundant coniferous wood may suggest a relatively cool climatic regime (Table S7). Paleo-temperature reconstructions from paleosol carbonates and vertebrate apatite in the Early Cretaceous Yanshan Mountains also indicate cool climatic conditions, with paleo-MATs ranging from ∼6 ± 2°C to ∼10 ± 4°C [11,66], which are somewhat higher than our Early Cretaceous paleo-temperature estimate of between 0.4 ± 5.6°C and 3.5 ± 5.5°C, but consistent within uncertainties (Table 2). Carbon isotope data from alkanes in the Yanshan Mountains further suggest that the major radiation of the Jehol Biota occurred under relatively cool and dry conditions [67]. Other arc segments, where the Jehol Biota flourished, also show relatively low paleo-MATs from 0.2 ± 5.8°C to 6.1 ± 5.6°C (Table 2), supporting a cold habitat. Ice-rafting deposits, a mixture of cold- and warm-climate palynofloras, and isotopic evidence from Mid–Lower Cretaceous strata in NE Asian basins [68–70], as well as isotopic evidence for continental ice sheets in the mid-latitude Great Xing’an Range region even within the generally super-greenhouse Early Cretaceous [71], further highlight significant topographic differences and a vertically zoned environment (Table S7). This is consistent with the paleo-elevation reconstruction, which indicates both relatively high elevations and pronounced topographic relief, as reflected by significant paleo-elevation differences of 2.1–1.7 km between the Early Cretaceous Yanshan Mountains and the adjacent Southern Great Xing’an Range and Songliao Basin (Table 2). Collectively, these paleontological and paleoclimatic records support a scenario of high-relief, vertically zoned, and mountain-lacustrine paleo-environments.

Following the northward return of the East Asian continent, adaptive radiation of both endemic and immigrated species enabled the colonization of vacant niches (Fig. 4c), while geographic isolation promoted allopatric speciation and the rise of endemic species [4,5]. During the Early Cretaceous, intense crustal extension further enhanced topographic ruggedness, and rock uplift driven by isostatic rebound elevated the relief, with both effects peaking ∼125–120 Ma [40]. Together, these processes expanded the surface area and amplified the ecological niche space, thereby contributing to the exceptional biodiversity of the Jehol Biota. The development of numerous NE–SW-trending rift basins, driven by Paleo-Pacific slab rollback [18,22], not only expanded available ecological space for species dispersal but also enhanced habitat connectivity and reduced dispersal barriers [4,5]. This interpretation is supported by the progressively younger eastward trend of the earliest Jehol fossilized elements in NE Asia, which parallels the eastward progression of basin initiation, sedimentation, and magmatic activities [6]. The eventual decline of the Jehol Biota was likely driven by the sharp rise in global average temperatures during the Mid-Cretaceous [16].

Overall, the gradual building of the Jurassic East Asian Highland and its subsequent differential destruction during the Early Cretaceous along the NE Asia continental margins contributed to a complex topographic configuration over time and space (Figs 2 and 3). Global average temperatures during the Mid–Late Jurassic–Early Cretaceous, ∼18.2–23.9°C, were significantly higher than the present-day average of ∼14.5°C [16]. However, a comparison of the Mid–Late Jurassic–Early Cretaceous and present-day MATs and elevations in the examined region reveal similar climate conditions, but distinct elevational differences (Fig. 3b and c). Therefore, we propose that the rise of the East Asian Highland during the Mid–Late Jurassic–Early Cretaceous provided not only diverse ecological environments, but also relatively cool refugia for diverse species within the context of the warm climate prevailing globally, thereby positioning montane NE Asia as a major biodiversity cradle. Although the Mesozoic East Asian Highland reached elevations comparable to the modern Tibetan Plateau, their topographic relief and global climatic context were markedly different. Such topographic complexity offered opportunities for species to track suitable climates, enhanced their ability to withstand global climate oscillations, and improved the resilience of local ecosystems [4,5]. The uplift of the East Asian Highland likely obstructed the westward transport of Paleo-Pacific-sourced moisture, reducing inland moisture delivery and contributing to a relatively humid climate in at least parts of NE Asia located on the windward side [12,72]. Additionally, coeval vigorous magmatism caused mountain substrates with variable lithological compositions [40], while continued rapid erosion and weathering of magmatic products supplied abundant nutrient elements into the ecosystems [73] and created soil heterogeneity with novel habitats and ecological niches [3]. Mountain building and development of endorheic basins with intense exhumation in the Jurassic–Early Cretaceous NE Asia resulted in the widespread formation of sedimentary covers (Fig. 2a) [6,22] that facilitated the development of soil-dependent rooted flora [2]. Collectively, these geological and climatic processes fostered biodiversity, promoted biosphere evolution, and created conditions beneficial for the flourishing of the Yanliao and Jehol Biotas.

Our results demonstrate that by controlling the patterns of magmatism migration, crustal thickness, elevation variations, and basin formation, the variable dynamics of convergent plate boundaries may also indirectly, but significantly, influence the spatiotemporal evolution of terrestrial ecosystems. This systematic control of plate margin tectonics on ecosystem evolution may provide valuable insights into other dramatically evolving ecosystems worldwide, such as the Tibet-Himalaya-Hengduan region [74], the western North America [1], and the Andes in South America [75].

## MATERIALS AND METHODS

To calculate paleo-elevations and paleo-crustal thicknesses, we compiled published whole-rock major and trace element data as well as zircon U-Pb geochronology data from both intrusive and extrusive rocks sampled between 30°N and 55°N along the continental margin of NE Asia (Tables S1 and S2), and then processed the resulting chemical dataset with GAME [33]. Depending on the diversity of the available chemical data, GAME combines up to 41 mohometer models accounting for the effects of magmatic differentiation, and is primarily designed to be applied to statistically meaningful sample populations representing spatially and temporally limited arc segments in which variations in elevation and crustal thickness can be neglected. Hence, after filtering the samples to ensure that only chemical compositions satisfying the quality requirements and limitations of the approach are employed, we grouped the NE Asian samples based on their location into 10 individual arcs segments that are short enough to minimize the uncertainties resulting from along-strike variations in elevation and crustal thickness, but sufficiently long as to remain unaffected by local disturbances of isostatic equilibrium [33]. To capture possible variation in topography and crustal thickness over time, in most of these arc segments we further distributed the samples by their crystallization age into magmatic activity intervals. To distribute samples that are ambiguously positioned in this scheme, local geological criteria have also been taken into consideration. By this procedure 26 spatially and temporally defined segments were separated. Finally, following the semi-automated standard workflow of GAME [33], we calculated the median paleo-elevation and Moho depth as well as the associated median absolute deviations representative for each of the above-defined arc segments (Table 1).

In order to evaluate the effects of building a high-elevation, high-relief landscape on climate, we estimated the MAT for each arc segment relative to the background of global temperature variations during the Jurassic–Cretaceous in NE Asia. The MAT estimations are based on a two-step approach that accounts for the effects of latitude and elevation on temperature. Considering numerous published paleolatitude data from the Jurassic–Early Cretaceous Yanshan Mountains [15,42], we used the paleolatitude data of the Yanshan Mountains as a reference point. The paleolatitude data of the Jurassic–Early Cretaceous Yanshan Mountains are primarily sourced from published data (refs. 15 and 42 and references therein). Because the collision between the North China Craton and micro-blocks had already finished and formed a unified welded plate during the Mid–Late Jurassic–Early Cretaceous [47,76], we use the present-day latitude differences between the Yanshan Mountains and other arc segments to estimate the Jurassic–Early Cretaceous paleolatitudes for each arc segment. While some studies suggest a ∼20° plate rotation of Jurassic–Early Cretaceous NE Asia [42,77], the resulting latitude uncertainties are negligible compared to the uncertainties reported in published paleolatitude data [77]. The Jurassic–Early Cretaceous MATs at presumed sea level and at the same latitude as each arc segment were estimated using the equator-to-pole temperature gradient model proposed by Scotese *et al.* [16]. All estimations assume a consistent tropical temperature between 15°N and 15°S [16]. Temperature decreases due to elevation were calculated based on the paleo-elevations of each arc segment and terrestrial thermal lapse rates. Regional terrestrial thermal lapse rates in East China are estimated, respectively, to be 4.0–5.0°C/km [37], and 3.75–5.15°C/km [38]. Accordingly, we adopted a terrestrial thermal lapse rate of 3.8–5.2°C/km as a reasonable range for our estimations, although we accept that atmospheric composition differences between present-day and the Jurassic–Early Cretaceous may affect the absolute values, they will not substantially change relative relief estimates. Additionally, we collected present-day MAT data from NE Asia to compare with the Jurassic–Cretaceous MAT data. The present-day MAT values were calculated as the average of MAT data recorded between 1980 and 2024 by meteorological stations across the examined paleo-arc segments.

Further details regarding the sample grouping strategy, data filtering principles, GAME app workflow, paleo-temperature reconstruction procedures, and the utilized whole-rock major and trace element data can be found in the Supplemental Material and Tables S1–S9.

## Supplementary Material

nwag100_Supplemental_Files

## DATA AND MATERIALS AVAILABILITY

All data collected and generated in this study are provided either in the Supplemental Material file or in the Dryad data repository (https://doi.org/10.5061/dryad.7wm37pw6j). The GAME (Geochemical Arc Moho Estimator) software used for paleo-crustal thickness and paleo-elevation reconstruction analyses is available at https://osf.io/bmn8y/.
