# Supplementary material for: Orogeny and topography influenced Jurassic–Cretaceous terrestrial ecosystem evolution in northeastern Asia
Source: Natl Sci Rev. 2026 Feb 13;13(6):nwag100. doi: 10.1093/nsr/nwag100 (PMC13017735; doi:10.1093/nsr/nwag100)
Supplement: nwag100_Supplemental_Files [file nwag100_supplemental_files.zip › Supplemental Material.pdf]

SUPPLEMENTAL MATERIAL for

Orogeny and Topography Influenced Jurassic–Cretaceous Terrestrial Ecosystem Evolution in Northeastern Asia

Nan Wang[1,2], Zhiyong Zhang[1]*, Peter Luffi[3,4,5]*, Zhiheng Li[6], Robert A. Spicer[7,8], Zhiqiang Yu[1], Bo Wan[1], Jing-Jing Zhu[2], Jien Zhang[1], Songjian Ao[1], Dongfang Song[1], Dunfeng Xiang[1,9], Chao Guo[1,10], Wenjiao Xiao[9]*

[1]State Key Laboratory of Lithospheric and Environmental Coevolution, Institute of Geology and Geophysics, Chinese Academy of Sciences, Beijing 100029, China.

[2]State Key Laboratory of Critical Mineral Research and Exploration, Institute of Geochemistry, Chinese Academy of Sciences, Guiyang 550081, China.

[3]Faculty of Geology and Geophysics, University of Bucharest, Bucharest 010041, Romania.

[4]Sabba Stefanescu Institute of Geodynamics, Bucharest 020032, Romania.

[5]Geological Institute of Romania, Bucharest 012271, Romania.

[6]Key Laboratory of Vertebrate Evolution and Human Origins, Institute of Vertebrate Paleontology and Paleoanthropology, Chinese Academy of Sciences, Beijing 10044, China.

[7]Key Laboratory of Tropical Forest Ecology, Xishuangbanna Tropical Botanical Garden, Chinese Academy of Sciences, Mengla 666303, China.

[8]School of Environment, Earth and Ecosystem Sciences, The Open University, Milton Keynes MK76AA, UK.

[9]Xinjiang Research Center for Mineral Resources, Xinjiang Institute of Ecology and Geography, Chinese Academy of Sciences, Urumqi 830011, China.

[10]Department of Geology, University of Vienna, Vienna 1090, Austria.

***Corresponding authors:** Zhiyong Zhang; Peter Luffi; Wenjiao Xiao

E-mail: zyzhang@mail.iggcas.ac.cn; peter.luffi@gmail.com; wj-xiao@mail.iggcas.ac.cn

This Supplementary material contains three sections:

Supplementary Text

Figures S1 to S10

Tables S1 to S9

Supplementary Text

1. GEOLOGICAL SETTING

Regional tectonics

NE Asia and the adjacent regions are traditionally considered to consist of the Erguna, Xing'an, Songliao and Jiamusi micro-continental blocks juxtaposed during the Paleozoic between the Siberia Craton and North China Craton (Figs. 1a and S3) [1,2]. These constitute the easternmost segment of the Central Asian Orogenic Belt representing broad Paleozoic accretion and collision zones, which collided with the North China Craton in the Middle Triassic (Figs. 1a and S3) [1,2]. The diachronous closure of the Mongol-Okhotsk Ocean proceeded from the west to the east during the Jurassic and ended in the Late Jurassic, resulting in the development of the Mongol-Okhotsk Orogenic Belt (Figs. 1a and S3) [3]. The final amalgamation of the Jiamusi and Songliao blocks likely occurred along the Mudanjiang Fault in the latest Triassic–Jurassic (Figs. 1a and S3) [1,4,5], leading to the emplacement of Late Triassic–Jurassic granitoids in the Zhangguangcai Range [4] and the Heilongjiang complex [5]. The architecture of the Mesozoic–Cenozoic NE Asia continental margin was also influenced by superposition of the circum-Pacific tectonic system (Figs. 1a and S3). Affected by subduction of the Paleo-Pacific Plate, the continental margin of NE Asia experienced Jurassic–Earliest Cretaceous compression-dominated intracontinental tectonic deformation and magmatism, with widespread out-of-sequence thrusts and folds in the Yanshan Mountains and its adjacent regions [6,7]. Subsequently, in the Early Cretaceous, NE Asia experienced intensive crustal-scale extension characterized by widespread formation of half-graben and graben structures [8] and metamorphic core complexes [9], as well as abundant volcanism and granitoid intrusion emplacements [5,10]. The extension culminated with the destruction of the North China Craton [9,11].

Magmatism: subduction-related or collision-related?

To calculate paleo-elevations and paleo-crustal thicknesses, it is crucial to determine whether the Mesozoic magmatic rocks distributed in NE Asia are associated with subduction or collision orogeny. The latest Triassic–Jurassic NE Asia was primarily dominated by subduction of the Paleo-Pacific Plate, which led to widespread magmatism [12]. However, two significant continental collision processes

also occurred in the region: the closure of the Mongol-Okhotsk Ocean resulting in the collision between the Siberia Craton and the welded micro-blocks of the eastern Central Asia Orogenic Belt (Fig. S3) [13], whereas the closure of the Mudanjiang Ocean led to collision between the Songliao and Jiamusi blocks (Fig. S3) [1,4]. The diachronous closure of the Mongol-Okhotsk Ocean resulted in the development of linear Carboniferous–Jurassic magmatic belts finalized in Mid–Late Jurassic times [3,13]. We therefore classify the Late Jurassic magmatic rocks distributed across the Erguna Block and the northern Great Xing'an Range as collision-related. The subduction and final collision between the Jiamusi and Songliao blocks resulted in the emplacement of voluminous Late Triassic–Middle Jurassic granitoids in the Zhangguangcai Range [4,5]. Since the final collision occurred during the Mid–Late Jurassic [4,5], we classify the Mid–Late Jurassic granitoids widely distributed in the Zhangguangcai Range as collision-related magmatism. The summarized magmatic crystallization age data reveal an over 1000 km inland migration of the NE Asian magmatic belt during the Jurassic, followed by a reverse migration in the Early Cretaceous (Figs. 1c–1e). Except for the magmatic rocks mentioned above most of the magmatic rocks are related to the Paleo-Pacific Plate subduction [12,14]. Therefore, after identifying the magmatic rocks associated with collision orogenies, all remaining latest Triassic–Cretaceous magmatic rocks are treated as subduction-related.

2. ELEVATION AND MOHO DEPTH ESTIMATION PROCEDURE USING GAME

To estimate paleo-elevations and Moho depths in NE Asia, we followed the strategy and semiautomatic workflow developed by Luffi and Ducea [15], as presented below.

Data filtering

Samples with major element totals significantly outside the 97–101 wt% range are in general considered potentially altered and are discarded by GAME, as they may affect the quality of results. To secure a statistically meaningful number of samples in each arc segment, we nevertheless slightly extended the acceptable range for major element totals to 95–101 wt%. Magmatic rocks from modern subduction zones typically scatter in a differentiation spectrum dominated by subalkaline compositions [16]. Alkaline volcanics are in general less frequent, unevenly distributed among different arcs, and commonly feature exotic trace element signatures. Therefore, samples with total alkali content exceeding the alkaline-subalkaline boundary by more than ~1.5 wt% at any given SiO_2 value are recommended to be removed from the dataset [15]. To retain sufficient samples for calculations, we slightly relaxed this limitation from 1.5 to 1.7 wt%, which is not expected to result in noteworthy extrapolations of the mohometer models. Samples with extreme mafic ($SiO_2 < 45$ wt%) and felsic ($SiO_2 > 80$ wt%) compositions were automatically discarded due to limitations of the modeled

chemical space [15]. Before running GAME, we also excluded some analyses that significantly deviate from common igneous differentiation trends, as defined by major elements versus SiO_2 contents (Harker diagrams). These analyses may represent cumulates or cumulate-dominated rocks and may contribute to abnormal results in the calculations.

Sample grouping strategy

The sample grouping process is essentially an optimization problem, with certain criteria (e.g., number of applicable mohometers and their statistical reliability) driving us to maximize the number of samples in a group, while others (e.g., increasing the spatial and temporal resolution of the defined arc segments) necessitate minimizing it. In the absence of a straightforward or singular solution to this problem, we employed the *Raw estimates* routine of GAME to calculate an median elevation and Moho depth separately for each sample by involving all applicable mohometers that show residuals $\varepsilon_{max} \leq 1$ km relative to the reference elevation model [15]. Albeit having large uncertainties associated, the resulting raw estimates plotted against spatial variables and zircon U-Pb are helpful to optimize the partitioning of the dataset for each examined arc in order to obtain spatiotemporal subdomains that can be considered within error homogeneous in terms of elevation and crustal thickness (Fig. S4). In cases in which such plots did not provide sufficient guidance, we proceeded with geologically motivated—inherently more subjective—decisions on how to group the samples within each arc to achieve meaningful temporal and spatial subdivisions. The resulting division of arc segments is discussed in details in Section 3 below.

Medians + MAD (median absolute deviation) vs. Means + STD (standard deviation)

GAME generates two types of elevation and Moho depth results: Medians ± MAD and Means ± STD. Choosing between these options is crucial. Means with STD are appropriate for normally distributed data with negligible outlier effects. However, in GAME calculations, this may not always hold true due to artificial sampling criteria (MgO bins are delimited artificially, filters exclude data points artificially etc.), which can leave significant outliers that affect precision. While the differences between median and mean estimates are in general minor, STD often exceeds MAD due to these outliers. For this reason, in this study we use Medians ± MAD values.

GAME workflow

The whole-rock geochemical data representing each NE Asian arc segment (Table 1) were processed following the standard GAME workflow summarized below:

1) The data are converted into sets of MgO-binned median values of the chemical parameters

defining the mohometers, using MgO bin sizes of 1 wt%.

2) Punctual elevation estimates are computed by mapping the MgO-binned median values onto the appropriate mohometer models.
3) Punctual estimates falling outside or in poorly modeled domains of the calibration ranges of the corresponding mohometer models (i.e., displaying reference elevation model residuals ε_{max} larger than 1 km, imposed arbitrarily) are removed automatically.
4) Mohometers are filtered based on their performance across all involved MgO bins, which is quantified as the root mean square error of predictions relative to their reference elevation models (RMSE); mohometers exceeding an arbitrarily imposed value of $rmse_{max}$ = 0.5 km are excluded automatically.
5) Some mohometers may occasionally pass the $rmse_{max}$ filter, but still predict spurious results due to the poor analytical or statistical quality of the underlying data; these are excluded manually.
6) Finally, the prediction of each individual mohometer is accepted or rejected based on its overall precision (=MAD) in the evaluated case. We typically used a cut-off value MAD = 5 km for Moho depths (corresponding to ~0.7 km in elevation), which was slightly adjusted on a case-by-case basis, depending on the size of dataset and number of mohometers remaining after the previous filtering steps.
7) Median elevation, median Moho depth, and the associated MAD values are computed from all remaining punctual estimates.

By design, GAME computes elevations (h) directly from compositions and calculates Moho depths (H) as $H = 6.79 \times h + 26.40$, $R^2 = 0.93$, which reflects a correlation characteristic of modern arcs globally that can be attributed to isostatic equilibrium [15]. Crustal thickness (H^{tot}) can be then calculated as $H^{tot} = H + h$.

The robustness of the results obtained with GAME depends on the parameters adjusted is steps 2–6 from above. In this respect, we strived to use ε_{max} and $rmse_{max}$ values as low as possible while maintaining the number of primary data, the MgO bins they populate, as well as the number of simultaneously applied mohometers as great as possible. By tweaking these parameters, the resulting median elevations and Moho depths are not likely to change significantly, but the associated uncertainties may be different. We consider in general robust those Moho depth estimates that have associated MAD values less than 5–6 km and are supported by at least 10 (preferably 15 or more) mohometers. For further details, the reader is referred to section 5.6.1. of Luffi and Ducea [15].

3. THE DIVISION OF ARC SEGMENTS FOR GAME CALCULATIONS

Based on the data filtering principles and arc segment division strategy outlined in Sections 2, we have isolated 10 arc segments in the Jurassic–Early Cretaceous Northeastern Asia. For these arc segments, we calculated 26 paleo-elevation and crustal thickness values across different time periods (see Fig. S5; Table S3).

Jiaodong Peninsula (JDP) arc segment

Jiaodong Peninsula is geographically separated from the Liaodong Peninsula and Taihangshan by the Bohai Bay Basin. The Early Cretaceous Paleo-Pacific subduction resulted in extensive Early Cretaceous magmatism in the Jiaodong Peninsula [17-19]. Based on limited data filtering, we have divided the evolution of the Jiaodong Peninsula arc segment into two distinct periods, ~133–125 Ma and ~125–110 Ma (Figs. S5-4 to S5-5; Table S3).

Korea Peninsula (KP) arc segment: Due to the lack of published data on whole-rock major and trace element data from North Korea, the data available for the Korea Peninsula are primarily collected from South Korea. Consequently, we have divided the Korea Peninsula arc segment based on the data collected from South Korea alone. The southern Korean Peninsula experienced magmatic flare-ups during the Early–Middle Jurassic and the late Early Cretaceous–early Late Cretaceous periods, which are associated with the Paleo-Pacific Plate subduction [20-24]. Based on the available data and potentially significant elevation variations over time by the averaging process, the magmatic activity in the Korea Peninsula arc segment was divided into three distinct periods: ~192–179 Ma, ~179–165 Ma, and ~112–109 Ma (Figs. S5-6 to S5-8; Table S3).

Taihangshan (THM)-Yanshan Mountains (YSM)-Southern Great Xing'an Range (SGXR) - Northern Great Xing'an Range (NGXR)-Erguna Block (EGN) arc segments

The Taihangshan is primarily dominated by early Early Cretaceous magmatism (Figs. 1c–1e). In contrast, the Yanshan Mountains and Great Xing'an Range experienced widespread Middle Jurassic–Early Cretaceous magmatism (Figs. 1c–1e). Meanwhile, the Erguna Block is characterized by continuous Triassic–Early Cretaceous magmatism (Figs. 1c–1e). The Jurassic–Early Cretaceous magmatism distributed in the Taihangshan and Yanshan Mountains is attributed to the coeval subduction of the Paleo-Pacific [12,25]. In the Great Xing'an Range and Erguna Block, Late Triassic–Jurassic magmatism is linked to both the subduction of the Paleo-Pacific Plate and the closure of the Mongol-Okhotsk Ocean [3,26,27]. Conversely, the Early Cretaceous magmatism in these regions is associated with the ongoing subduction of the Paleo-Pacific Plate [26,27].

Geospatially, the plot of individual elevation versus latitude for the Early Cretaceous THM-YSM-SGXR-NGXR-EGN arc segments reveals a noticeable variation in paleo-elevation along the THM-YSM-SGXR-NGXR-EGN direction (Figs. S5-16 to S5-17 and S5-20 to S5-21; Table S3). The southern Taihangshan and southern Great Xing'an Range exhibit relatively lower elevations, whereas the Yanshan Mountains, Northern Great Xing'an Range, and Erguna Block show relatively higher elevations (Figs. S5-16 to S5-17 and S5-20). Based on these results, we have divided the Early Cretaceous THM-YSM-SGXR-NGXR-EGN magmatic arc into four distinct arc segments: the Southern Taihangshan arc segment, the Yanshan Mountains arc segment, the Southern Great Xing'an Range arc segment, and the Northern Great Xing'an Range and Erguna Block arc segment. The plot of individual elevation versus latitude for the Jurassic SGXR-NGXR-EGN arc segments reveals variations (Figs. S5-15 to S5-19; Table S3). The elevation of the Jurassic Southern Great Xing'an Range is lower compared to that of the Northern Great Xing'an Range and Erguna Block (Figs. S5-15 to S5-19; Table S3), as well as the Yanshan Mountains (Figs. S5-11 to S5-12; Table S3). Therefore, we have divided the Jurassic YSM-SGXR-NGXR-EGN magmatic arc into three distinct arc segments: the Yanshan Mountains arc segment, the Southern Great Xing'an Range arc segment, and the Northern Great Xing'an Range and Erguna Block arc segment.

In terms of geological time, considering the limited major and trace element data and the potentially significant elevation variations over time due to averaging process, we have divided the Taihangshan magmatic arc into three segments: Southern Taihangshan (135–125 Ma), Northern Taihangshan (146–138 Ma), and Northern Taihangshan (133–120 Ma). Analyses from some alkaline and mafic magmatic rocks have been excluded from the final elevation calculations (Figs. S5-1 to S5-3; Table S3). For the Yanshan Mountains, the plot of individual elevation versus crystallization age does not show significant variations in elevation (Figs. S5-11 to S5-14; Table S3). After excluding some analyses that fall into the alkaline region or exhibit characteristics of extreme mafic magmatism, we have subdivided the Jurassic–Cretaceous Yanshan Mountains magmatic arc into four segments: Yanshan Mountains (173–160 Ma), Yanshan Mountains (160–150 Ma), Yanshan Mountains (140–125 Ma), and Yanshan Mountains (125–120 Ma). Most analyses from the Southern Great Xing'an Range exhibit typical arc-like igneous differentiation trends (Figs. S5-15 to S5-17). We have divided the magmatism in the Jurassic–Early Cretaceous Southern Great Xing'an Range arc into three time intervals: Southern Great Xing'an Range (160–140 Ma), Southern Great Xing'an Range (140–130 Ma), and Southern Great Xing'an Range (130–120 Ma). North China Craton and Siberia Craton have similar paleolatitudes since ca. 160 Ma [13,28], implying that final closure of the Mongol-Okhotsk Ocean in the eastern

segment occurred after that time. Therefore, excluding the magmatic rocks younger than 160 Ma from the Northern Great Xing'an Range and Erguna Block, we identified four magmatic arc segments active in in different periods: Northern Great Xing'an Range and Erguna Block (206–186 Ma), Northern Great Xing'an Range and Erguna Block (180–160 Ma), Northern Great Xing'an Range and Erguna Block (143–128 Ma), and Northern Great Xing'an Range and Erguna Block (128–112 Ma). These arc segments exhibit significant variations in elevation and crustal thickness (see Figs. S5-18 to S5-21; Table S3).

Songliao Basin (SB) arc segment

The Songliao Basin is one of the major intracontinental rift basins along the eastern Asian continental margin (Fig. 1b) [29], experienced intense magmatism during the late Early Cretaceous syn-rift stage, commonly attributed to slab rollback of the Paleo-Pacific plate [30-32]. Available geochronological and geochemical data for this magmatic episode are mainly derived from the central Songliao Basin, largely obtained through oil-drilling exploration. Based on these datasets, we define a Songliao Basin arc segment with magmatic ages of ~115–102 Ma (Fig. S5-22; Table S3).

Liaodong Peninsula (LDP)-Zhangguangcai Range (ZGR)-Jiamusi Block (JB) arc segments

Geospatially, the Liaodong Peninsula is situated far from the Zhangguangcai Range and Jiamusi Block. The plots of Early Cretaceous individual elevation versus latitude, longtitude, and crystallization age from the Liaodong Peninsula exhibit no significant temporal and spatial variation. Therefore, the Early Cretaceous arc of Liaodong Peninsula can be considered a unique arc segment and its activity can be subdivided into two time intervals, ~135–125 Ma and 124–116 Ma (see Figs. S5-9 to S5-10; Table S3). The collision between the Jiamusi Block and Songliao Block occurred during the Middle–Late Jurassic [4,33,34], and therefore the Middle–Late Jurassic magmatic rocks found in the Zhangguangcai Range are excluded from the calculations. The plot of Late Triassic–Middle Jurassic individual elevations versus crystallization ages shows a significant trend, potentially indicating crustal thickening and topography growth (see Figs. S5-23 to S5-25; Table S3). To account for the significant elevation variations over time, we subdivided the ZGR-JB arc magmatism into three time intervals, ~210–196 Ma, ~196–184 Ma, and ~183–173 Ma (see Figs. S5-23 to S5-25; Table S3). Additionally, we also calculated the elevation for the ~113–100 Ma ZGR-JB arc segment (see Figures S5-26; Table S3).

4. TRADITIONAL INDIVIDUAL Sr/Y AND $(La/Yb)_{CN}$ PROXIES AND DATA FILTERING

Individual chemical mohometers were applied to the intrusive and extrusive magmatic rocks along the northeastern Asia continental margin between 30°N and 55°N. Whole-rock geochemical and geochronological data were compiled from existing publications. Our focus was on magmatism mainly

occurring between 200 Ma and 100 Ma, aiming to capture the building and destruction processes of potential high-topography orogenic belt formed within a subduction setting, characterized by the landward migration of the arc magmatism followed by its retreat [11,12,14].

For Moho depth estimates using individual Sr/Y and $(La/Yb)_{CN}$ proxies, we employed the geochemical data filtering and processing methods outlined by Chapman et al. [35] and Hu et al. [36]. Specifically, samples with SiO_2 of 55–70 wt%, MgO of 1.0–6.0 wt%, and Rb/Sr ratio of 0.05–0.20 were selected and grouped into data subsets based on similar ages and geographic locations (Figs. S7 and S8). Outlier Sr/Y and $(La/Yb)_{CN}$ ratios were identified and removed using the modified Thompson tau statistical method. We then calculated the median Sr/Y and $(La/Yb)_{CN}$ ratios and their standard deviations. Data subsets with Sr/Y standard deviations greater than 10 and $(La/Yb)_{CN}$ standard deviations greater than 5 were discarded. Due to the absence of precise ages or uncertainties in the compiled data, we estimated ages from surrounding plutons and report 5% uncertainties, and subsequently calculated weighted average ages and standard deviations from the data subsets. The corresponding raw data and calculated results are provided in Table S9.

5. EMPIRICAL CORRELATION EQUATIONS AND THEIR LIMITATION FOR Sr/Y AND $(La/Yb)_{CN}$ PROXIES

We utilized a global compilation of geochemical data from magmatic rocks in modern subduction and collision zones, along with corresponding Moho depths and elevations [36]. We recalibrated the direct empirical relationships between global arc-averaged Sr/Y and $(La/Yb)_{CN}$ ratios and Moho depths and elevations using weighted least squares regression (Equations 1–8), to estimate Moho depths and elevations for these samples within subduction and collision setting, using the similar methodologies of Chapman et al. [35], Hu et al. [36], Profeta et al. [37], and Hu et al. [38].

The empirical equations between the median Sr/Y and $(La/Yb)_{CN}$ ratios of magmatic rocks, and average elevations and Moho depths from subduction and collision zones are presented in Figure S9. The calculated regressions show strong correlations with $R^2 > 0.85$, validating the effectiveness of Equations 1–6. However, the models for collision zone based on $(La/Yb)_{CN}$ ratios show relative lower correlation coefficients. The empirical equations for subduction zones are listed as follows (Equations 1–4):

$$Hs = (-0.59 \pm 0.24) + (0.10 \pm 0.01)\, Sr/Y, \quad R^2 = 0.87 \quad (1)$$

$$Ds = (12.34 \pm 2.50) + (1.00 \pm 0.08)\, Sr/Y, \quad R^2 = 0.86 \quad (2)$$

$$Hs = (2.28 \pm 0.15)\log(La/Yb)_{CN} - (2.19 \pm 0.39),\ R^2 = 0.89 \quad (3)$$

$$Ds = (23.19 \pm 1.63)\log(La/Yb)_{CN} - (4.07 \pm 3.90),\ R^2 = 0.88 \quad (4)$$

where "H" is the elevation in kilometers and the subscript "s" denotes subduction zone models. "CN" indicates the values normalized to chondritic values of [39]. The empirical equations for collision zones are as follows (Equations 5–8):

$$Hc = (-0.18 \pm 0.39) + (0.11 \pm 0.01)\,Sr/Y,\quad R^2 = 0.85 \quad (5)$$

$$Dc = (26.35 \pm 2.21) + (0.73 \pm 0.05)\,Sr/Y,\quad R^2 = 0.95 \quad (6)$$

$$Hc = (2.51 \pm 0.55)\log(La/Yb)_{CN} - (3.95 \pm 1.61),\ R^2 = 0.70 \quad (7)$$

$$Dc = (20.05 \pm 5.20)\log(La/Yb)_{CN} - (5.26 \pm 15.09),\ R^2 = 0.62 \quad (8)$$

where the subscript "*c*" refers to the collision zone models. "CN" denotes the values normalized to chondritic values of [39].

All of the above steps are performed using the R programming language, employing relevant functions and libraries, such as "ggplot 2" and "predict" function. The calibrated equations are consistent with those calibrations proposed by Chapman et al. [35], Hu et al. [36], Profeta et al. [37], Hu et al. [38], and Sundell et al. [40]. The uncertainty in predicted paleo-elevations and Moho depths includes contributions from the equation itself (derived from confidence interval–"se.fit", and prediction intervals–"fit.lwr" and "fit.upr") and the standard deviations of Sr/Y and $(La/Yb)_{CN}$ ratios within each data subset. Paleo-elevation estimates exceeding 6 km for subduction systems ($Sr/Y > 65$; $(La/Yb)_{CN} > 30$) and collision zones ($Sr/Y > 50$; $(La/Yb)_{CN} > 40$) and lower than 1 km ($(La/Yb)_{CN} < 8$) for collision zones are considered invalid, as these ranges are not constrained by the data used for calibration. These empirical equations do not require assumption of crust and upper mantle density, thereby reducing uncertainty, as emphasized by Hu et al. [36].

Comparison of predicted paleo-elevations and Moho depths based on the two proxies, Sr/Y and $(La/Yb)_{CN}$, reveals noticeable discrepancies when applied to the same samples (Fig. S10). These differences reflect the relatively large uncertainties associated with estimates derived from a single proxy. Accordingly, integrating multiple proxies of paleo-elevation and Moho depth provides more robust and reliable constraints on crustal thickness and surface elevation.

Supplementary Figures

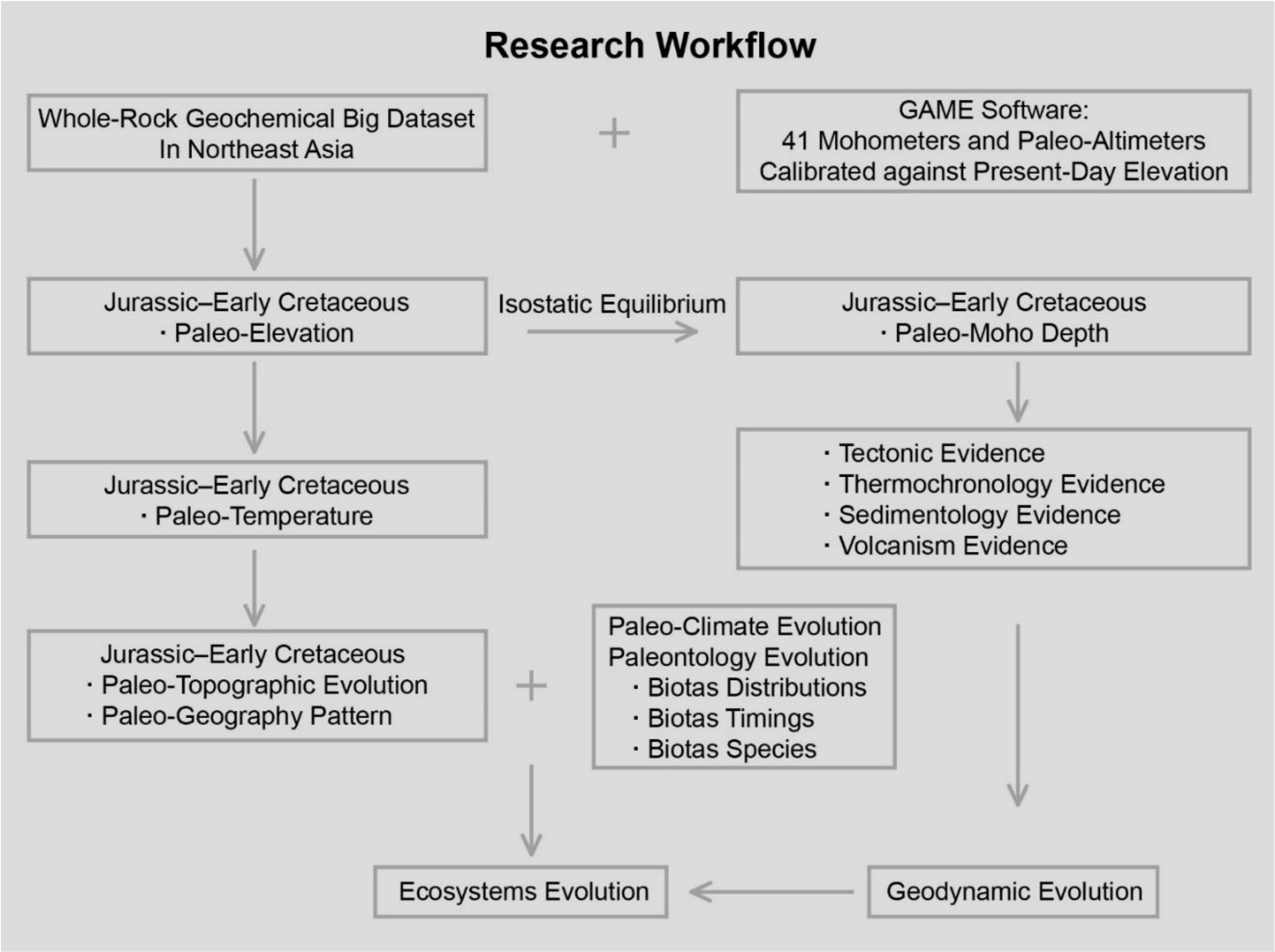

Fig. S1. Research workflow for this study

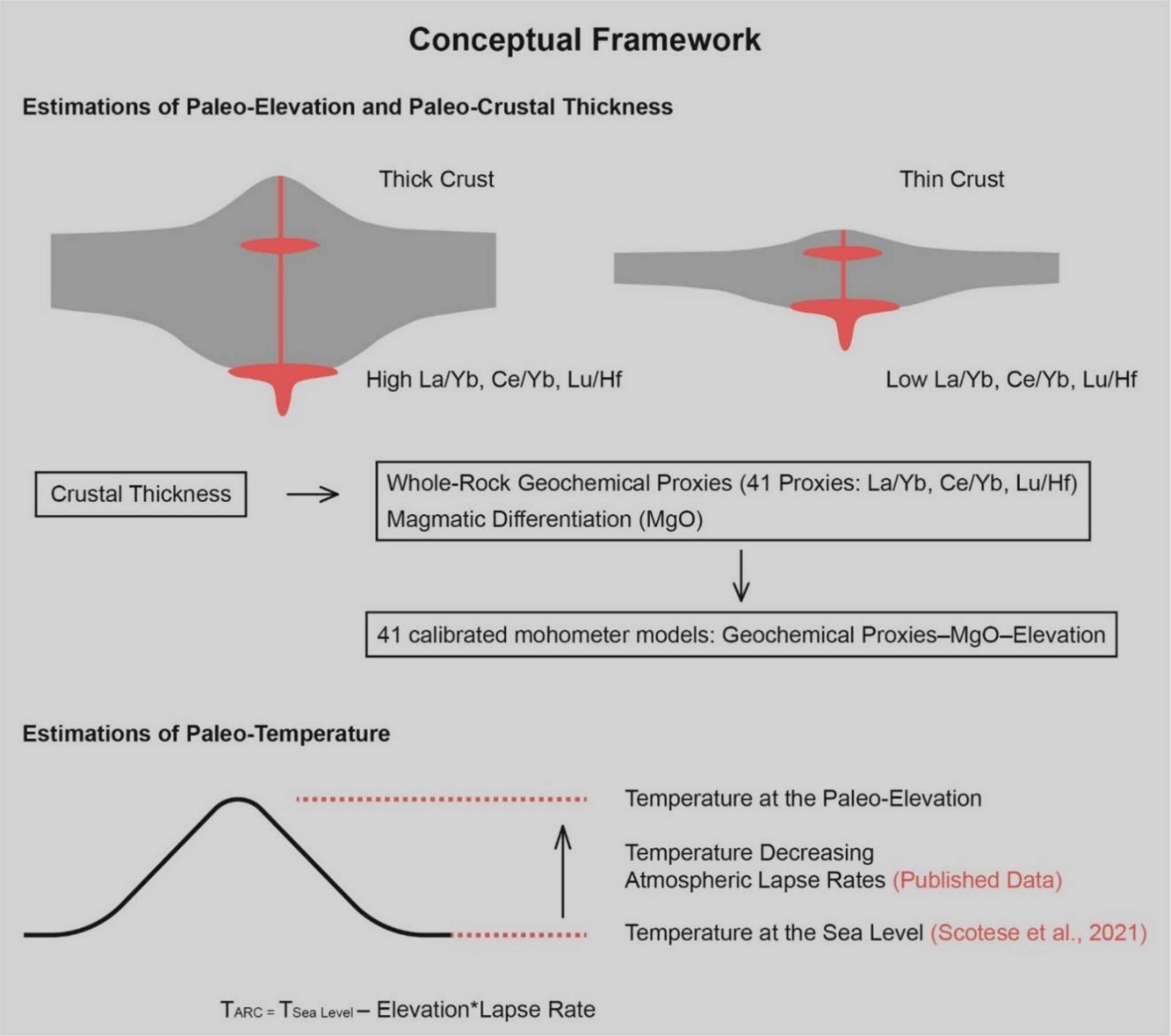

Fig. S2. Conceptual framework for estimations of paleo-elevation and paleo-temperature, modified after Tang et al. [41]

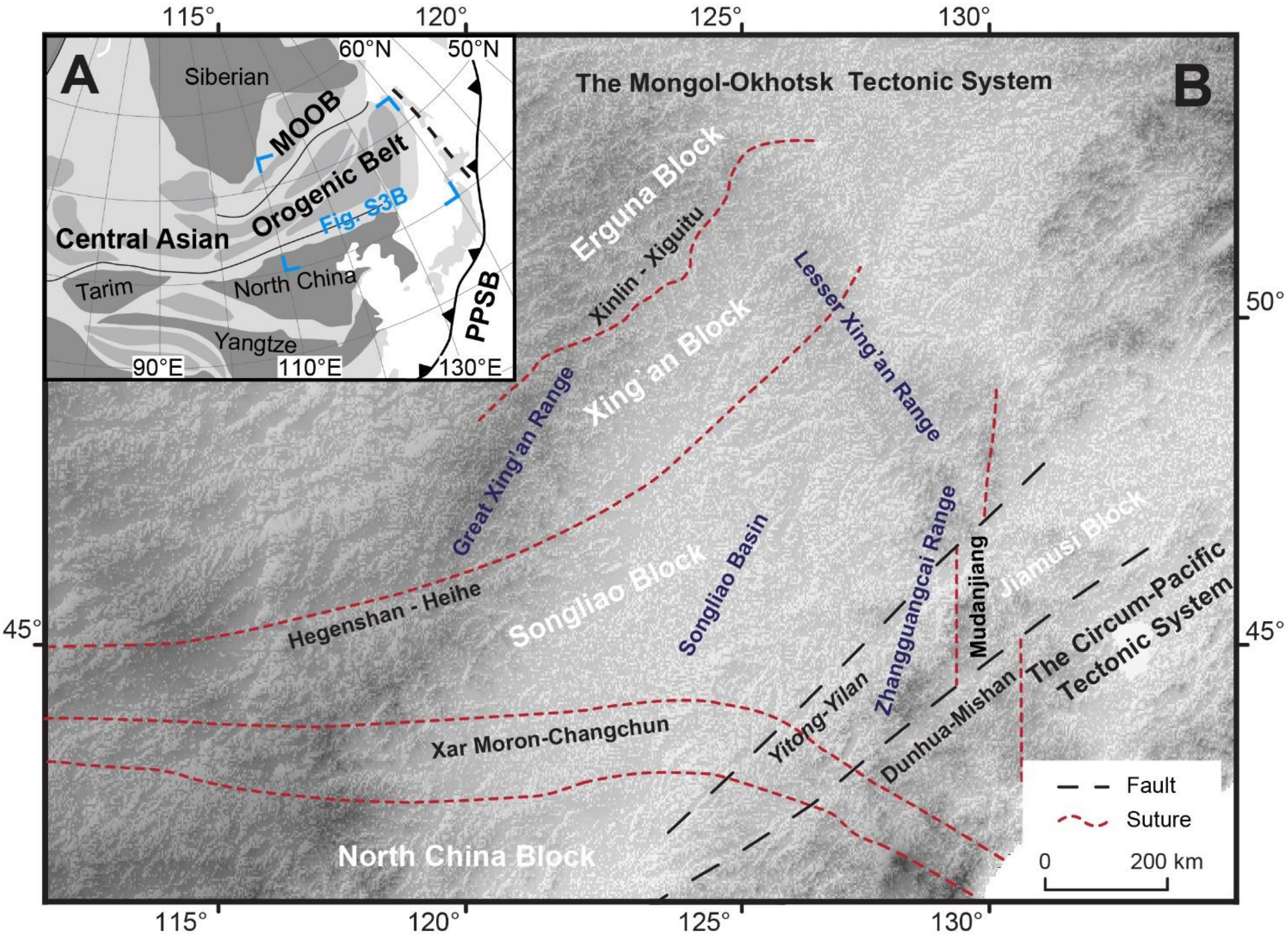

Fig. S3. Tectonic subdivision of northeastern Asia

A: Schematic tectonic subdivision of the Eurasian continent, modified after Liu et al. [1]. B: Regional tectonics and tectonic subdivision of Northeastern Asia, major faults are modified after Wu et al. [5]. PPSB–Paleo-Pacific Subduction Belt; MOOB–Mongol-Okhotsk Collision Belt.

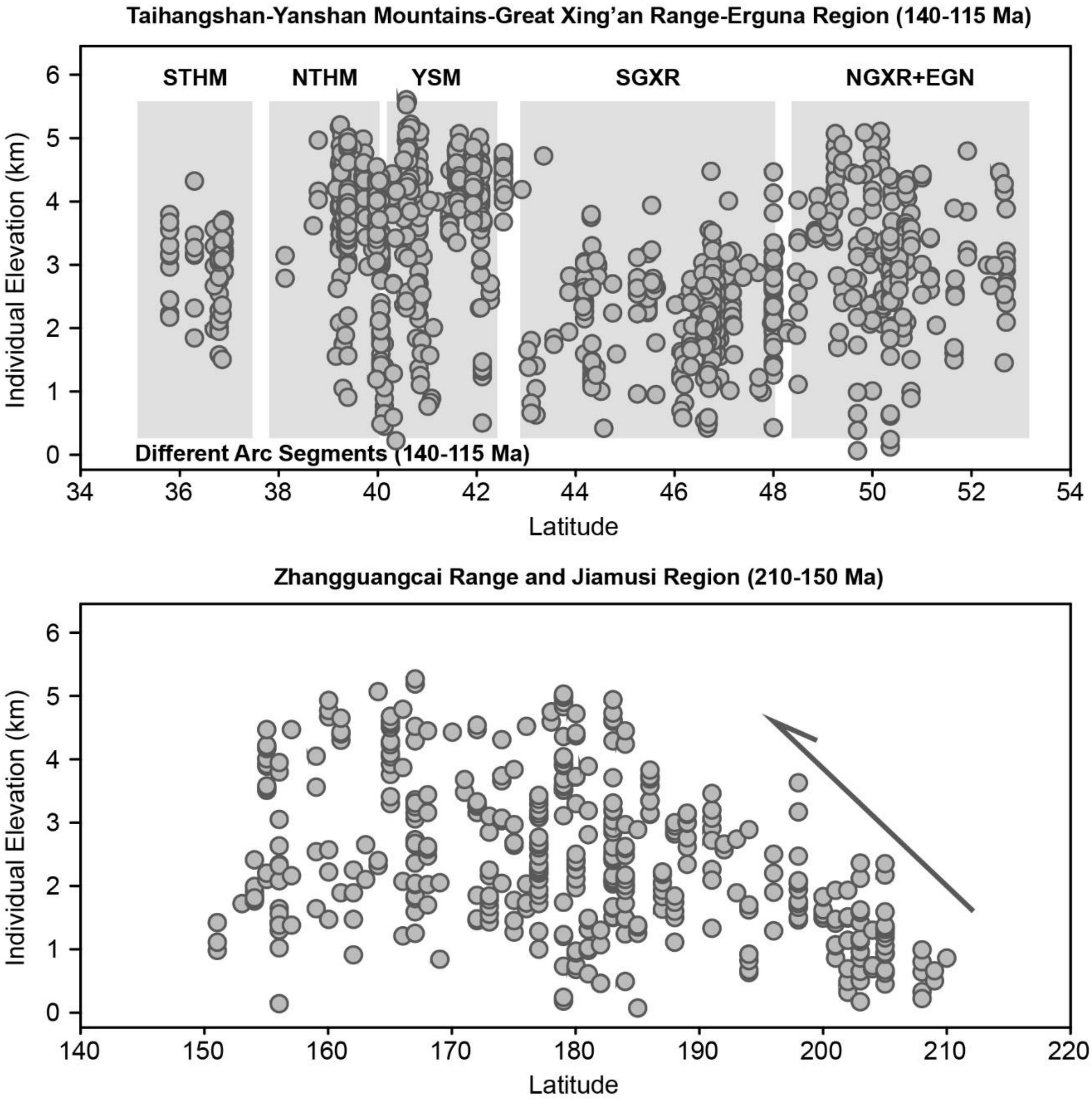

Fig. S4. Individual elevation estimates plotted against latitudes and zircon U-Pb ages

Individual elevations represent the median elevation estimates obtained for the quality-filtered individual samples, computed by using all applicable mohometers that satisfy the imposed εmax limitation.

Figure S5-01 Southern Taihangshan (STHM): 135-125 Ma Magmatism

Individual Elevation vs. Age (135-125 Ma)

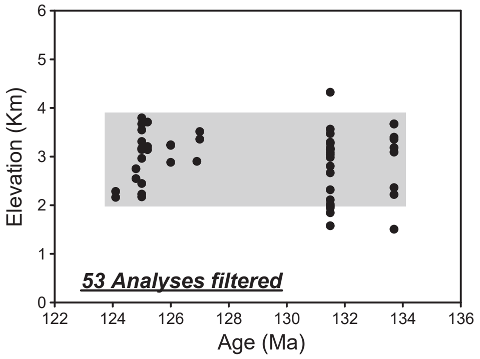

TAS diagram (135-125 Ma)

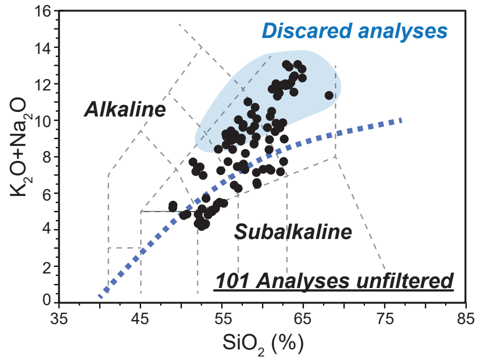

Individual Elevation vs. Longtitude and Latitude (140-115) and (135-125 Ma)

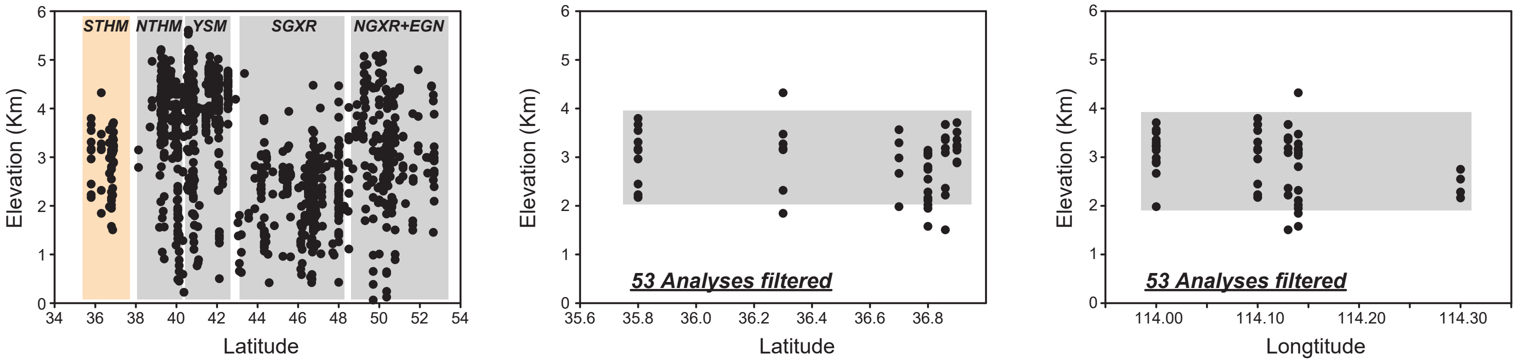

Harker Diagrams (135-125 Ma)

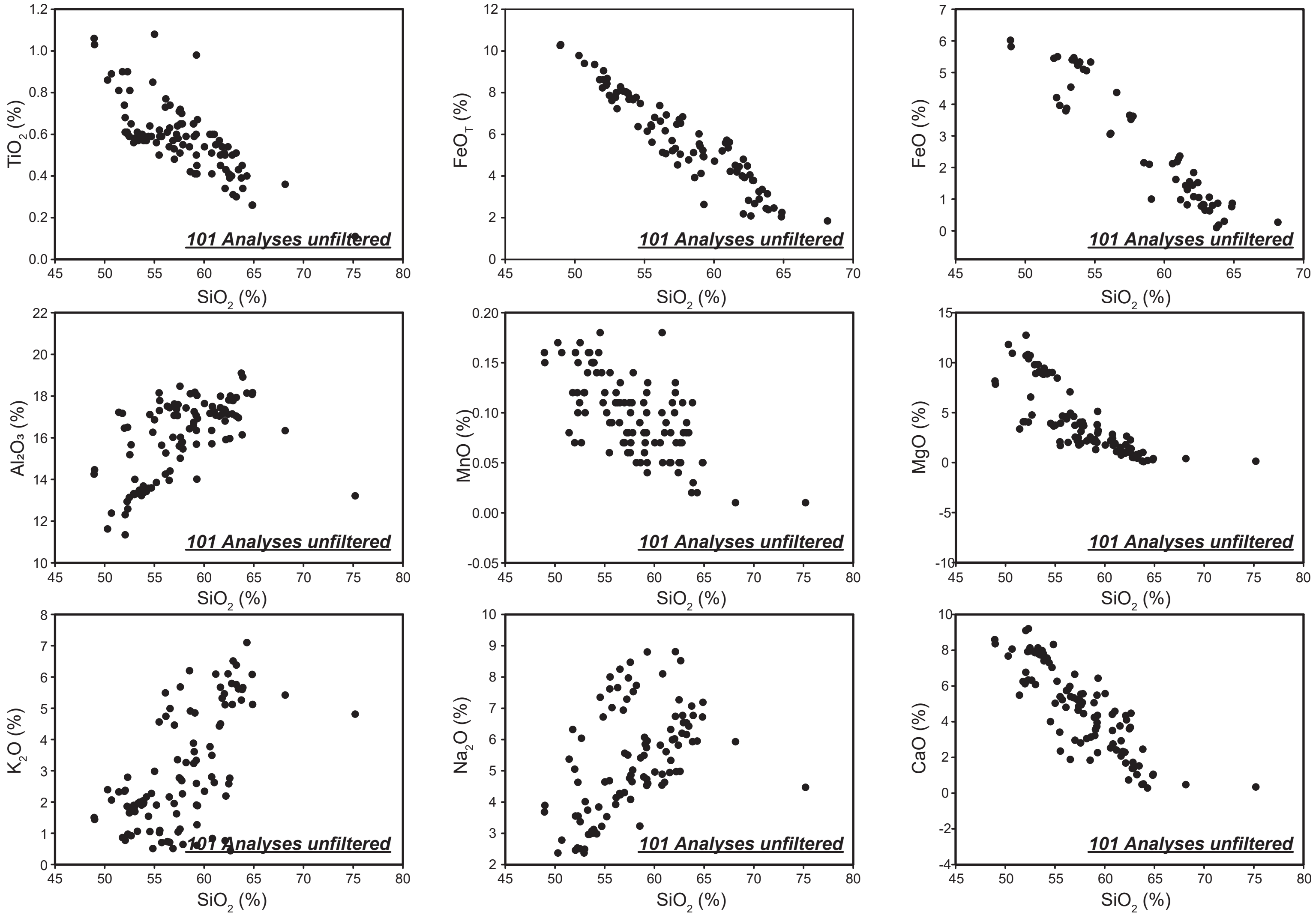

Southern Taihangshan 135-125 Ma Arc Segment GAME Results

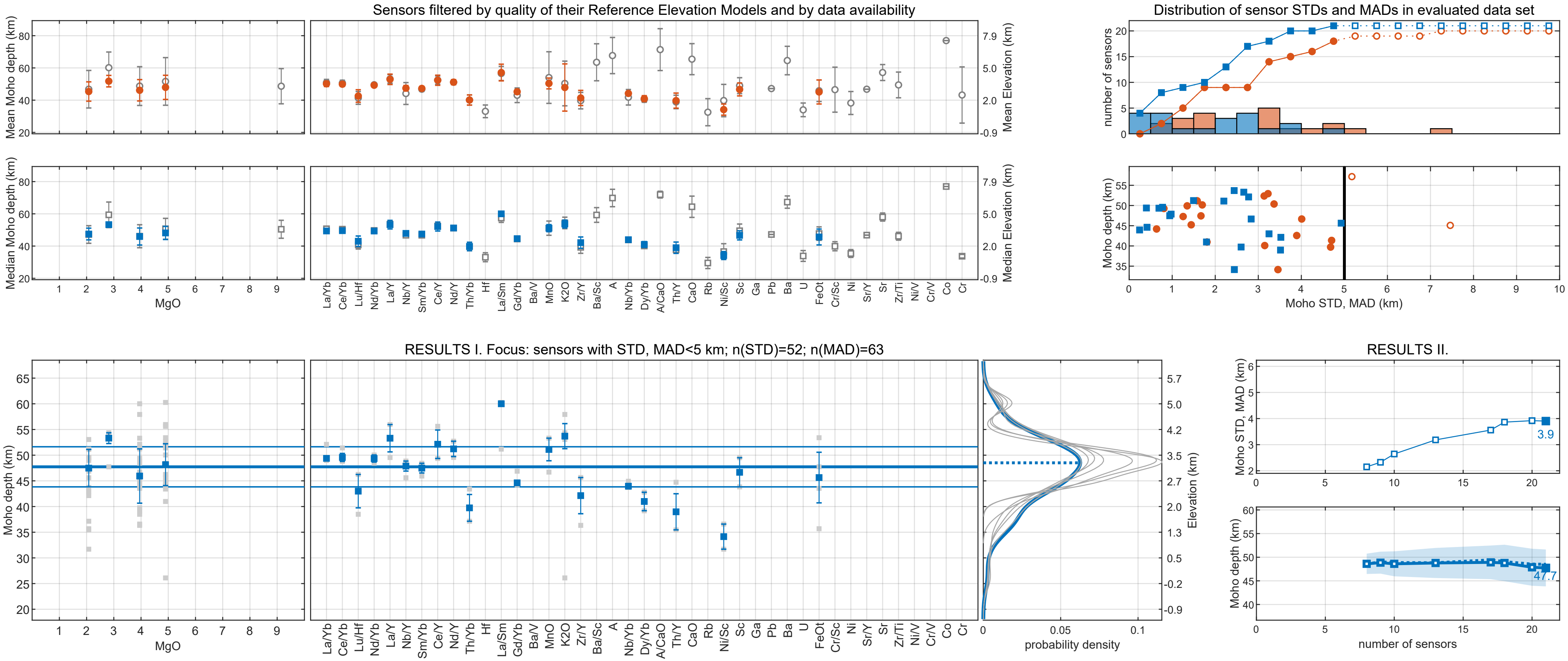

Figure S5-02 Northern Taihangshan (NTHM): 146-138 Ma Magmatism

Individual Elevation vs. Age (146-120 Ma)

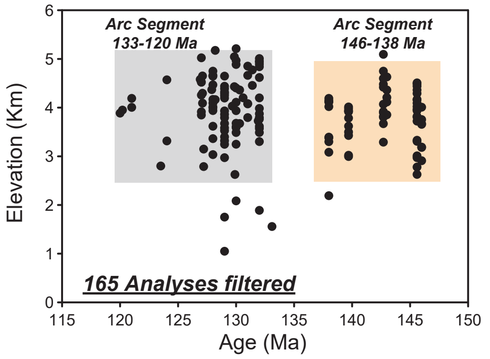

TAS diagram (146-138 Ma)

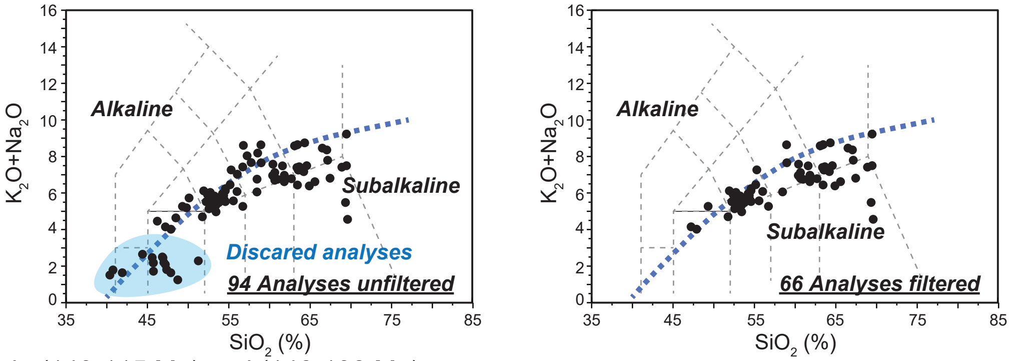

Individual Elevation vs. Longtitude and Latitude (140-115 Ma) and (146-138 Ma)

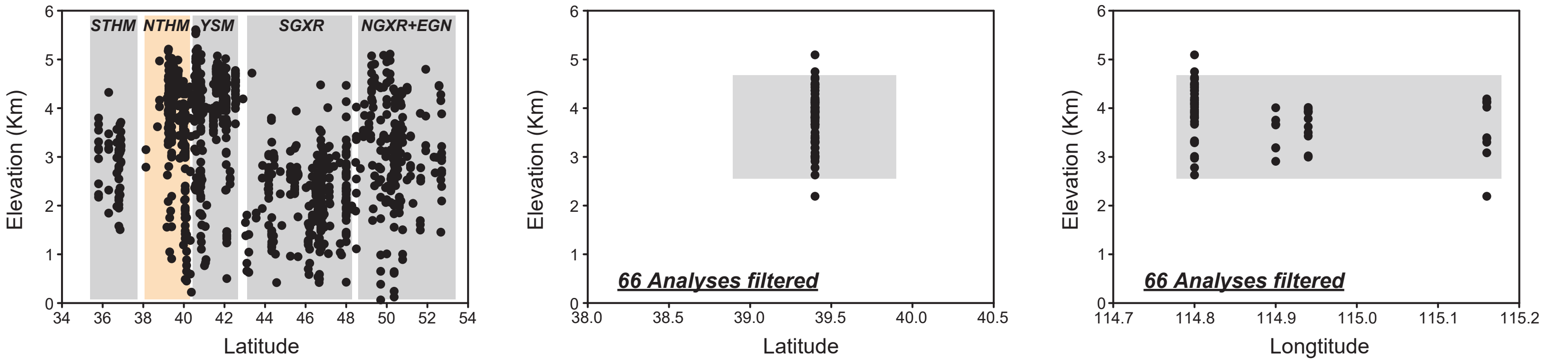

Harker Diagrams (133-120 Ma)

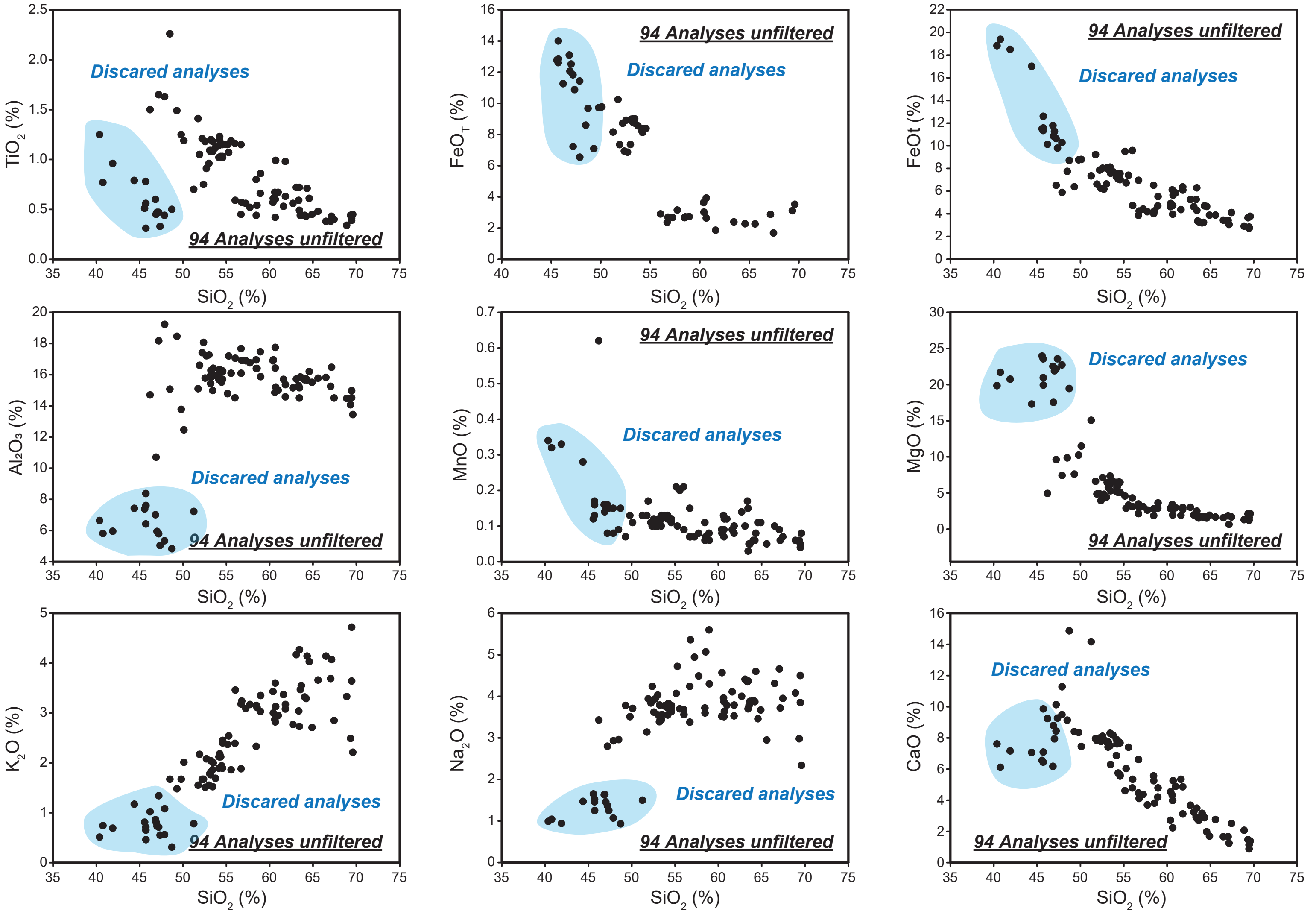

Northern Taihangshan 146-138 Ma Arc Segment GAME Results

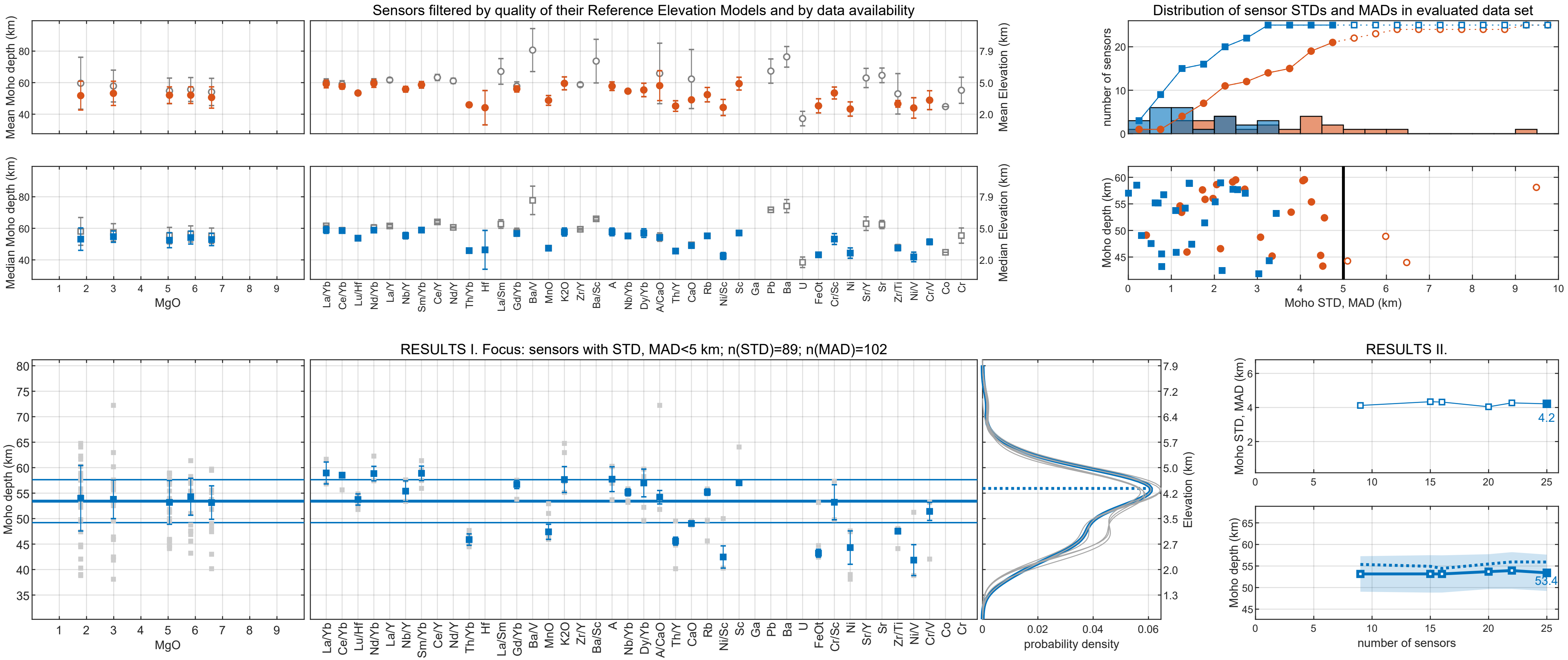

Figure S5-03

Northern Taihangshan (NTHM): 133-120 Ma Magmatism

Individual Elevation vs. Age (146-120 Ma)

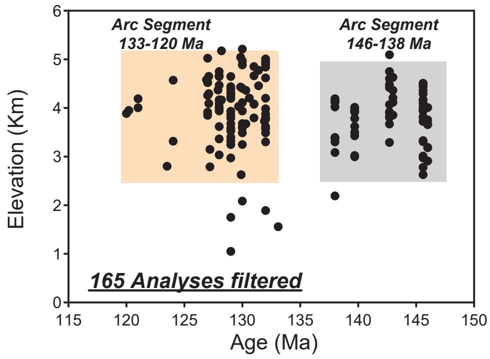

TAS diagram (133-120 Ma)

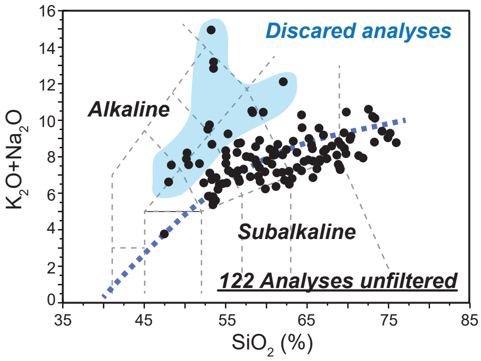

Individual Elevation vs. Longtitude and Latitude (140-115 Ma) and (133-120 Ma)

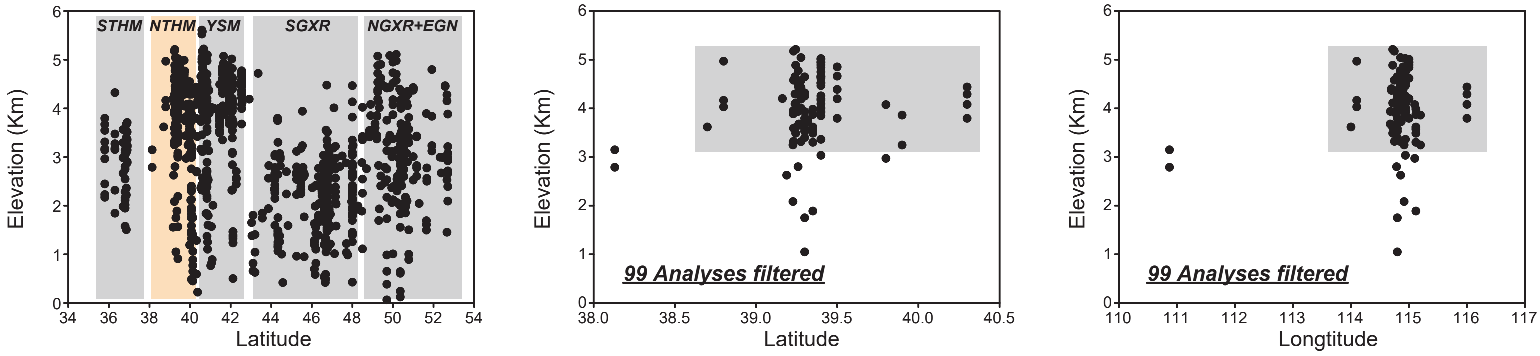

Harker Diagrams (133-120 Ma)

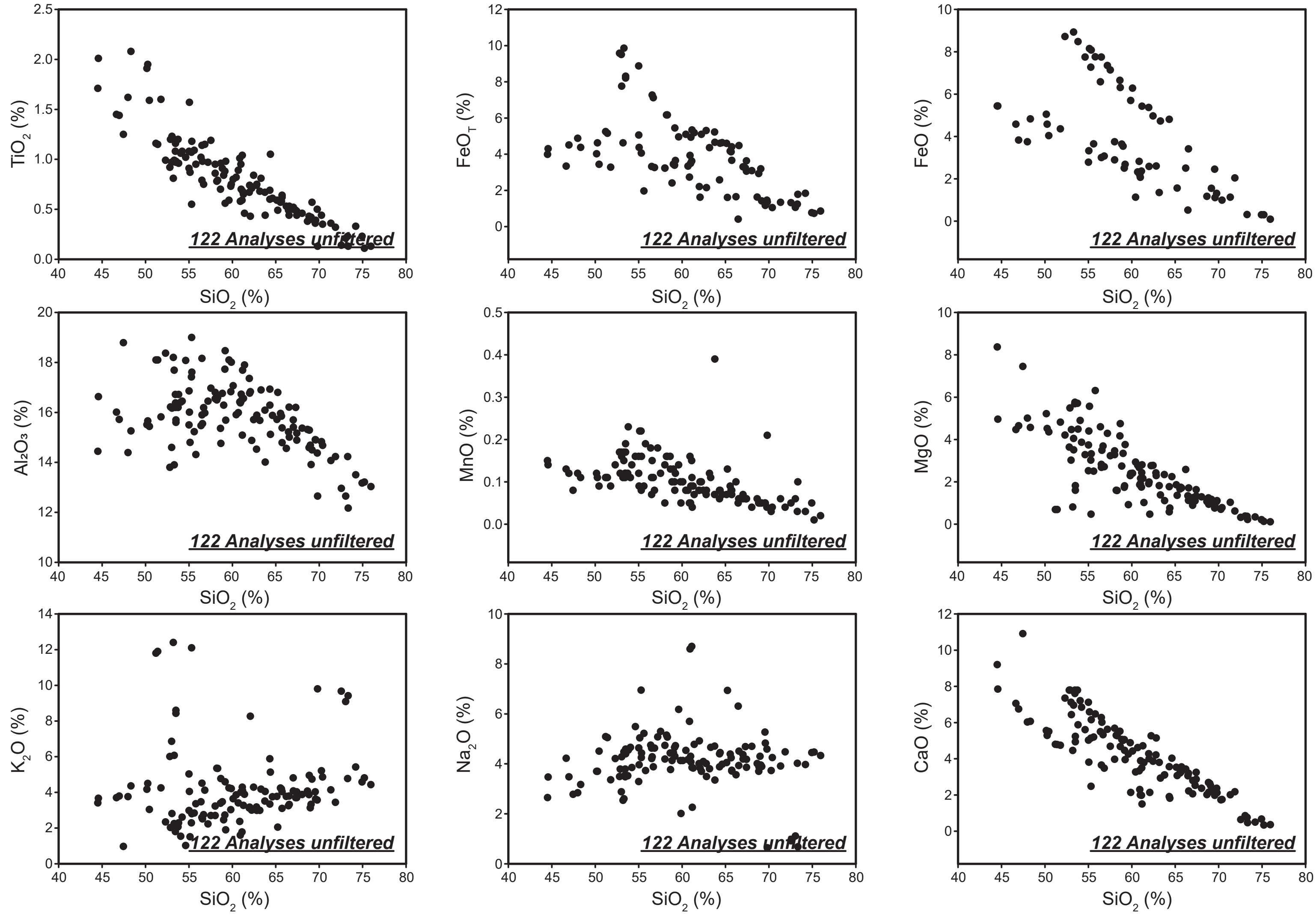

Northern Taihangshan 133-120 Ma Arc Segment GAME Results

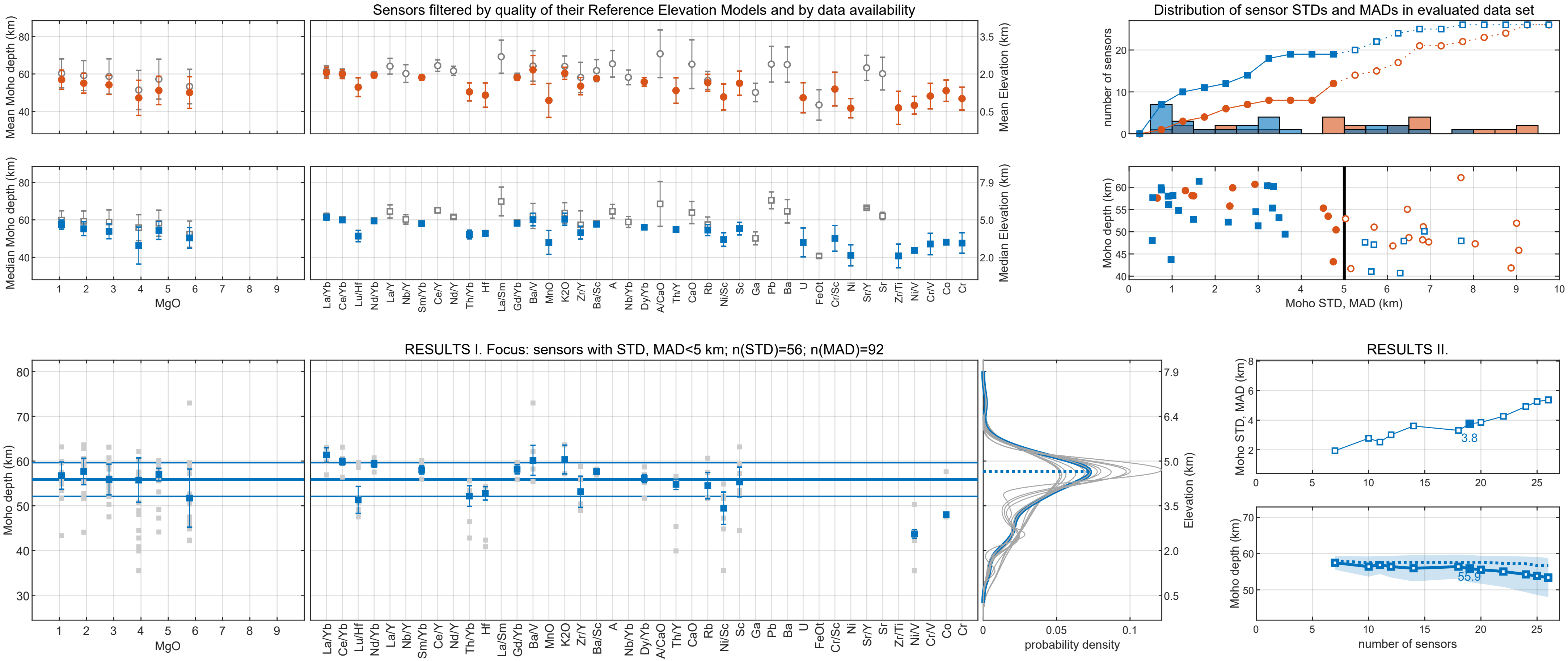

Figure S5-04 Jiaodong Peninsula (JDP): 133-125 Ma Magmatism

Individual Elevation vs. Age (159-106 Ma) and (133-125 Ma)

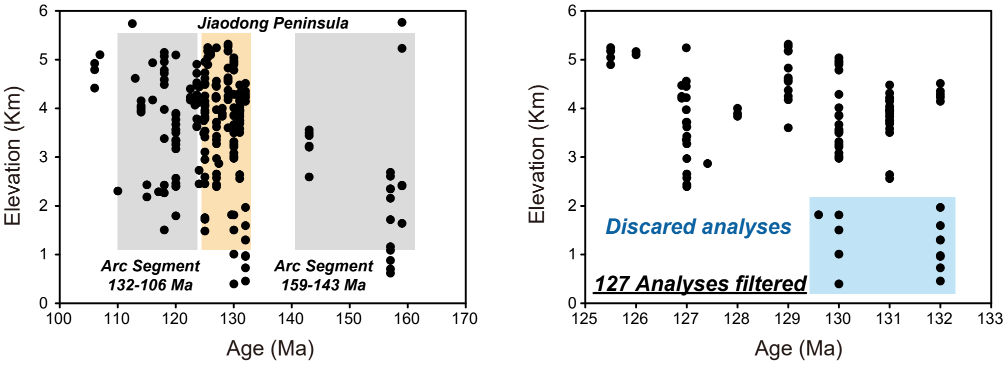

TAS diagram (133-125 Ma)

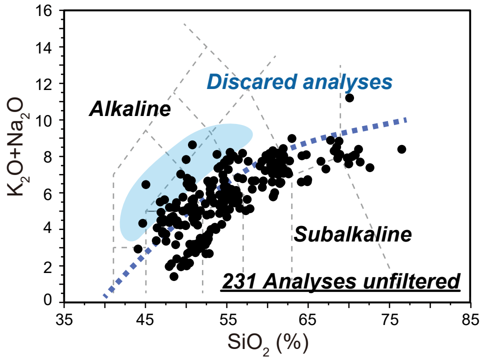

Individual Elevation vs. Longtitude and Latitude (133-125 Ma)

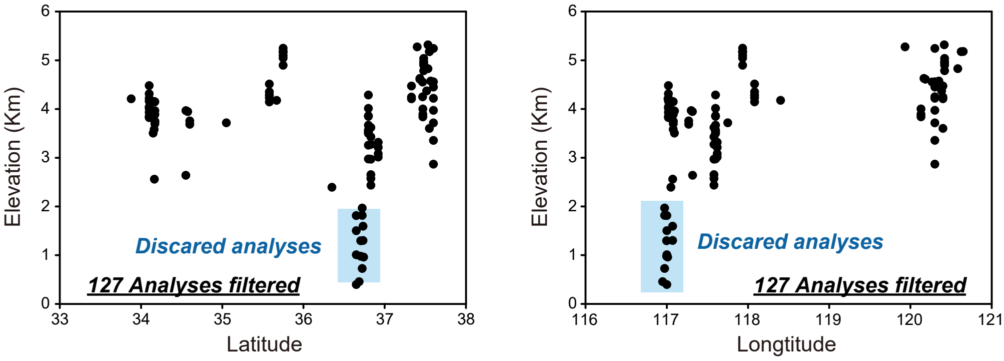

Harker Diagrams (133-125 Ma)

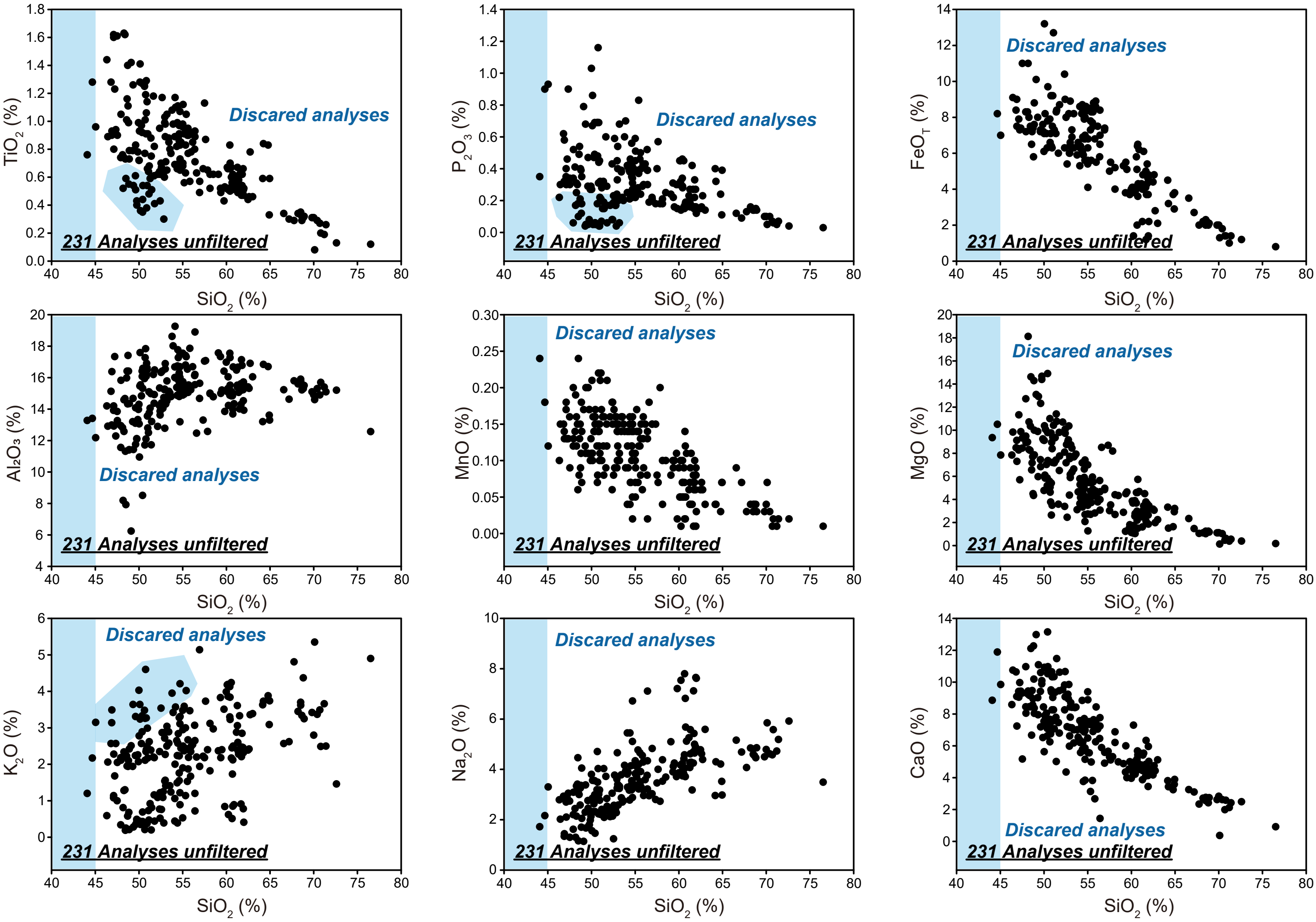

Jiaodong Peninsula 133-125 Ma Arc Segment GAME Results

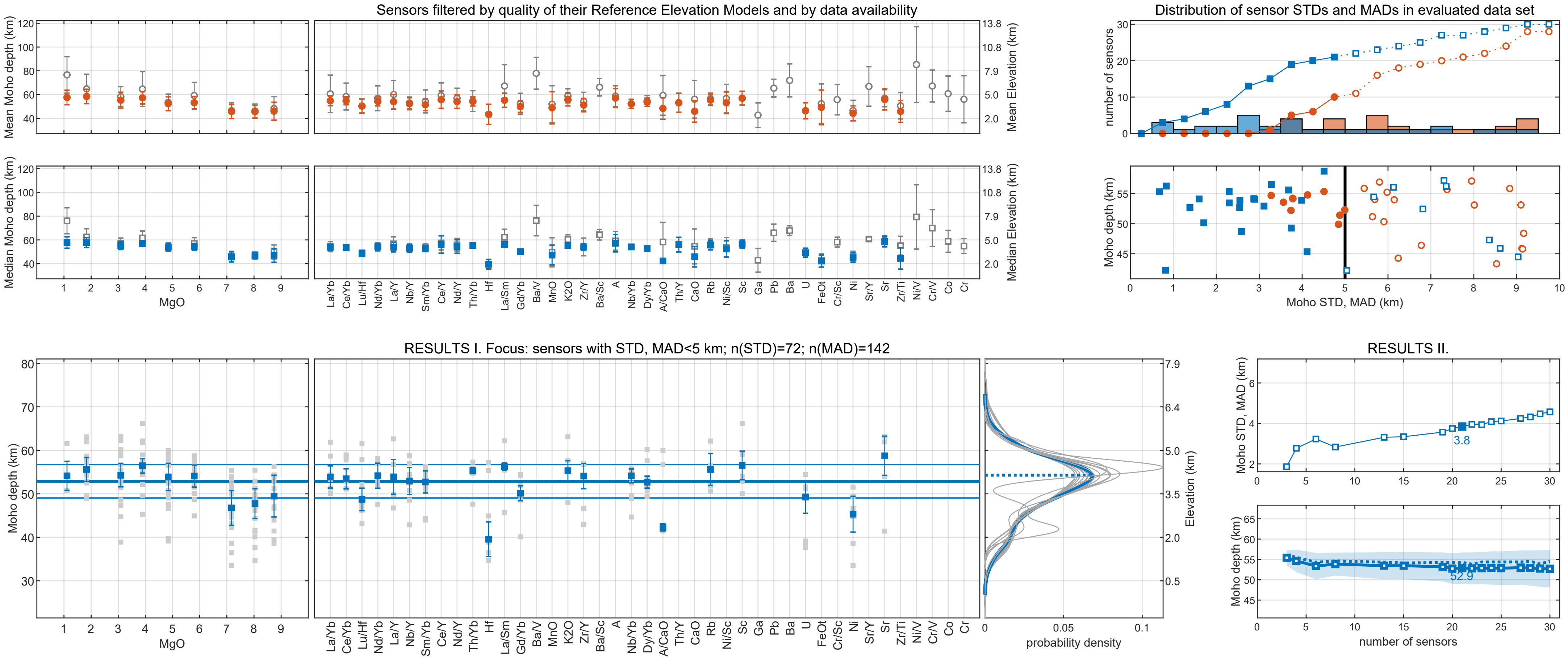

Figure S5-05 Jiaodong Peninsula (JDP): 125-110 Ma Magmatism

Individual Elevation vs. Age (159-106 Ma) and (125-110 Ma)

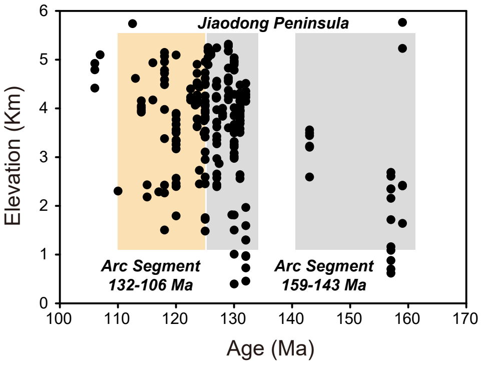

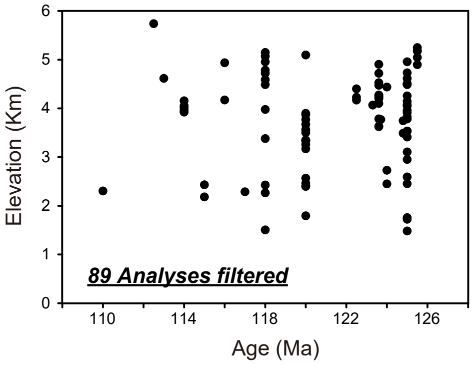

TAS diagram (125-110 Ma)

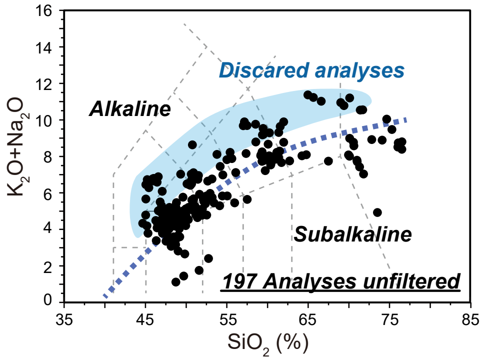

Individual Elevation vs. Longtitude and Latitude (125-110 Ma)

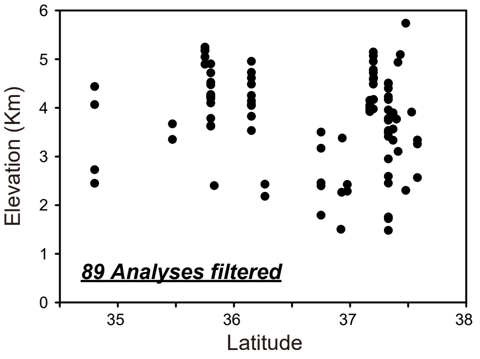

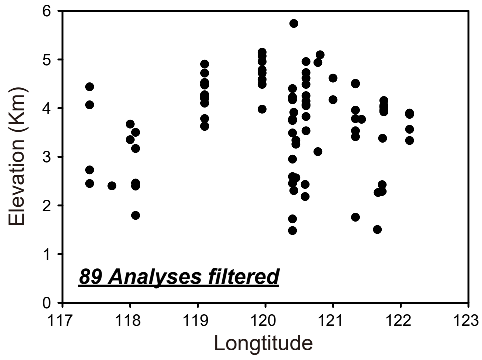

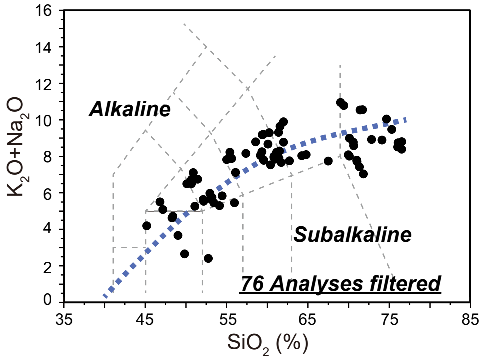

Harker Diagrams (125-110 Ma)

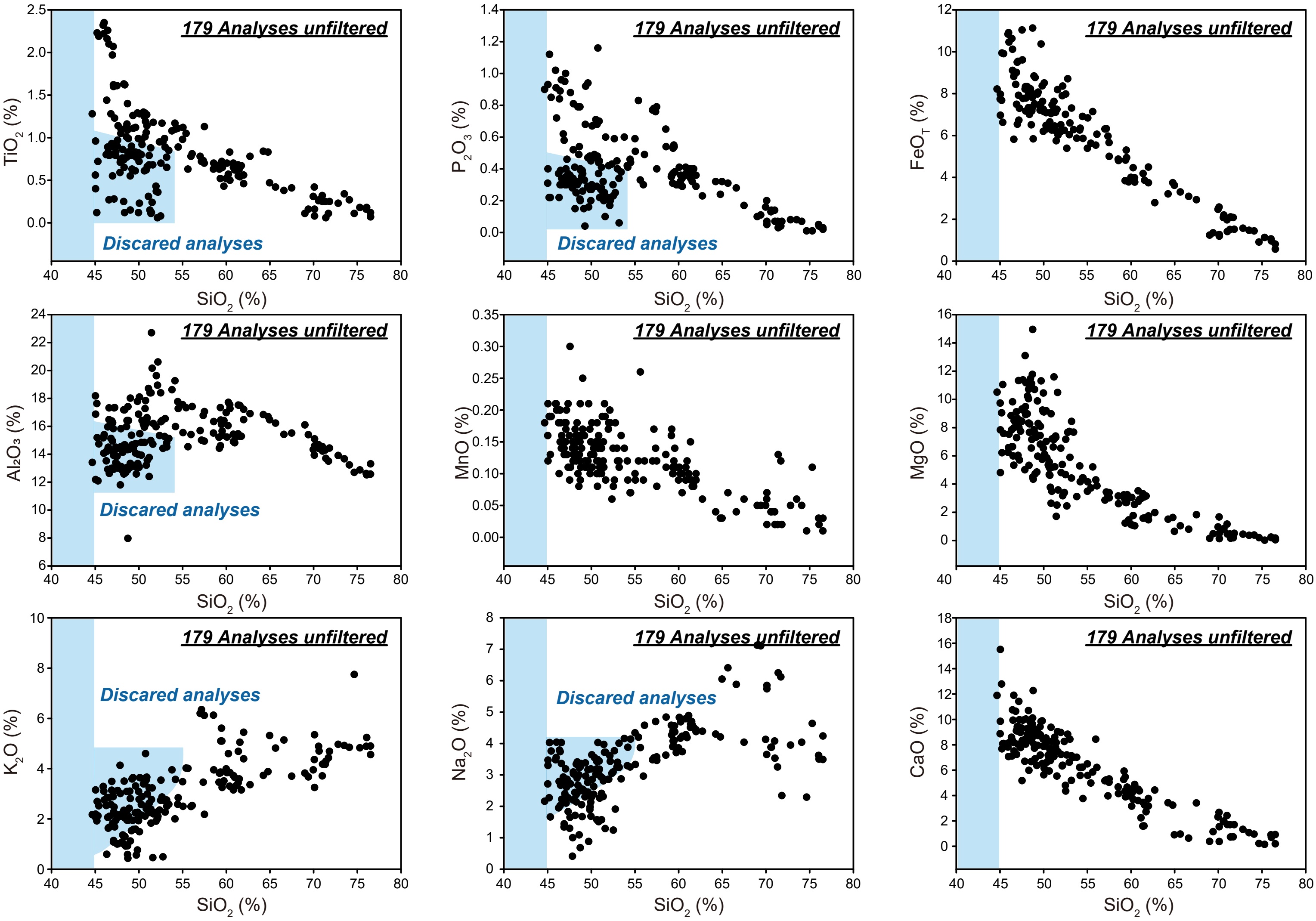

Jiaodong Peninsula 125-110 Ma Arc Segment GAME Results

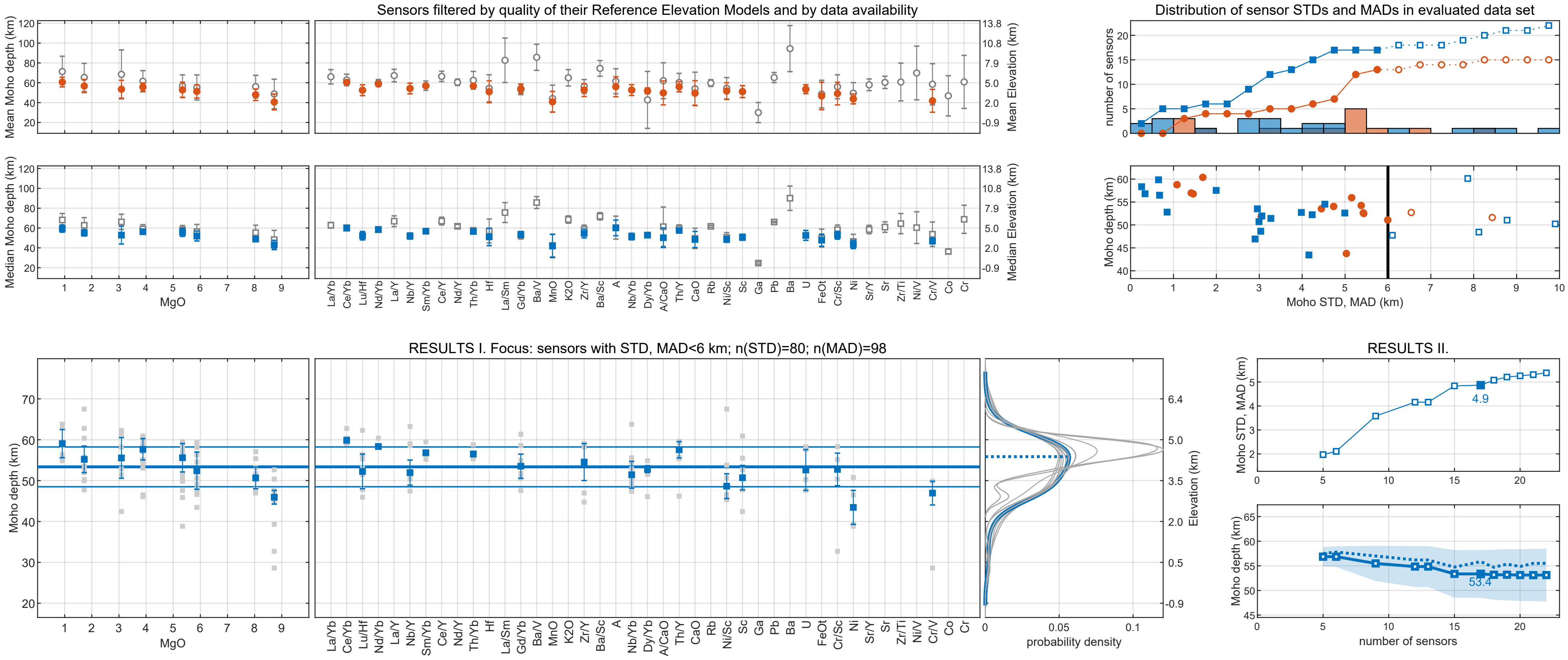

Figure S5-06 Korea Peninsula (KP): 192-179 Ma Magmatism

Individual Elevation vs. Age (192-165 Ma)

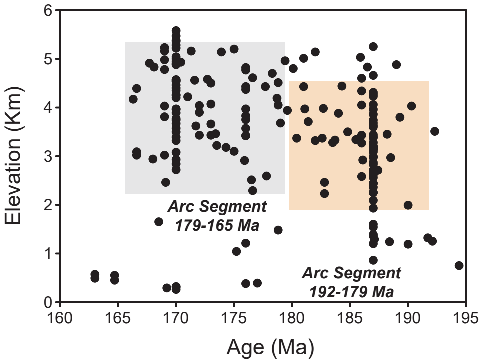

TAS diagram (192-179 Ma)

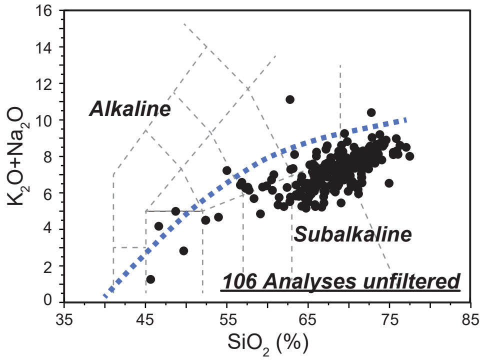

Individual Elevation vs. Longtitude and Latitude (192-179 Ma)

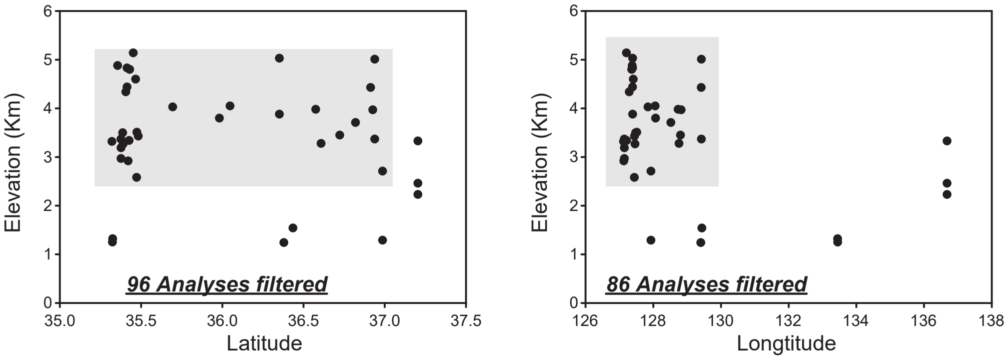

Harker Diagrams (192-179 Ma)

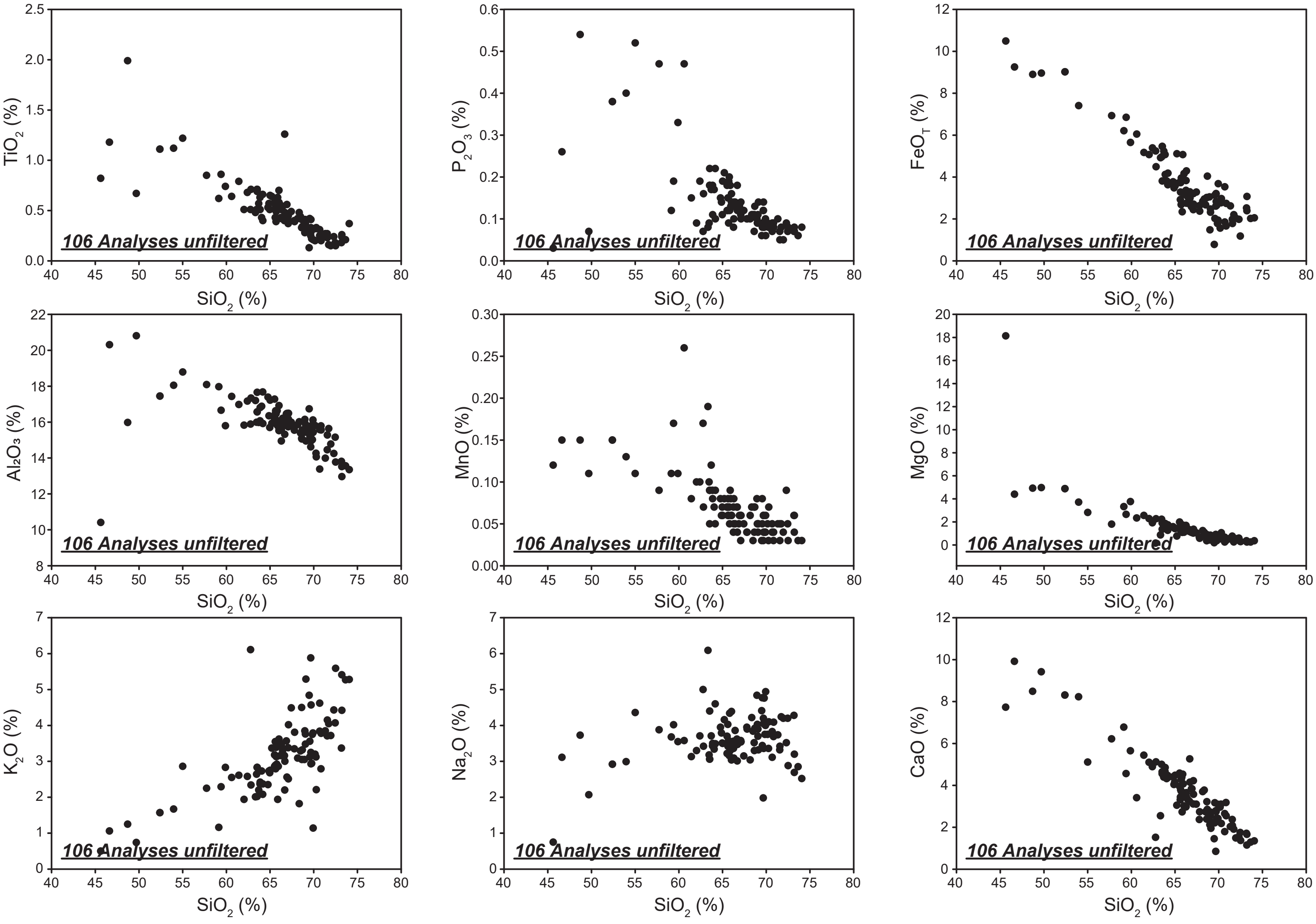

Korea Peninsula 192-179 Ma Arc Segment GAME Results

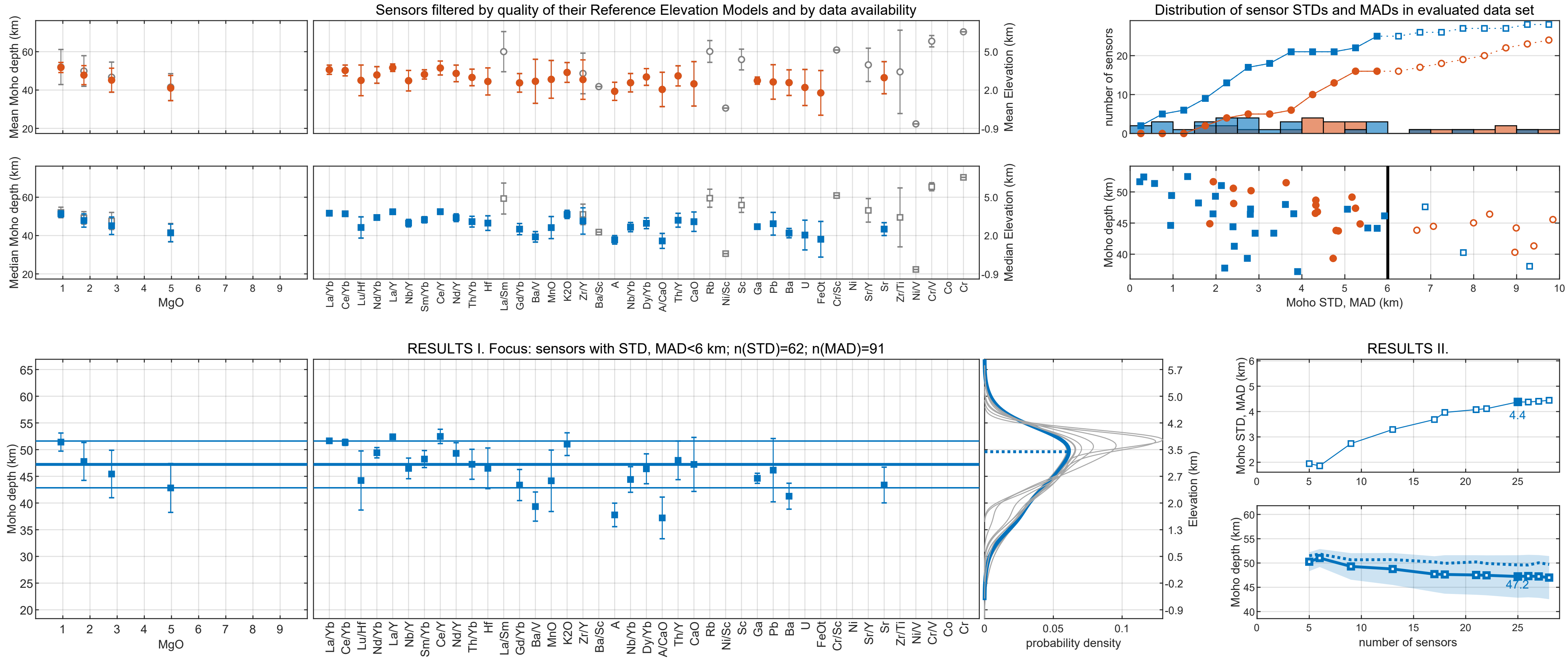

Figure S5-07

Korea Peninsula (KP): 179-165 Ma Magmatism

Individual Elevation vs. Age (192-165 Ma)

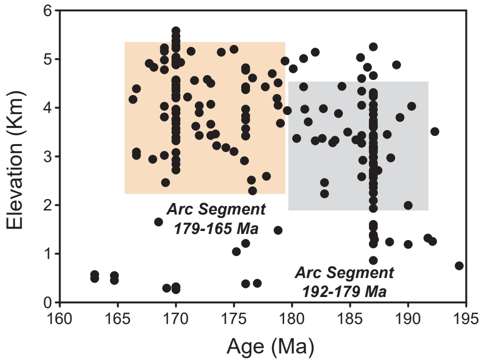

TAS diagram (179-165 Ma)

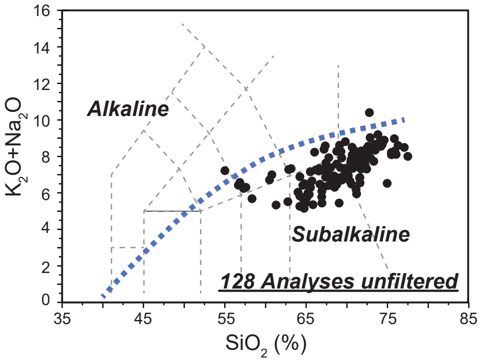

Individual Moho Depth vs. Longtitude and Latitude (179-165 Ma)

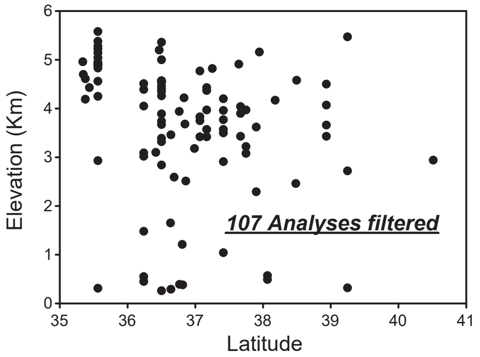

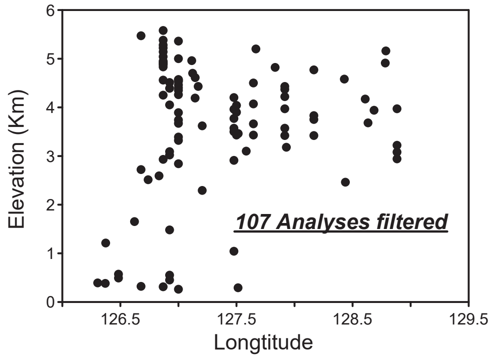

Harker Diagrams (179-165 Ma)

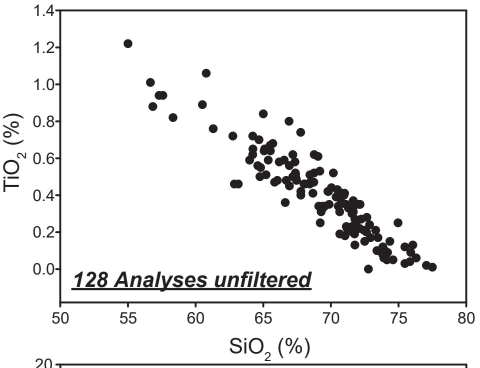

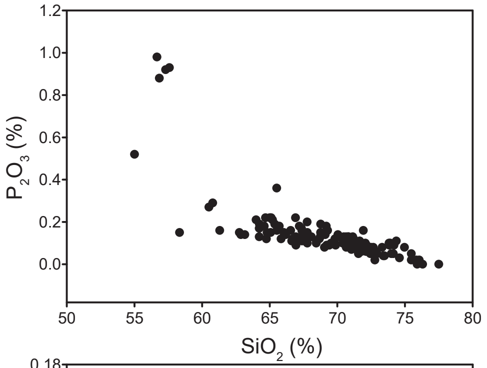

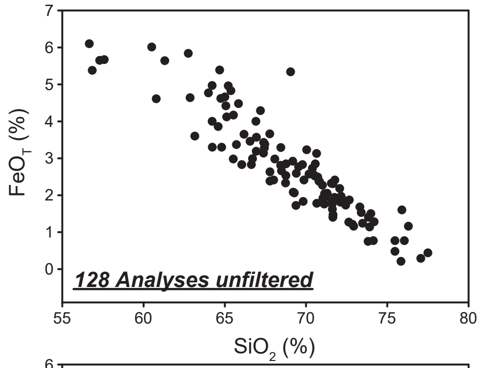

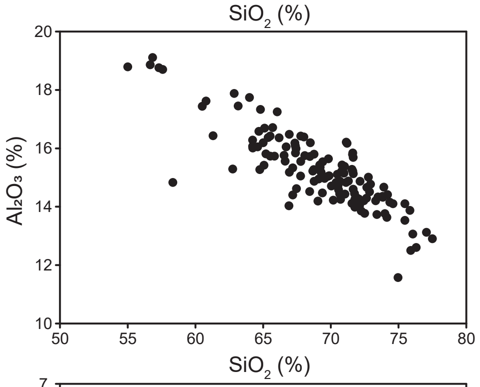

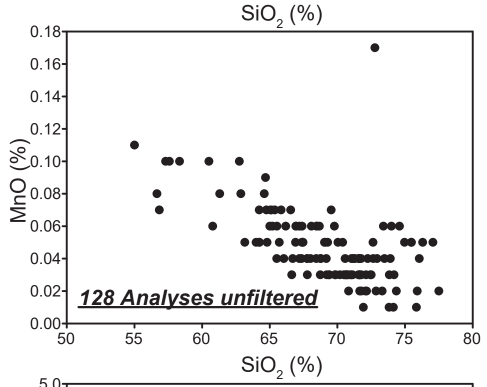

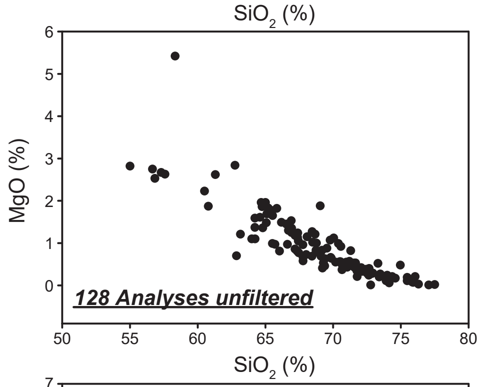

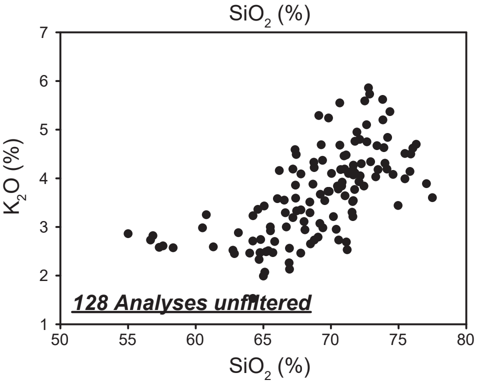

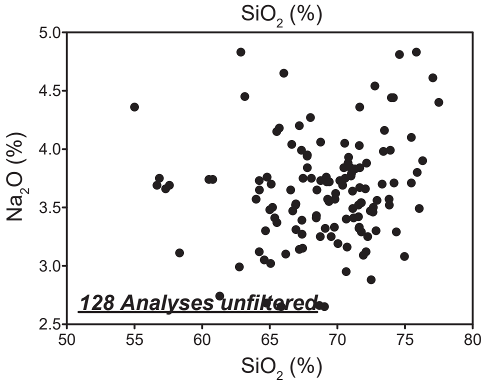

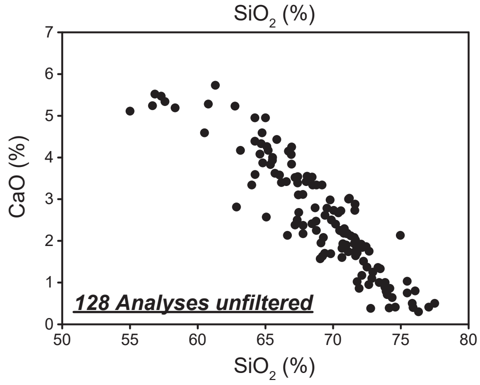

Korea Peninsula 179-165 Ma Arc Segment GAME Results

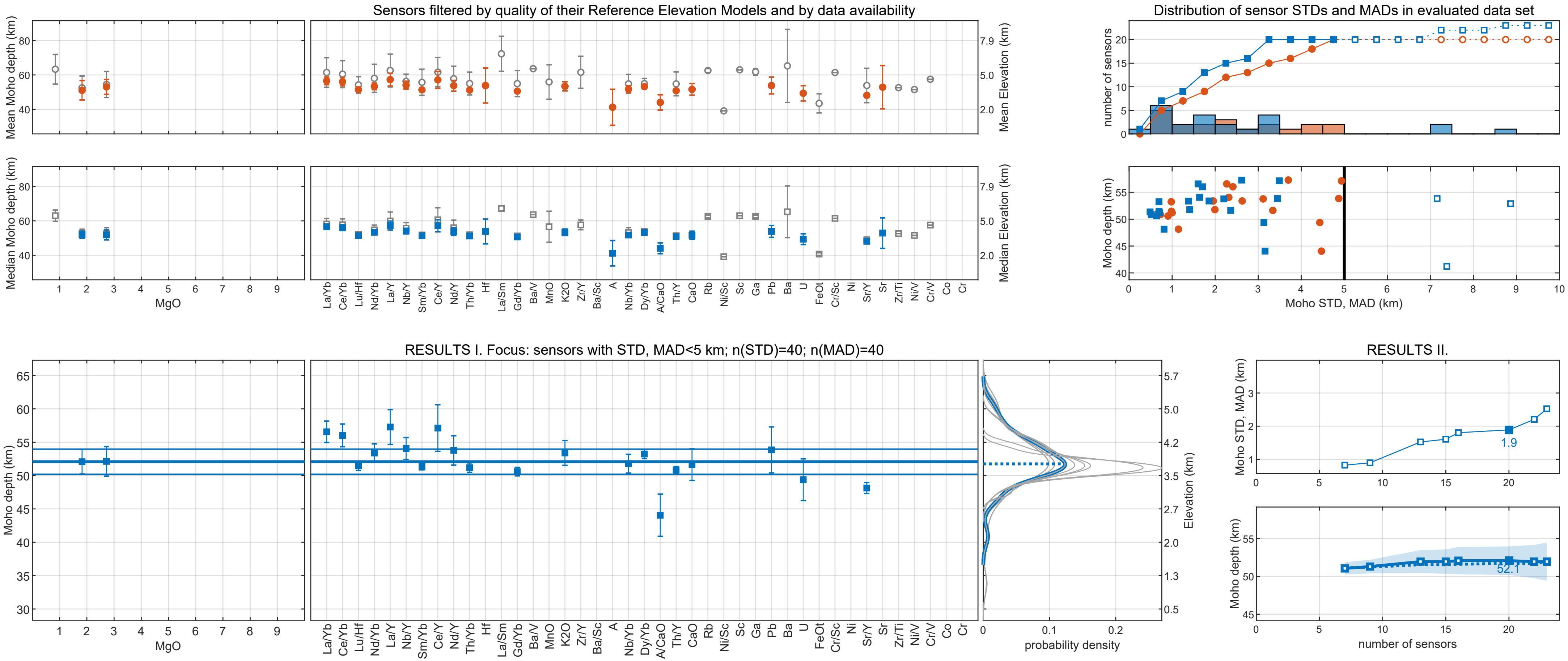

Figure S5-08 Korea Peninsula (KP): 112-109 Ma Magmatism

Individual Elevation vs. Age (112-66 Ma) and (112-109 Ma)

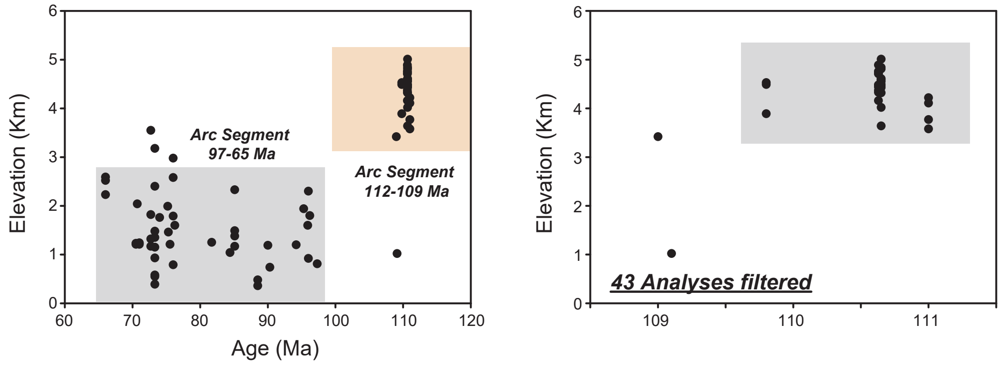

TAS diagram (112-90 Ma)

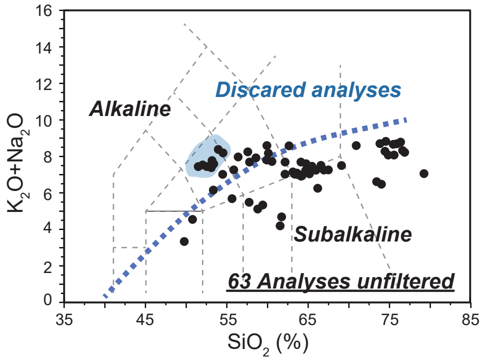

Individual Elevation vs. Longtitude and Latitude (112-109 Ma)

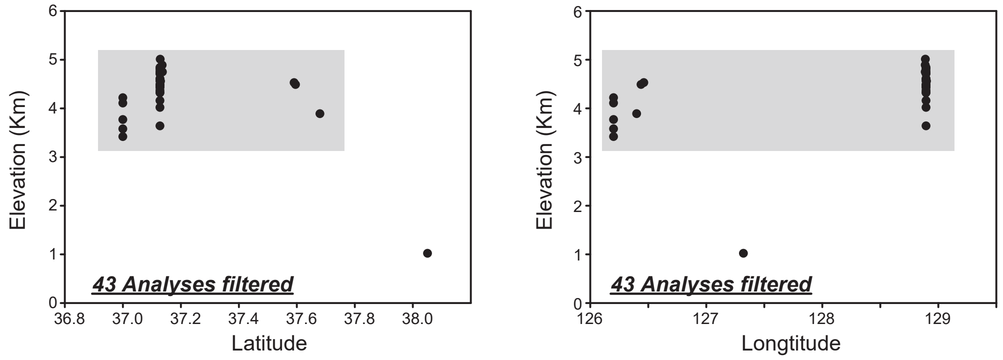

Harker Diagrams (112-90 Ma)

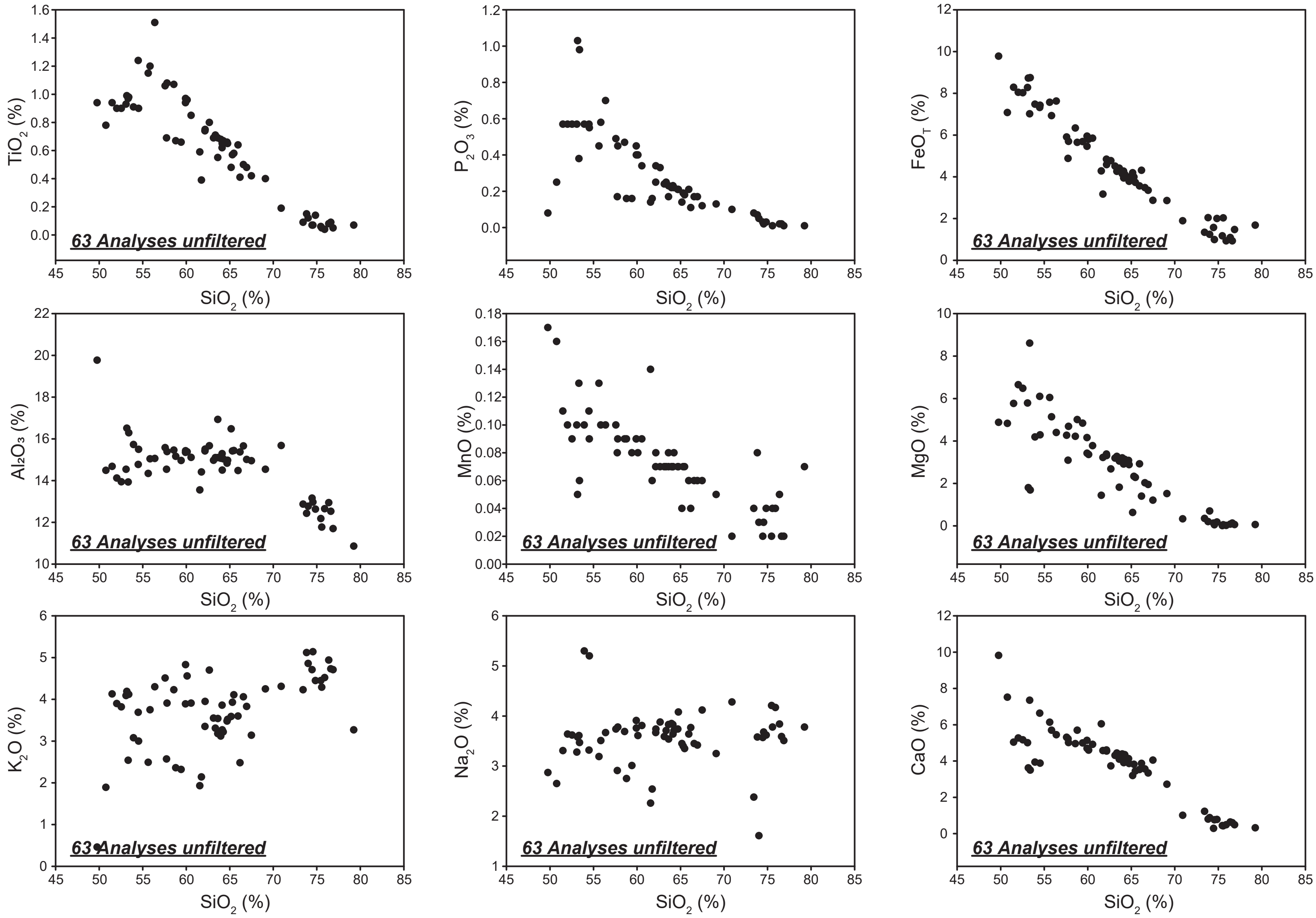

Korea Peninsula 112-90 Ma Arc Segment GAME Results

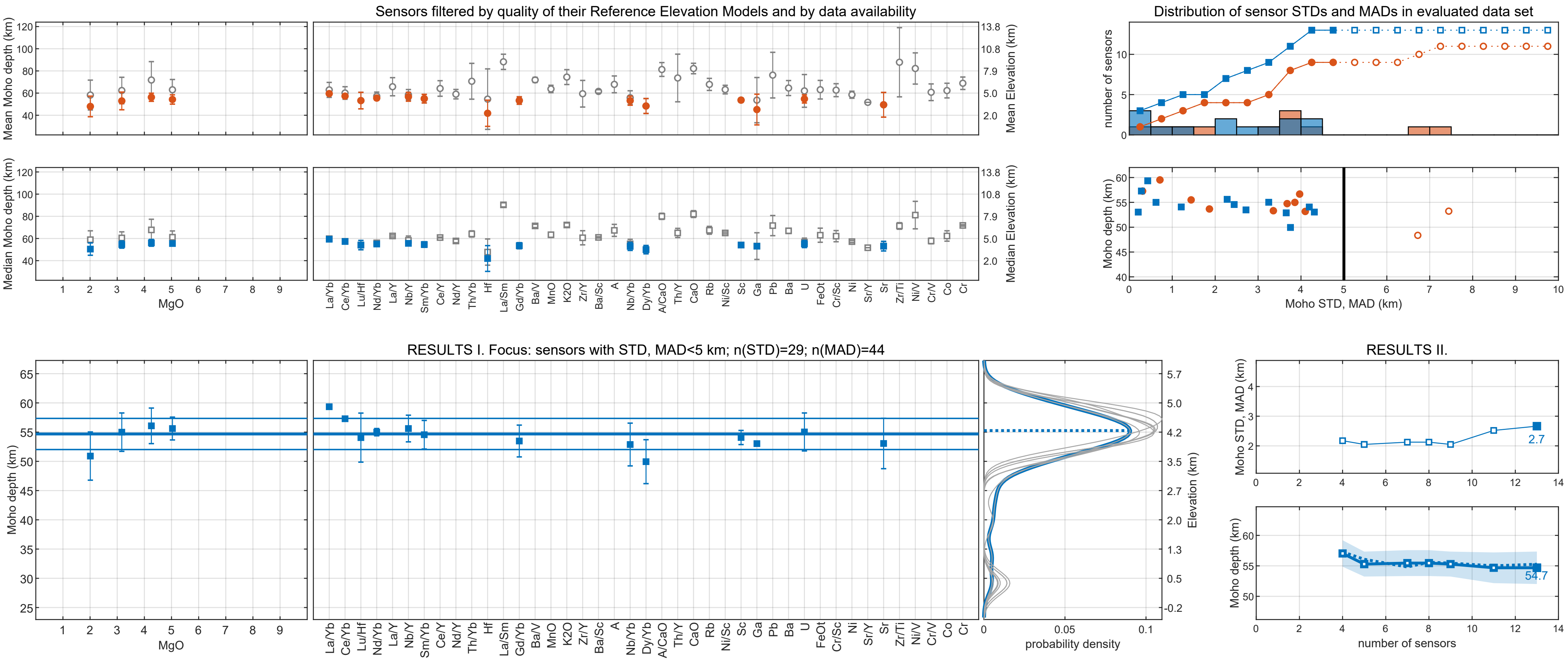

Figure S5-09 Liaodong Peninsula (LDP): 135-125 Ma Magmatism

Individual Elevation vs. Age (140-100 Ma)

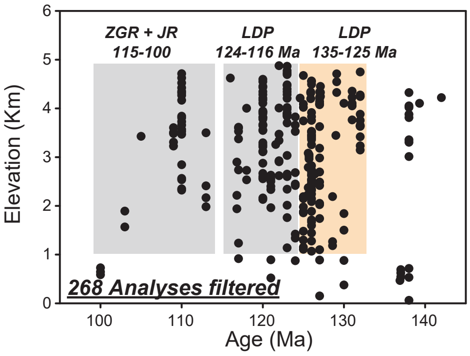

TAS diagram (135-125 Ma)

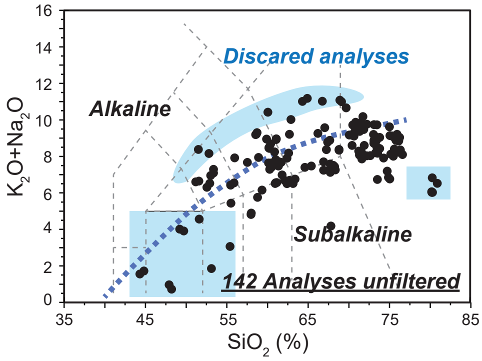

Individual Elevation vs. Latitude and Longtitude (140-100 Ma)

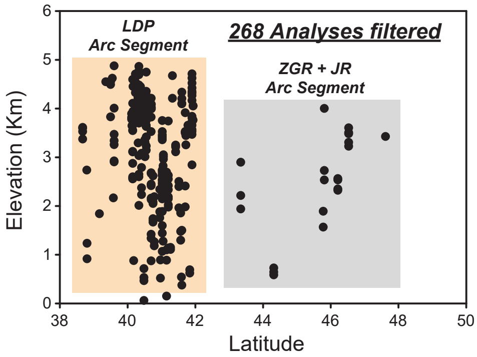

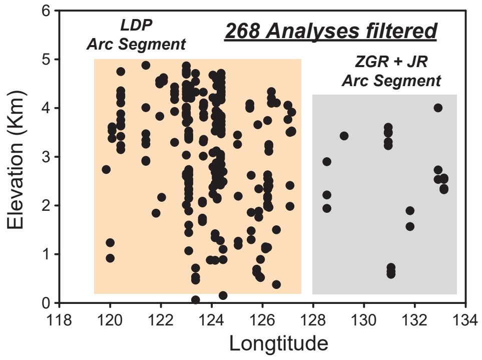

TAS diagram (135-125 Ma)

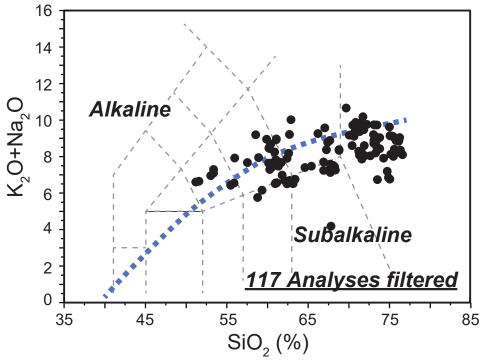

Harker Diagrams (135-125 Ma)

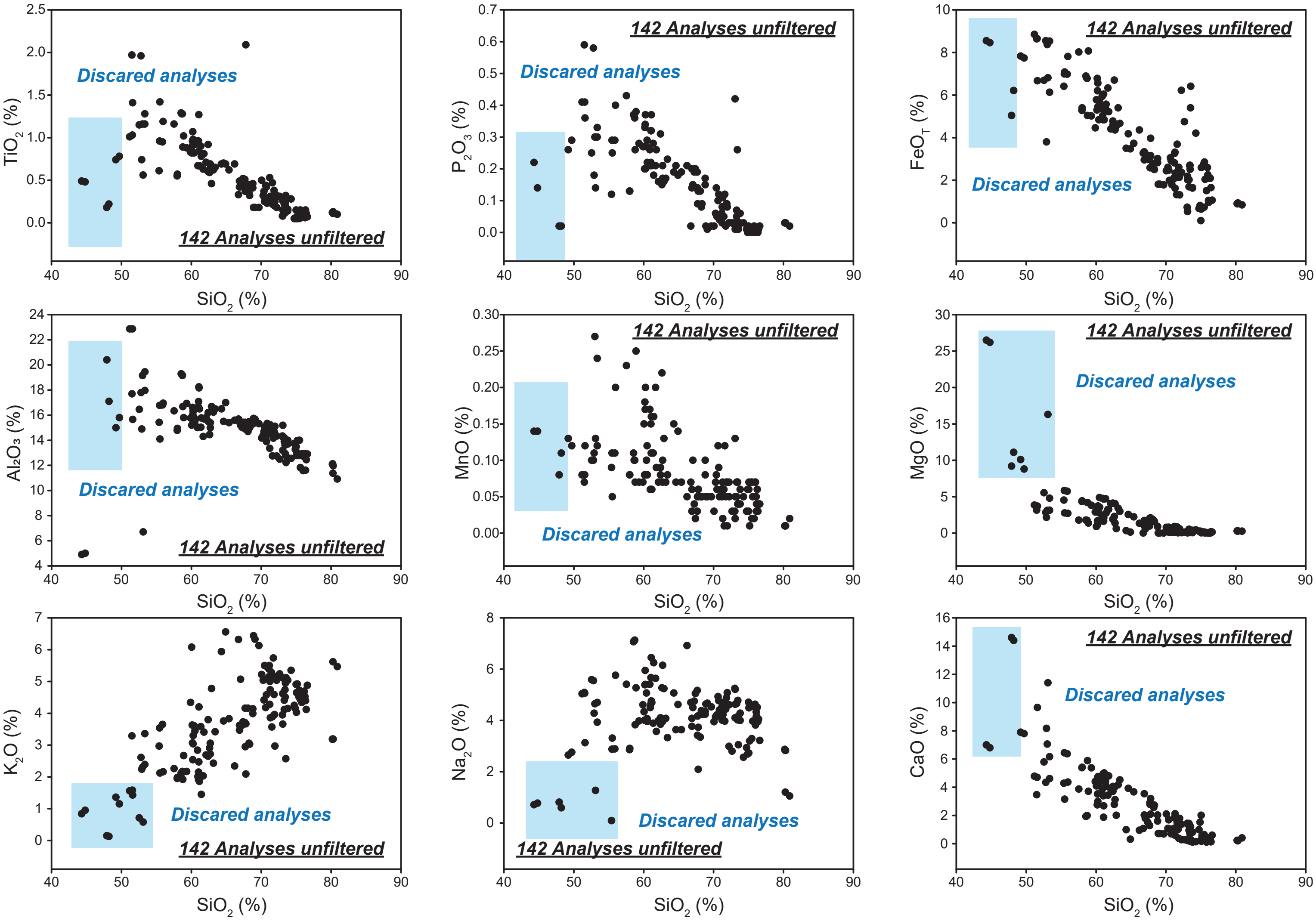

Liaodong Peninsula 135-125 Ma Arc Segment GAME Results

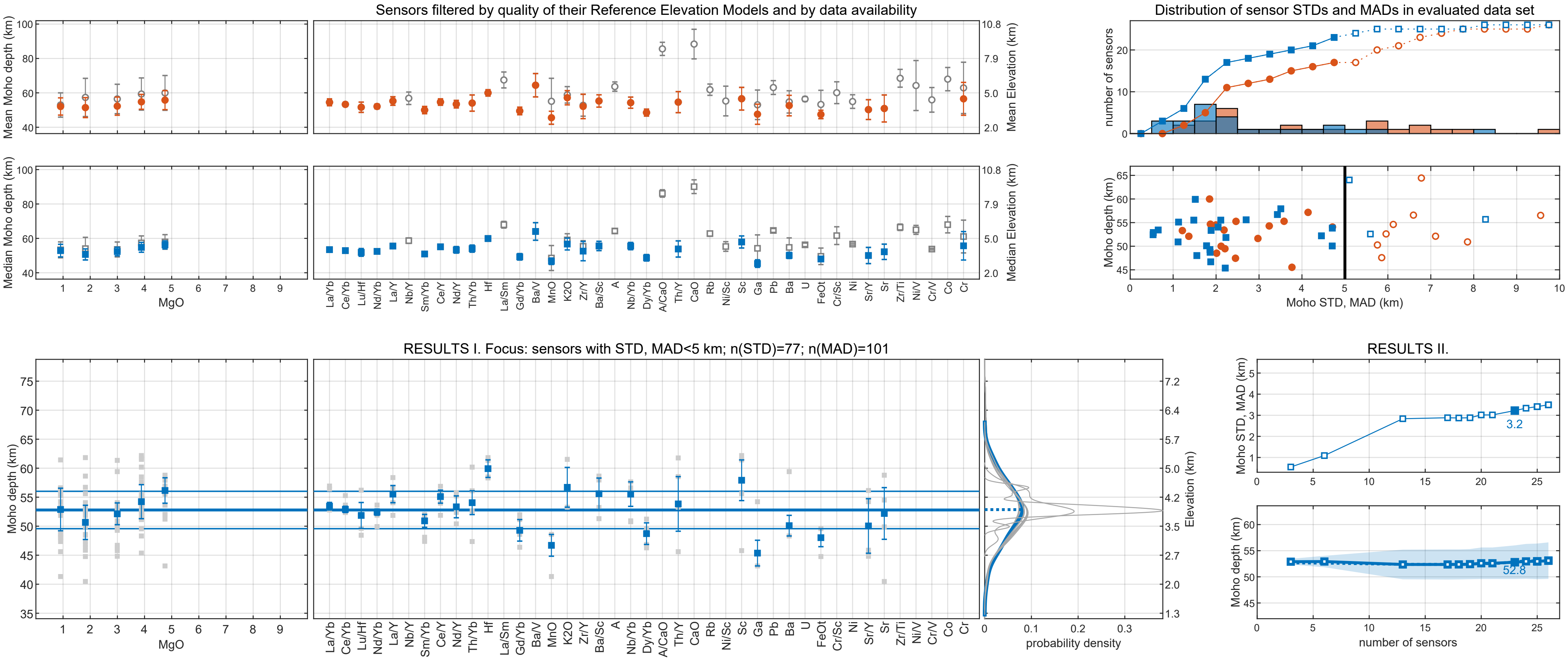

Figure S5-10 Liaodong Peninsula (LDP): 124-116 Ma Magmatism

Individual Elevation vs. Age (140-100 Ma)

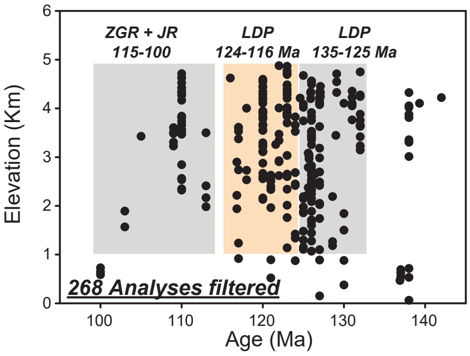

TAS diagram (124-116 Ma)

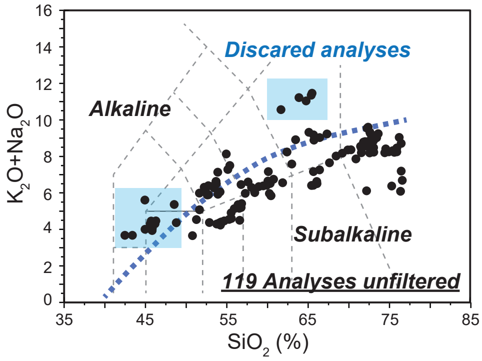

Individual Elevation vs. Latitude and Longtitude (140-100 Ma)

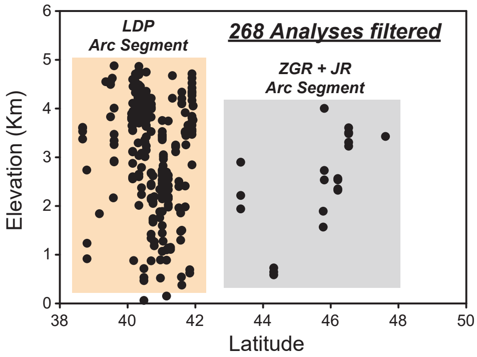

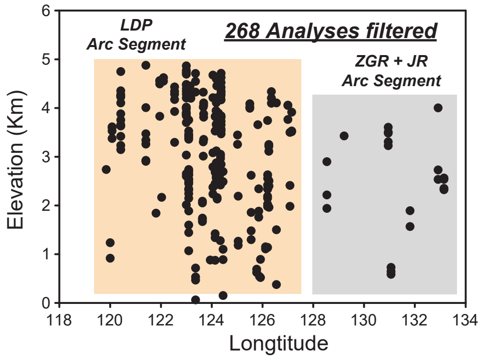

TAS diagram (124-116 Ma)

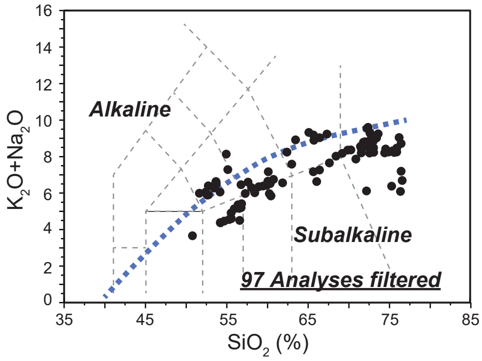

Harker Diagrams (124-116 Ma)

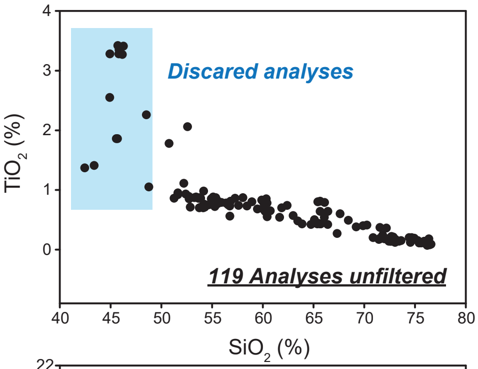

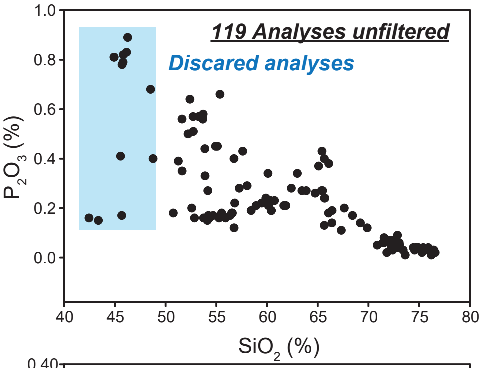

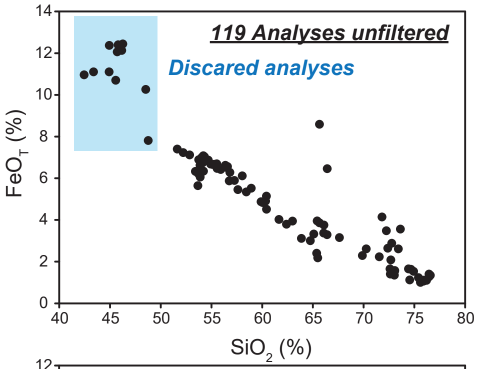

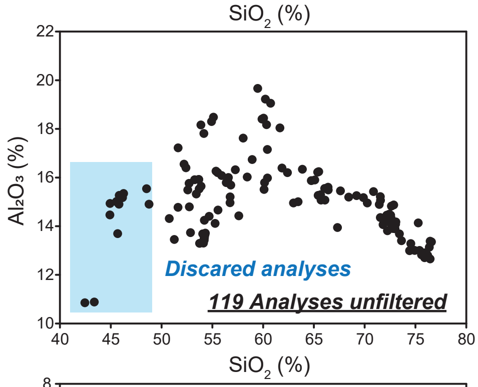

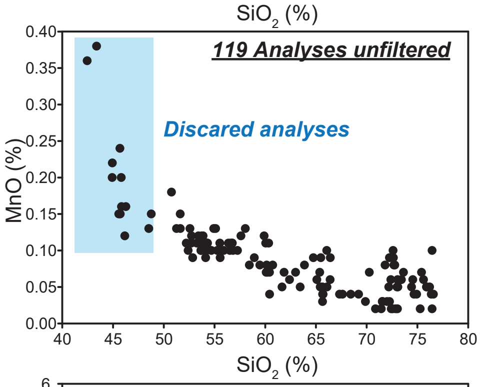

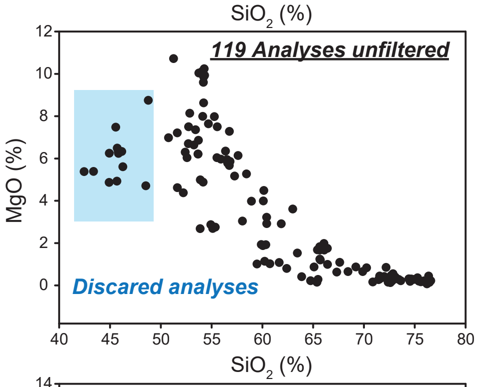

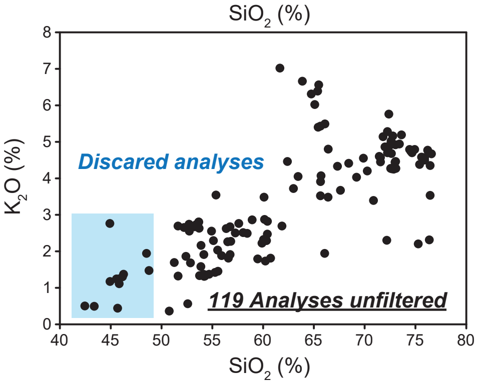

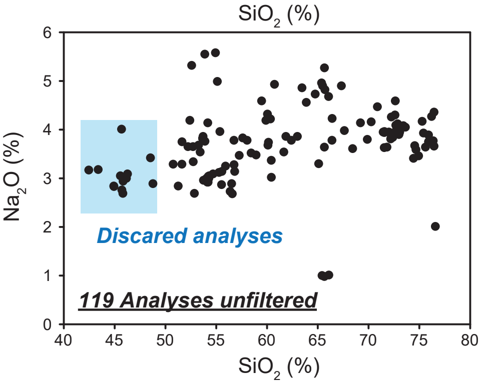

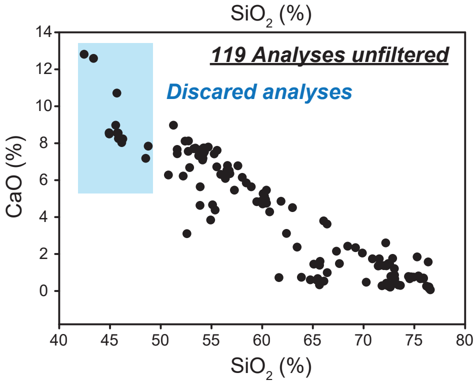

Liaodong Peninsula 124-116 Ma Arc Segment GAME Results

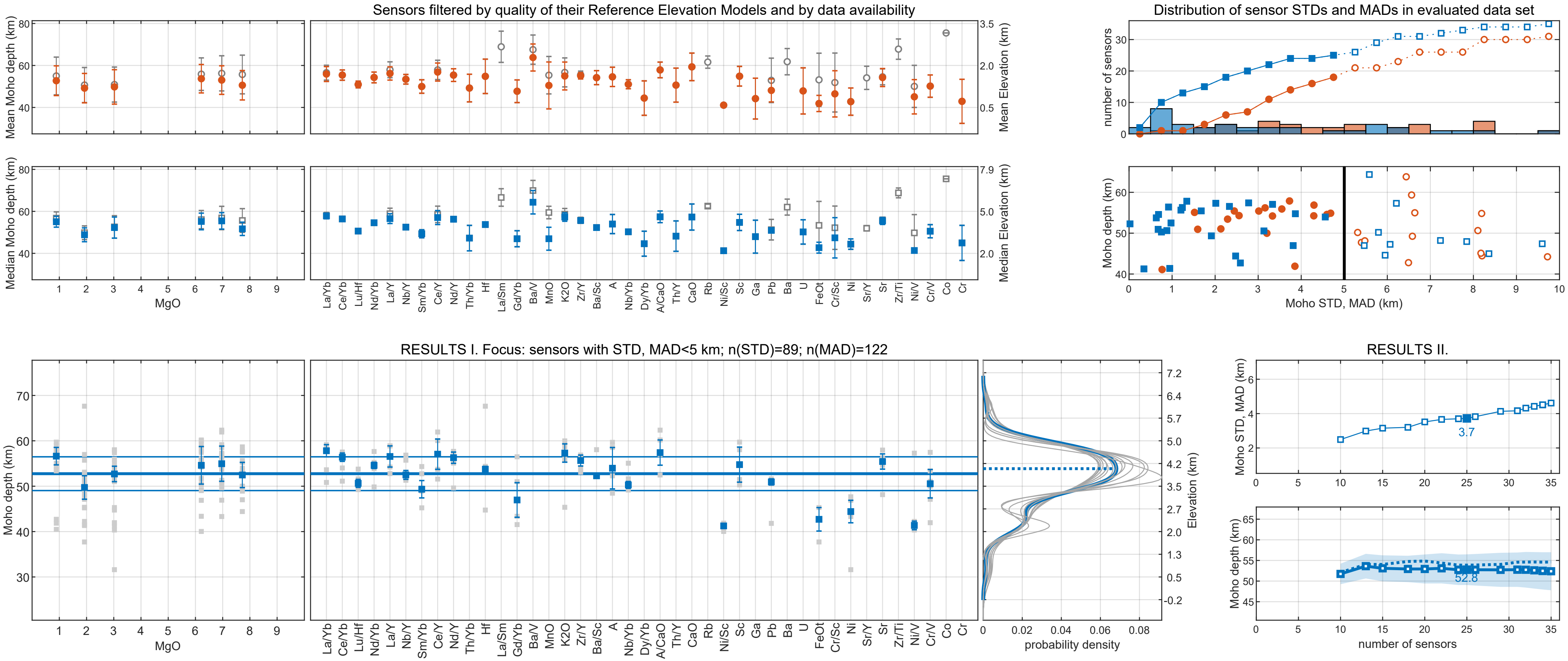

Figure S5-11

Yanshan Mountains (YSM): 173-160 Ma Magmatism

Individual Elevation vs. Age (173-113 Ma) and (173-160 Ma)

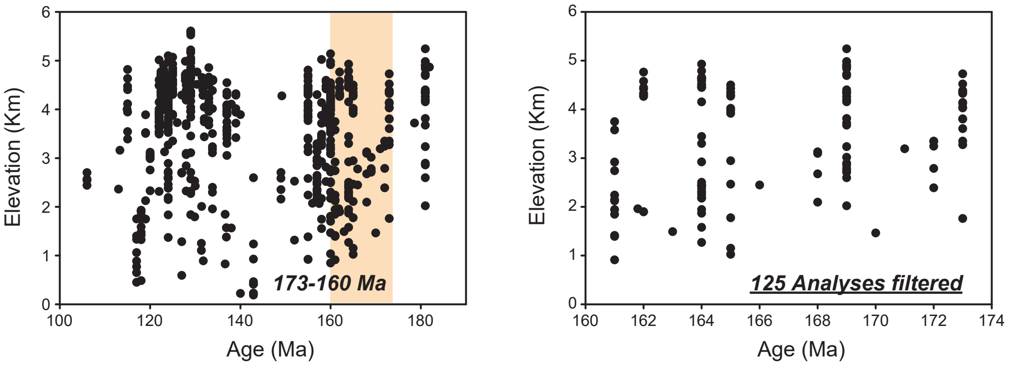

TAS diagram (173-160 Ma)

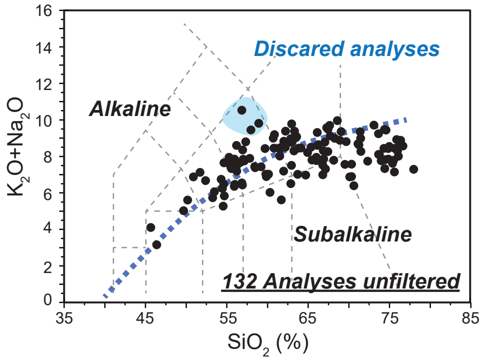

Individual Elevation vs. Longtitude and Latitude (173-160 Ma)

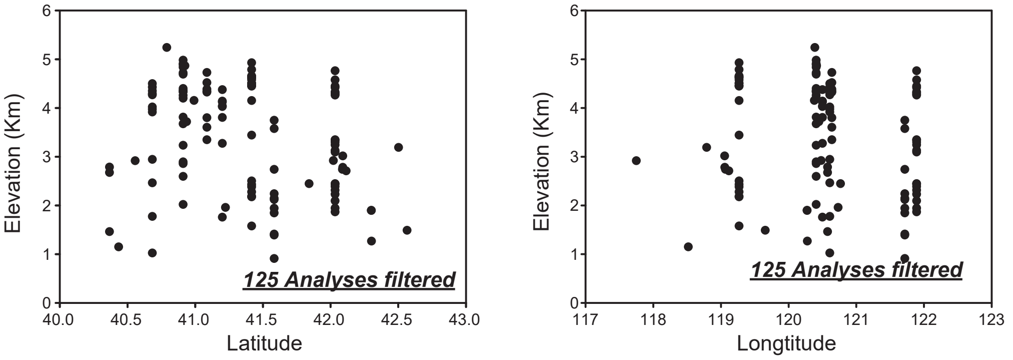

TAS diagram (173-160 Ma)

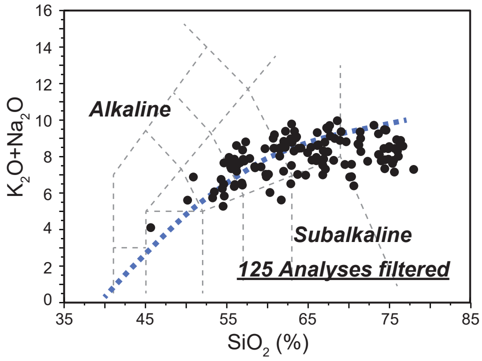

Harker Diagrams (173-160 Ma)

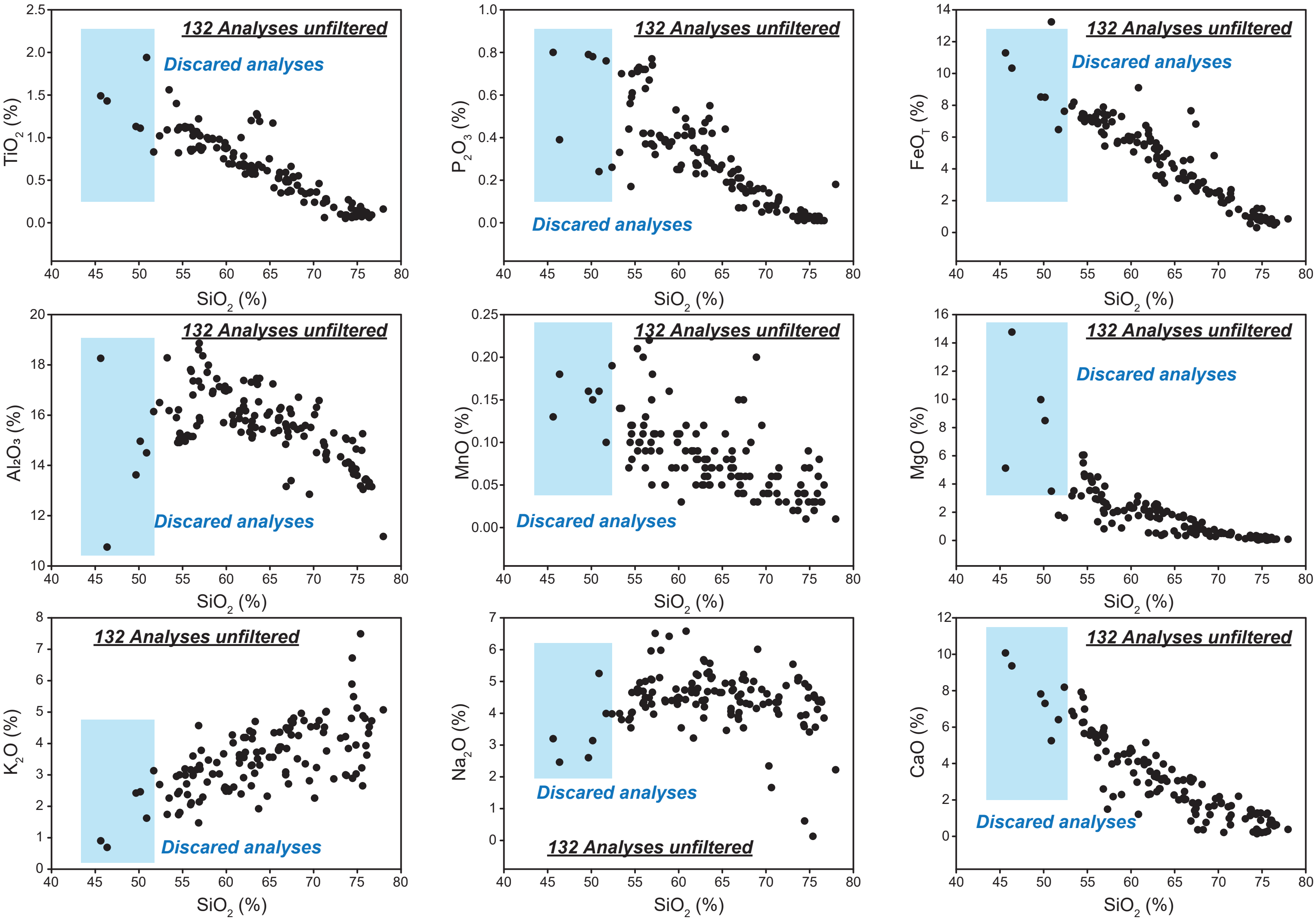

Yanshan Mountains 173-160 Ma Arc Segment GAME Results

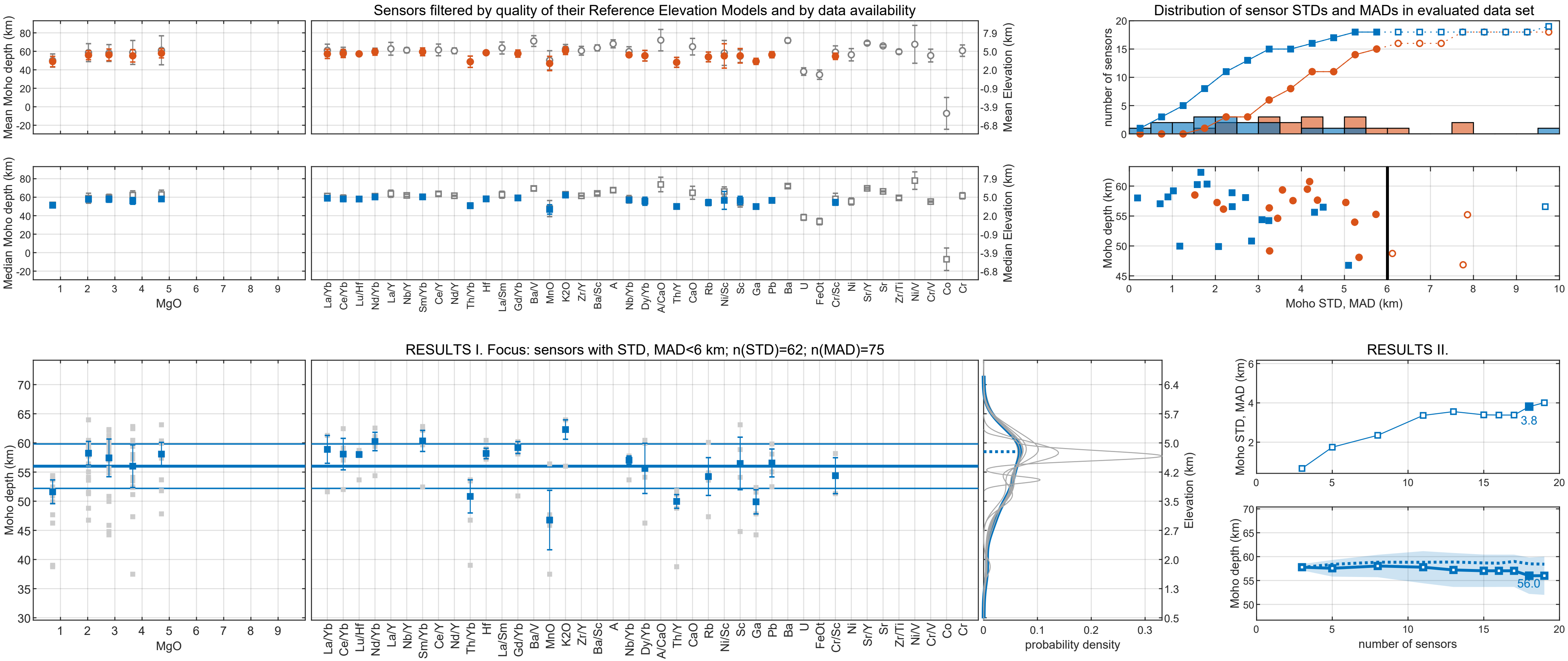

Figure S5-12 Yanshan Mountains (YSM): 160-150 Ma Magmatism

Individual Elevation vs. Age (182-113 Ma) and (160-150 Ma)

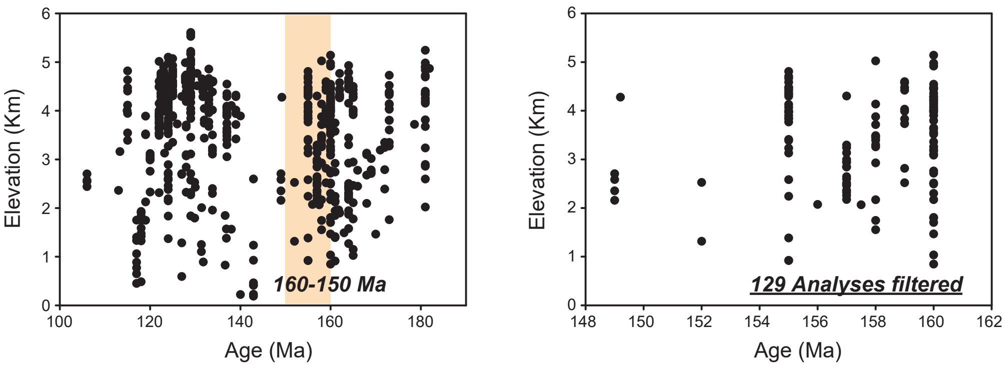

TAS diagram (160-150 Ma)

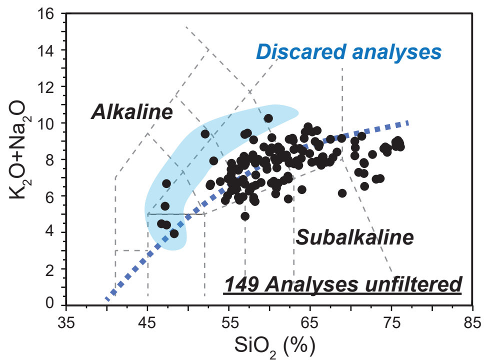

Individual Elevation vs. Longtitude and Latitude (160-150 Ma)

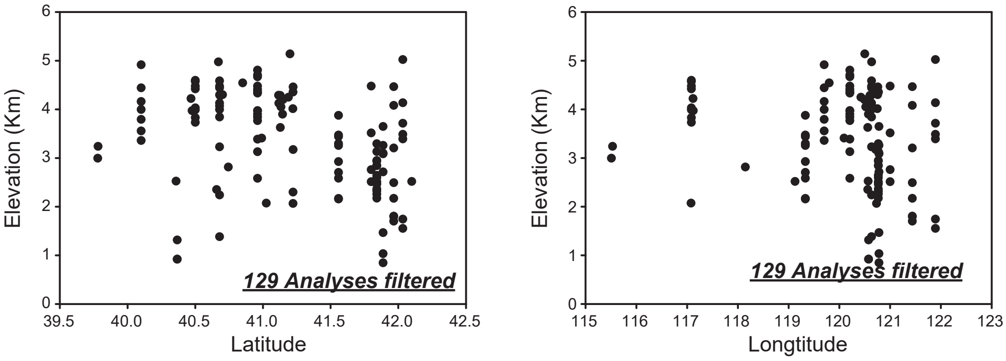

TAS diagram (160-150 Ma)

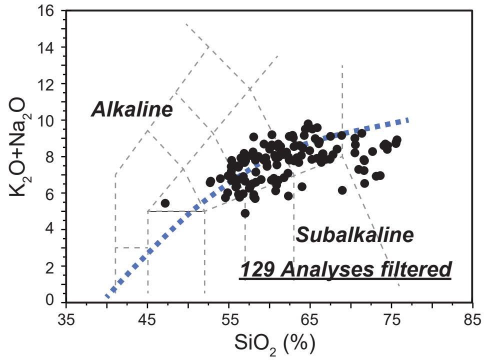

Harker Diagrams (160-150 Ma)

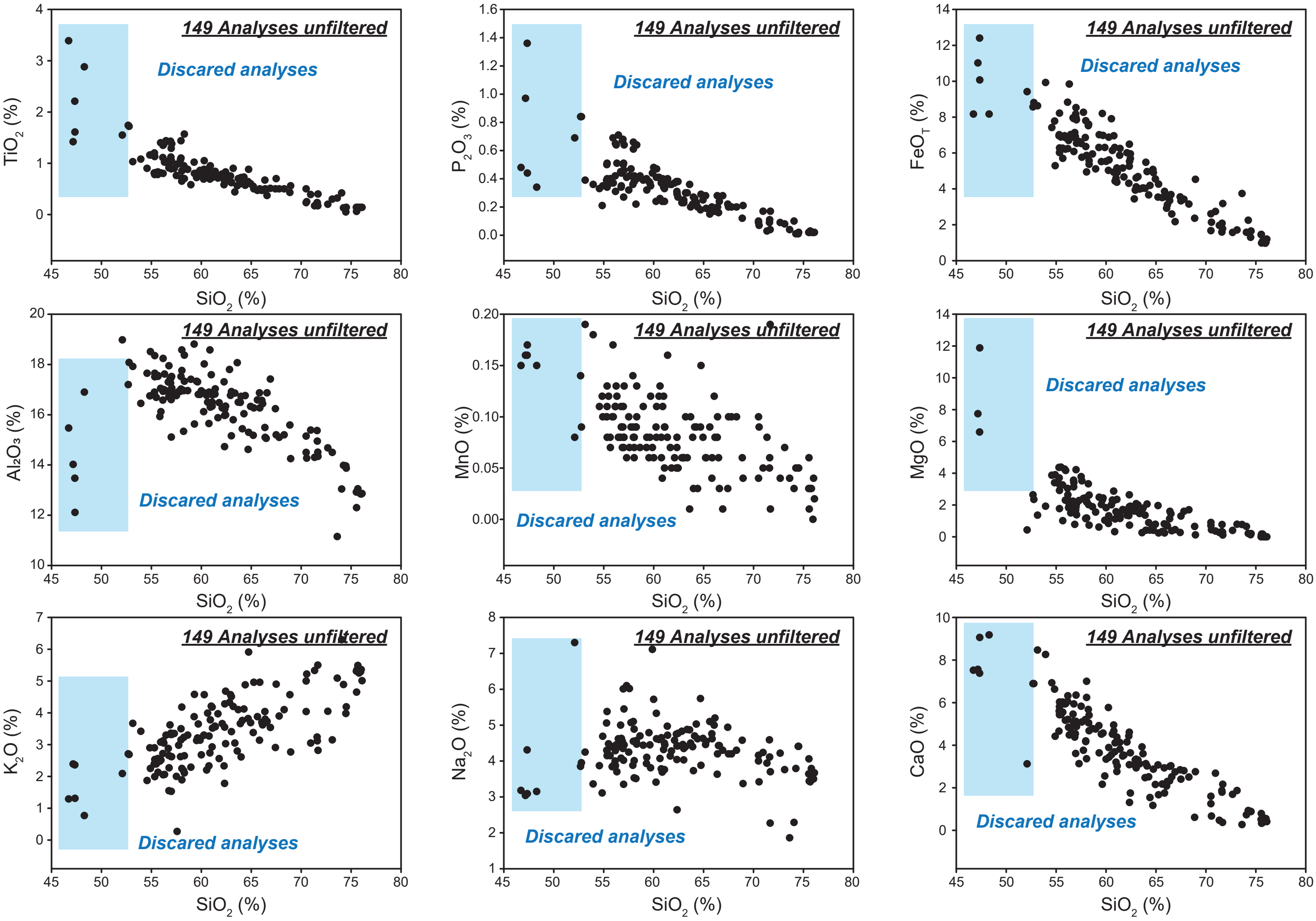

Yanshan Mountains 160-150 Ma Arc Segment GAME Results

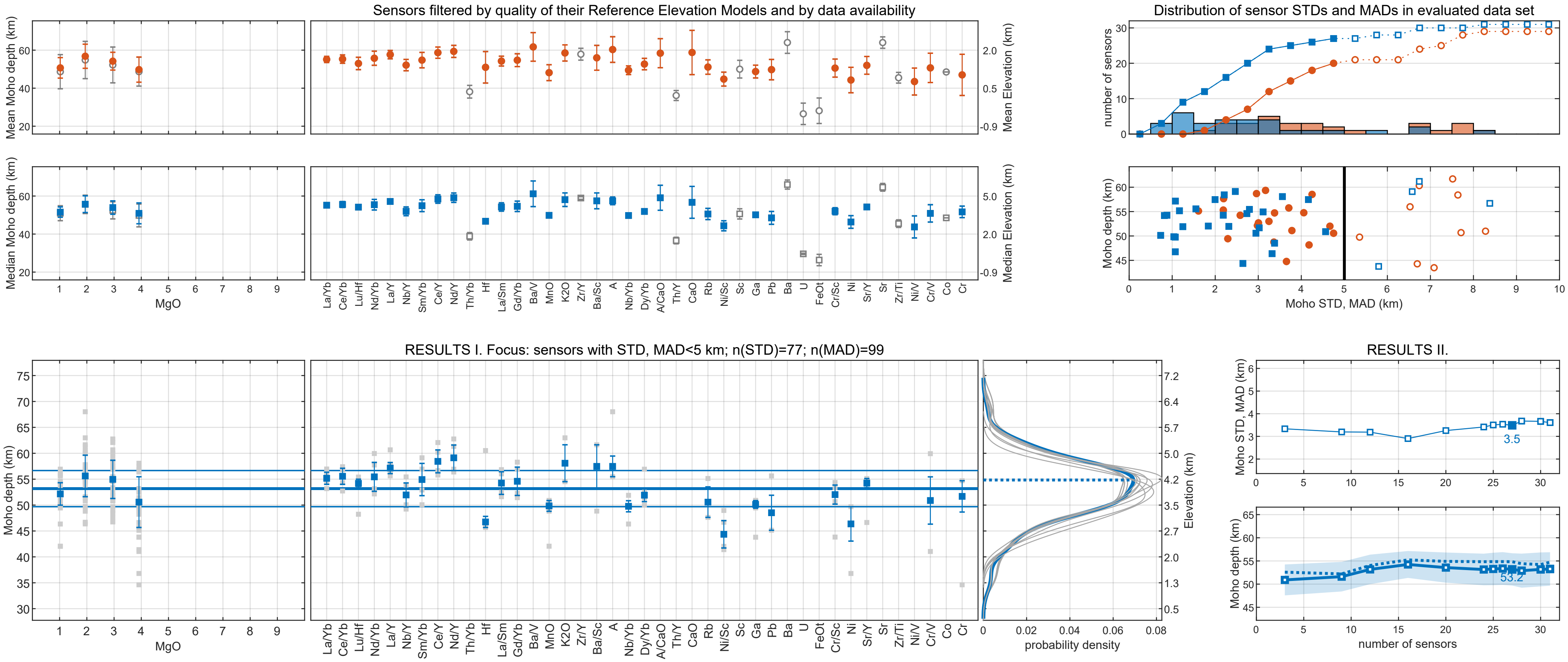

Figure S5-13 Yanshan Mountains (YSM): 140-125 Ma Magmatism

Individual Elevation vs. Age (182-113 Ma) and (140-125 Ma)

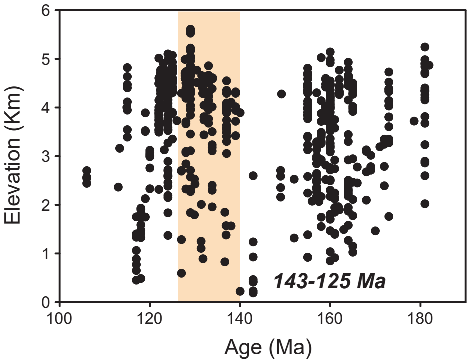

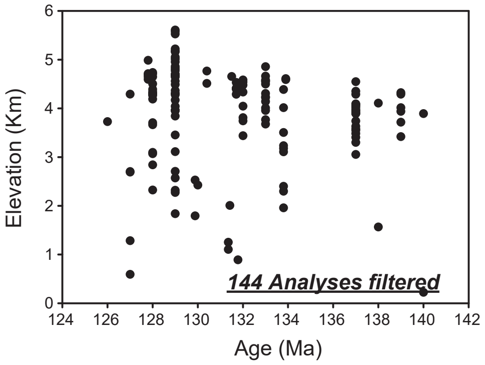

TAS diagram (140-125 Ma)

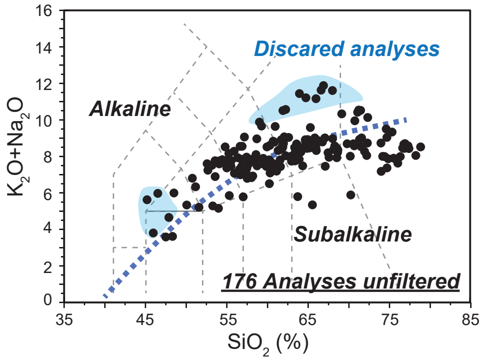

Individual Elevation vs. Longtitude and Latitude (140-125 Ma)

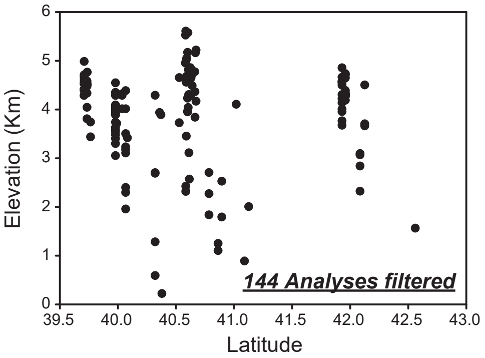

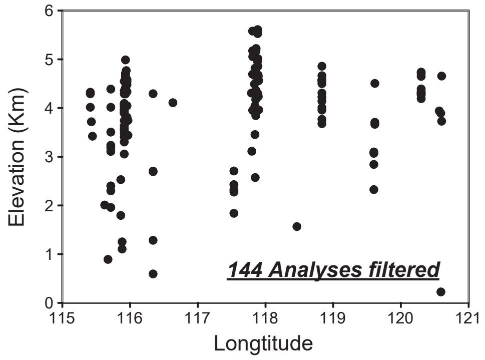

TAS diagram (140-125 Ma)

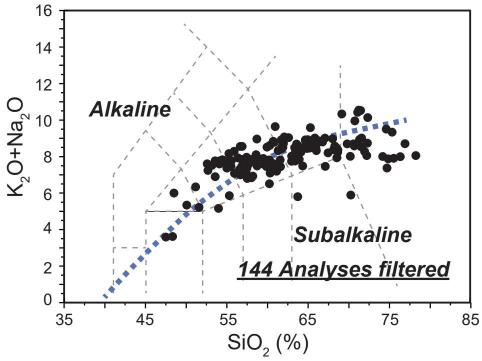

Harker Diagrams (143-125 Ma)

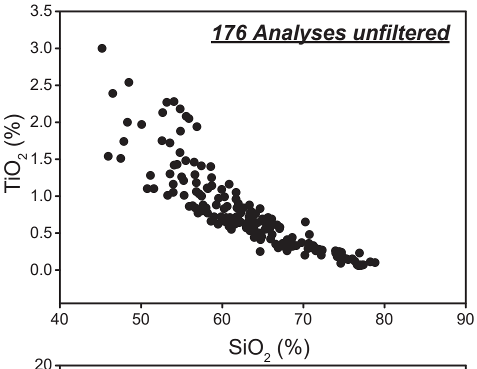

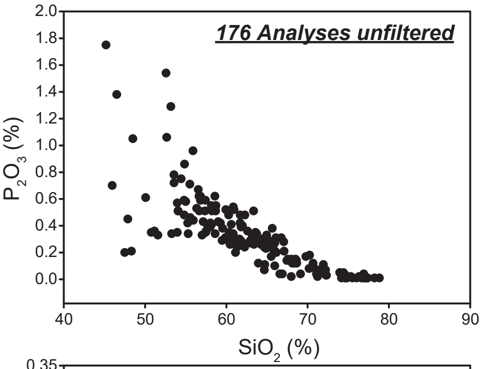

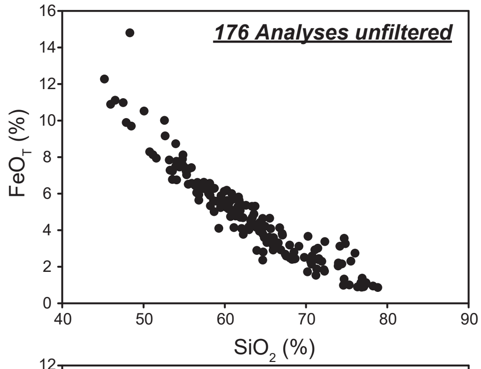

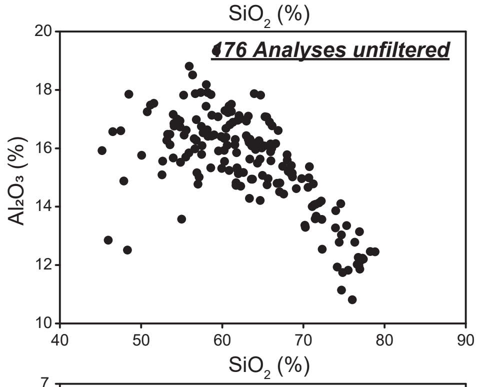

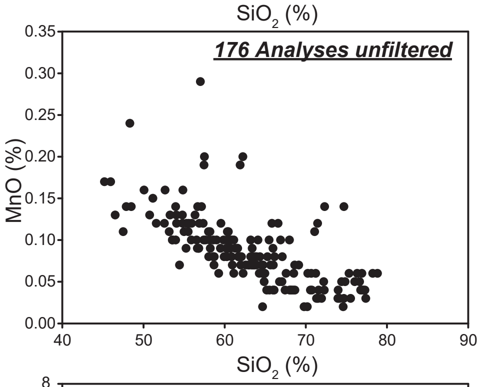

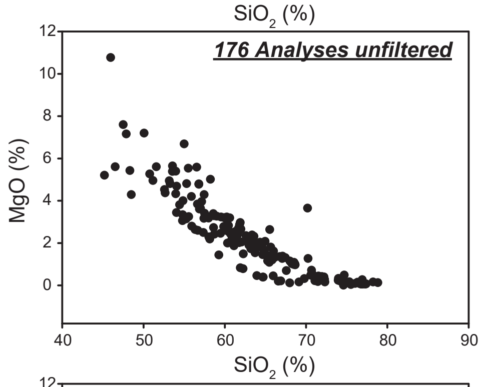

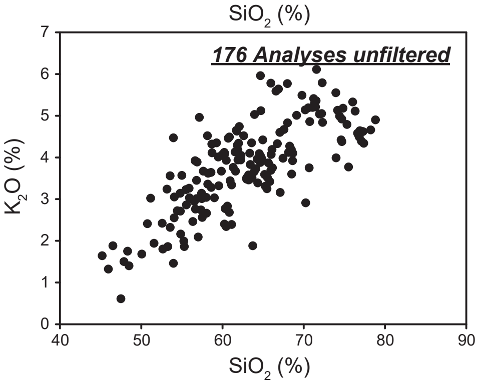

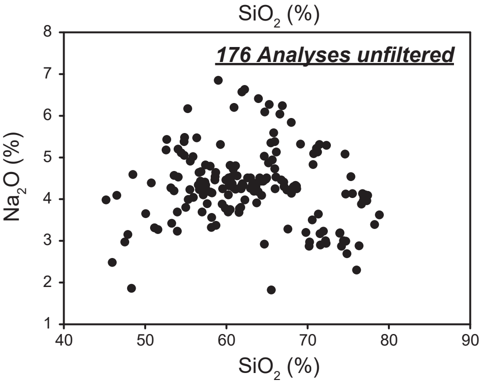

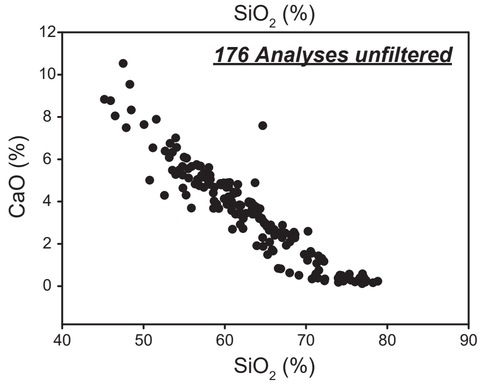

Yanshan Mountains 140-125 Ma Arc Segment GAME Results

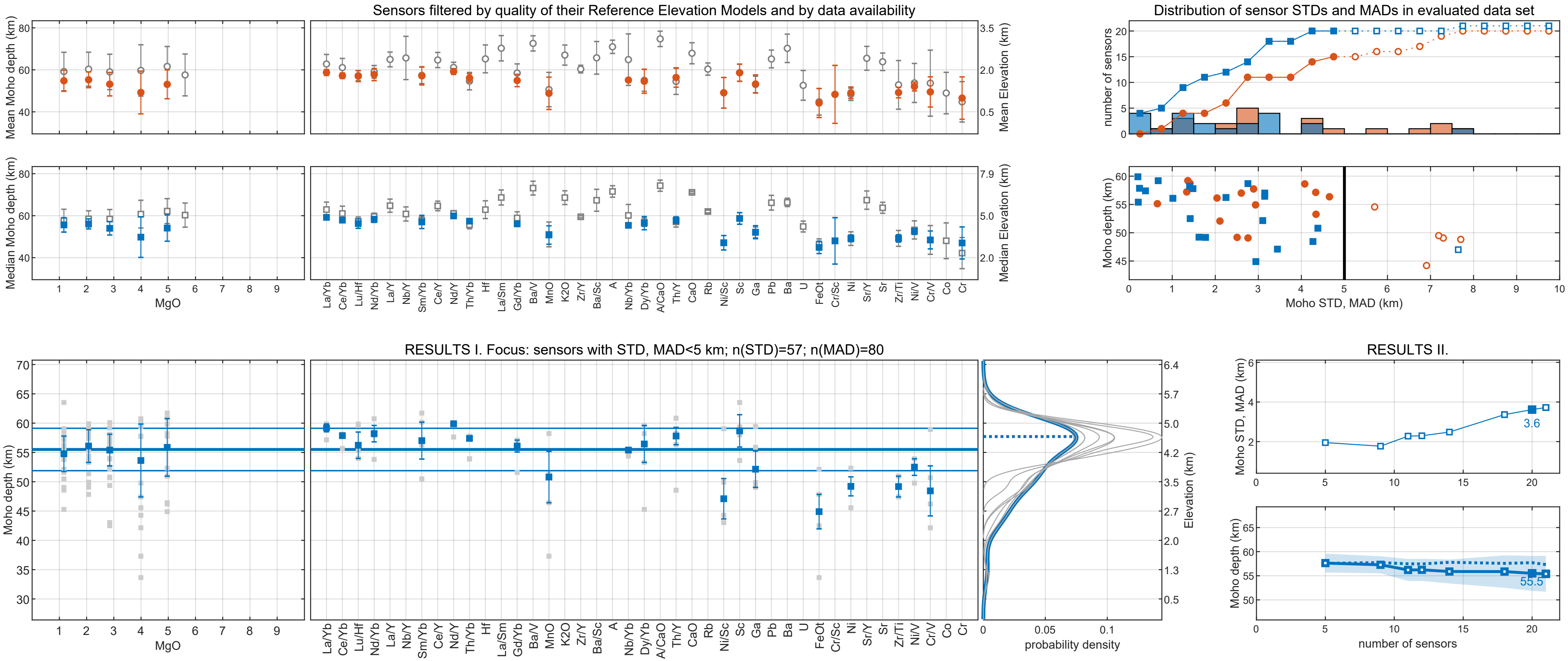

Figure S5-14 Yanshan Mountains (YSM): 125-122 Ma Magmatism

Individual Elevation vs. Age (182-106 Ma) and (125-106 Ma)

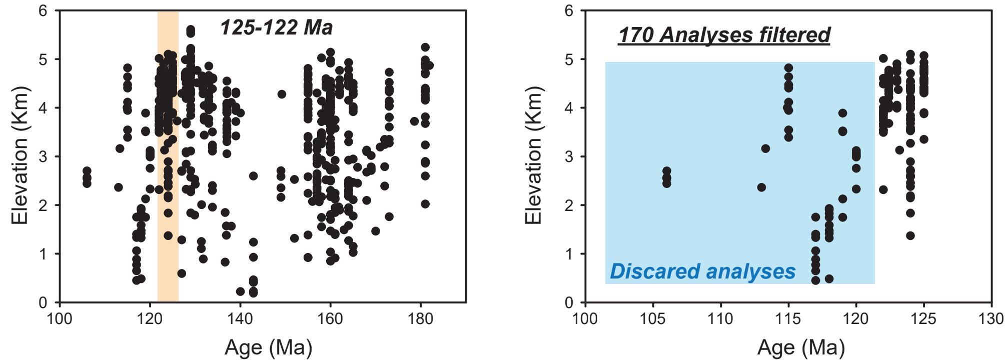

TAS diagram (125-113 Ma)

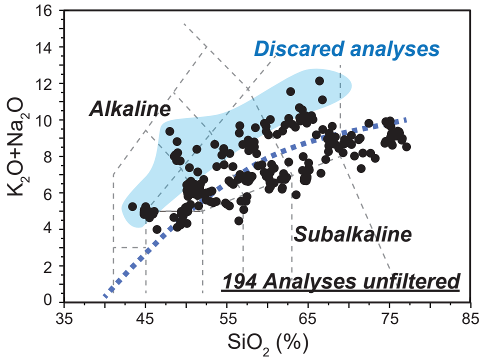

Individual Elevation vs. Longtitude and Latitude (125-106 Ma)

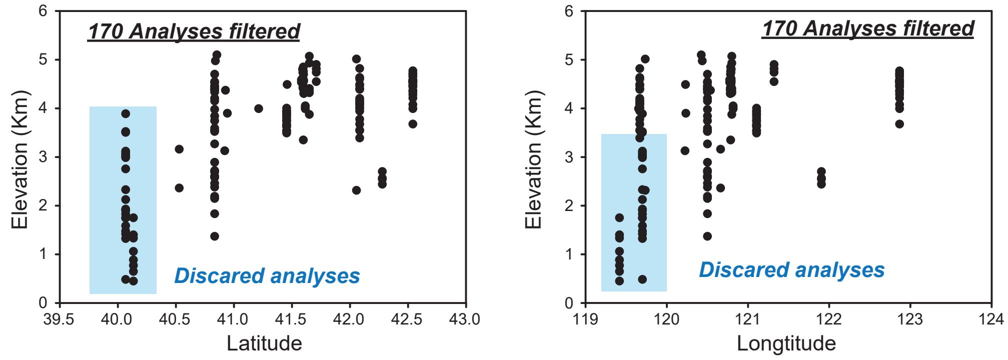

TAS diagram (125-122 Ma)

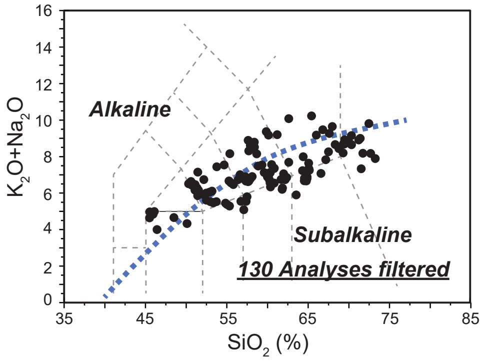

Harker Diagrams (125-113 Ma)

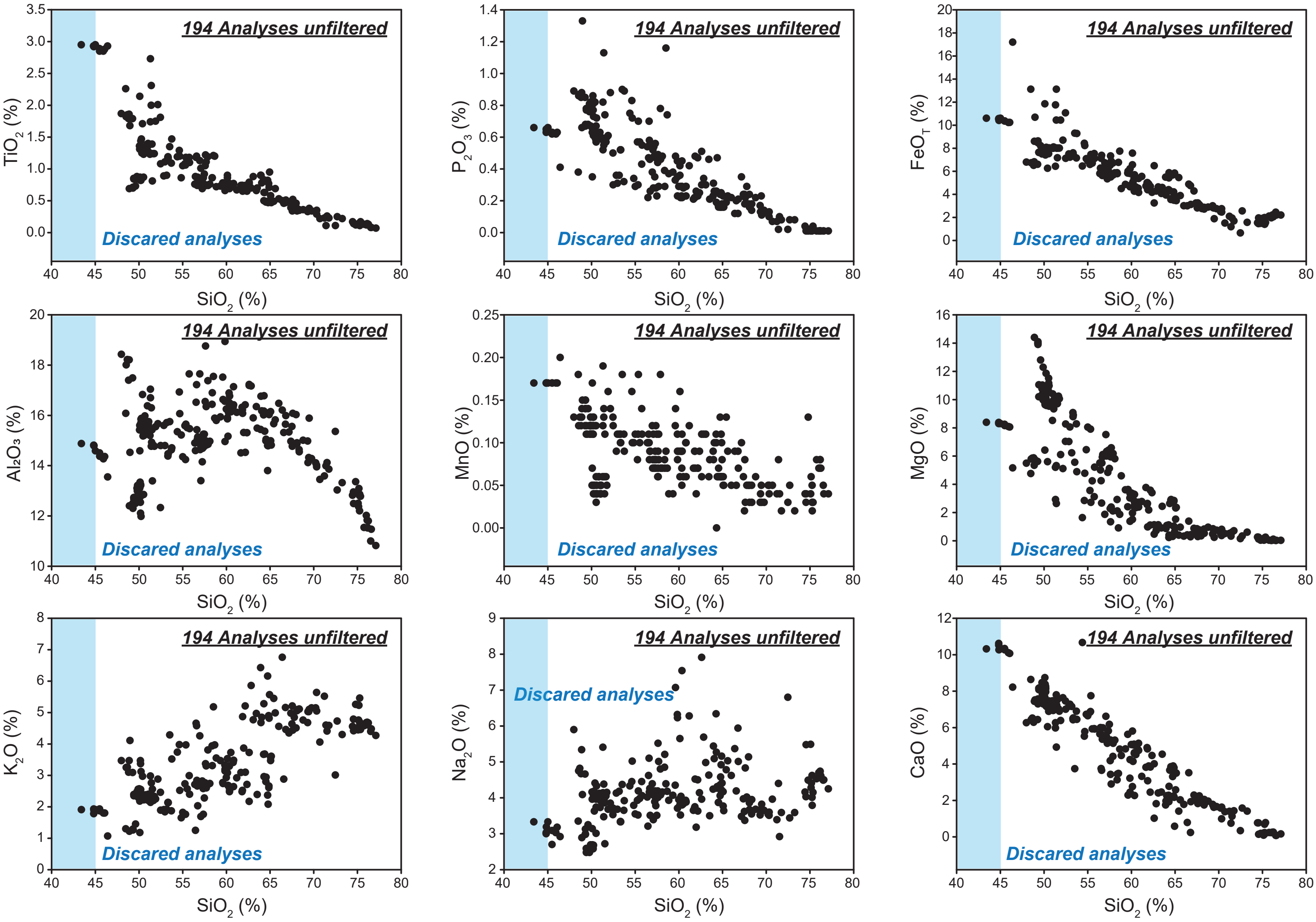

Yanshan Mountains 125-122 Ma Arc Segment GAME Results

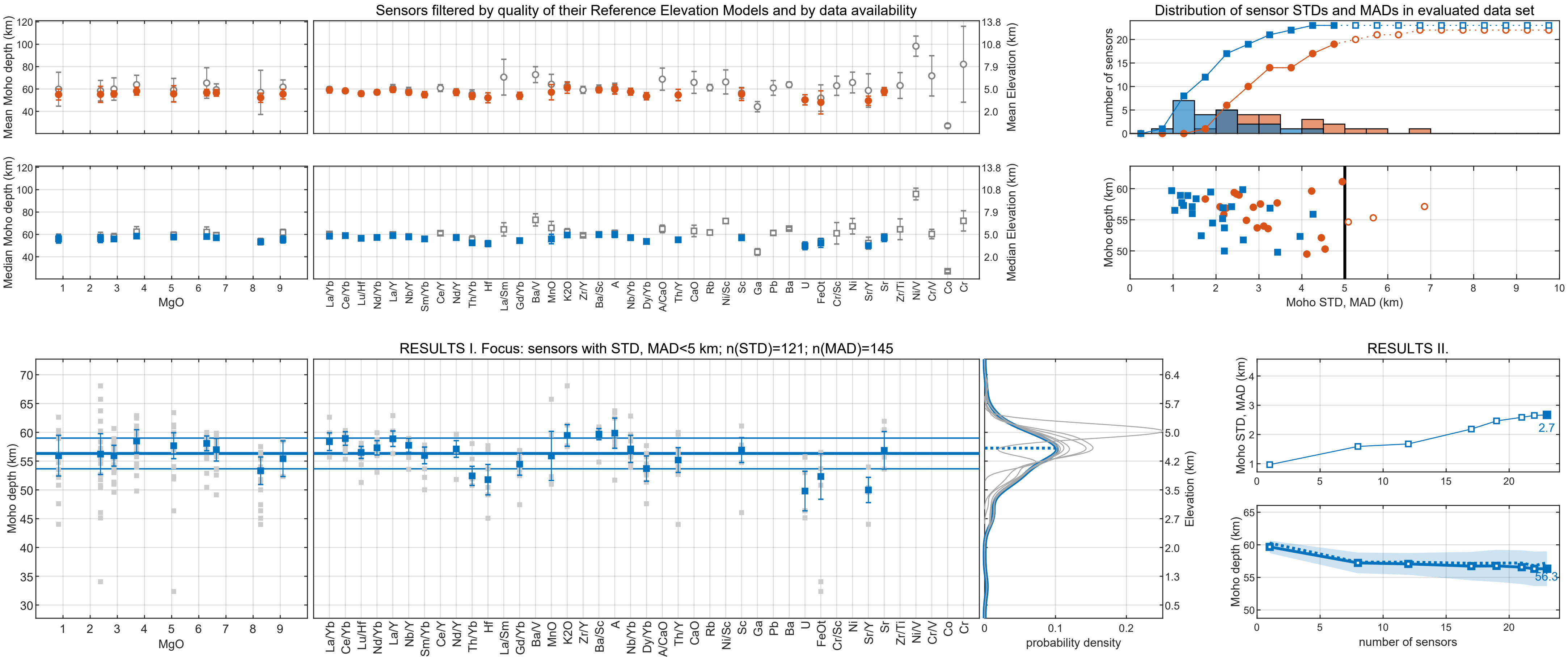

Figure S5-15 Southern Great Xing'an Range (SGXR): 160-140 Ma Magmatism

Individual Elevation vs. Latitude (150-140 Ma) and (160-150 Ma)

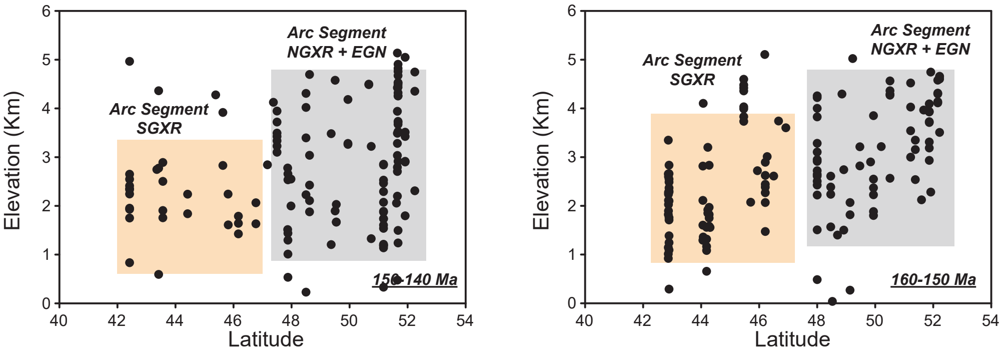

TAS diagram (160-140 Ma)

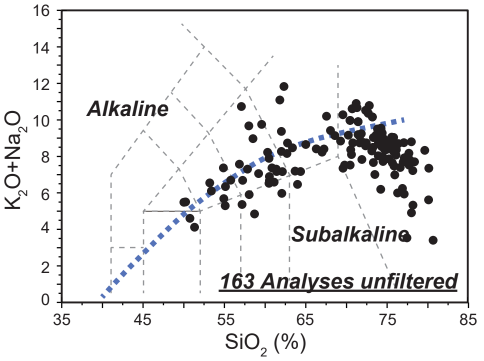

Individual Elevation vs. Latitude (170-160 Ma) and (180-170 Ma)

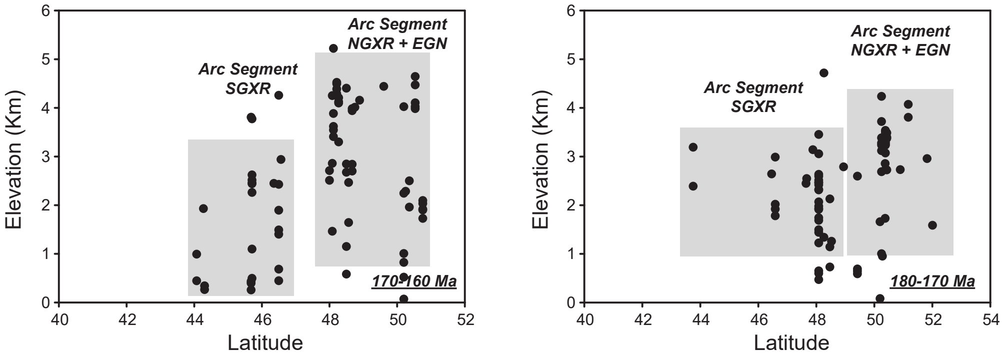

TAS diagram (160-140 Ma)

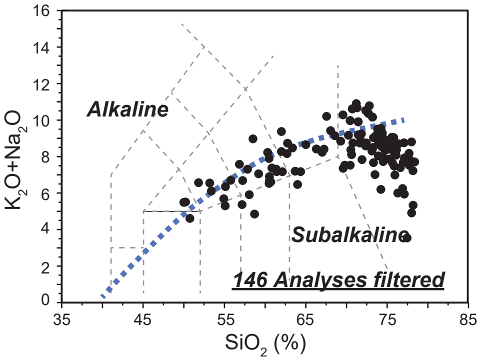

Harker Diagrams (160-140 Ma)

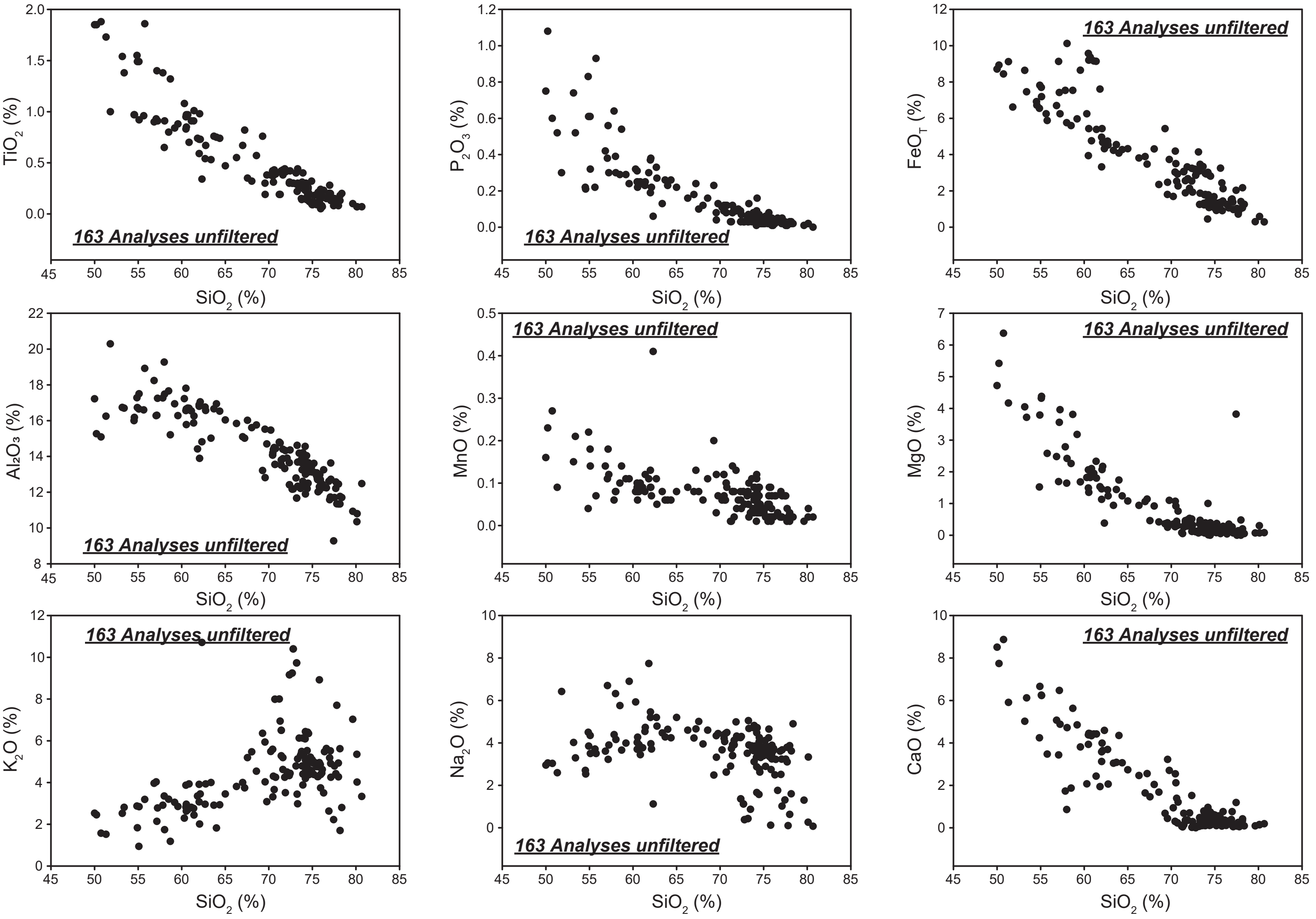

Southern Great Xing'an Range 160-140 Ma Arc Segment GAME Results

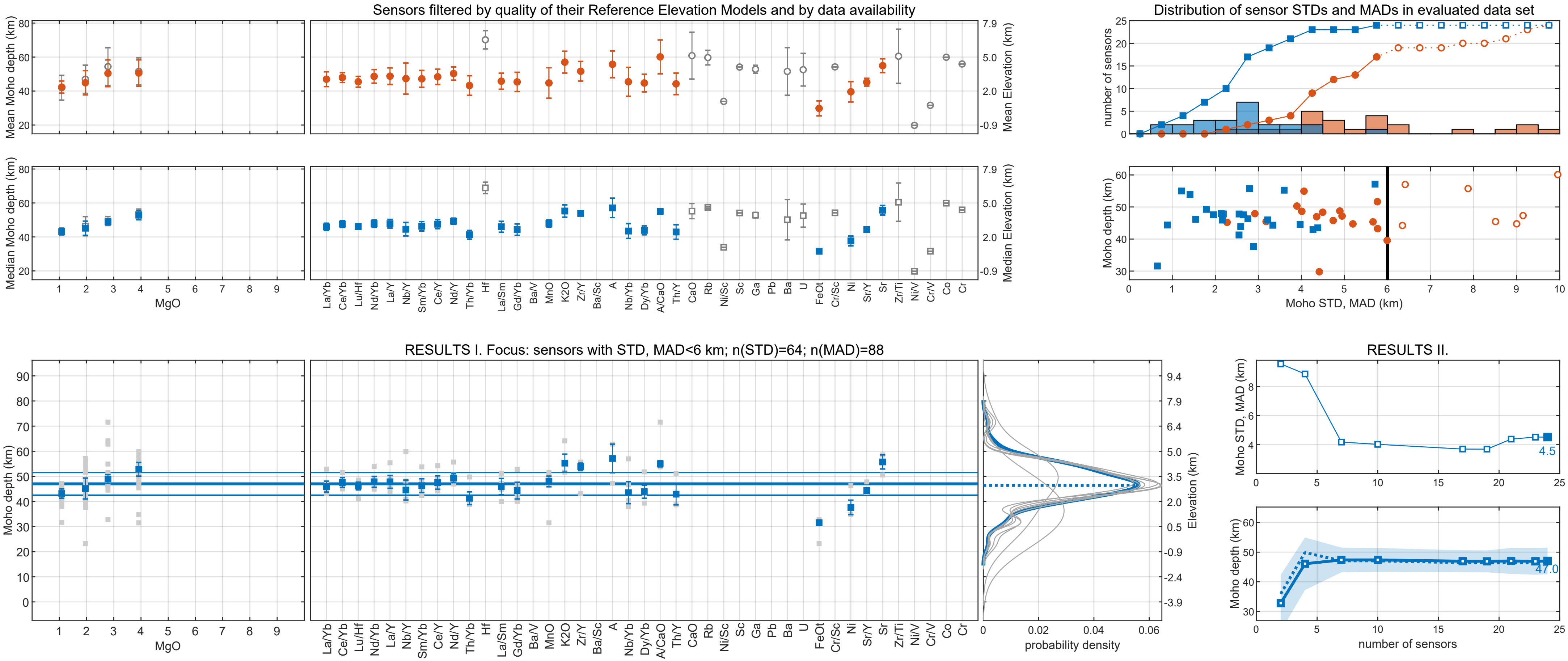

Figure S5-16 Southern Great Xing'an Range (SGXR): 140-130 Ma Magmatism

Individual Elevation vs. Age (140-130 Ma)

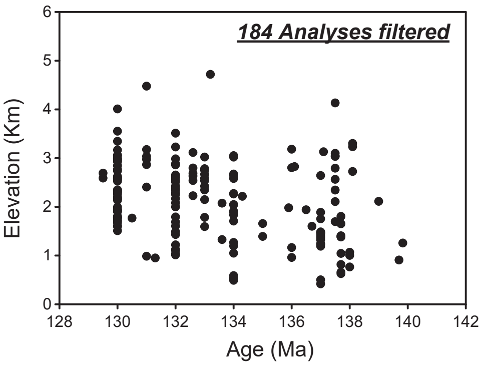

TAS diagram (140-130 Ma)

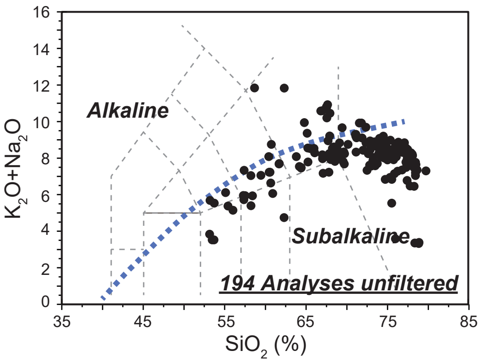

Individual Elevation vs. Longtitude and Latitude (140-115 Ma) and (140-130 Ma)

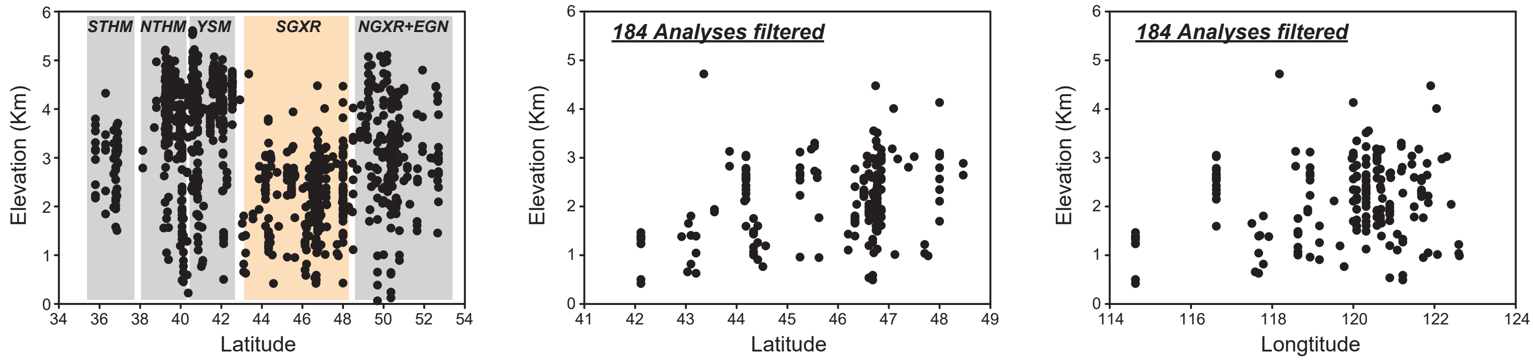

Harker Diagrams (140-130 Ma)

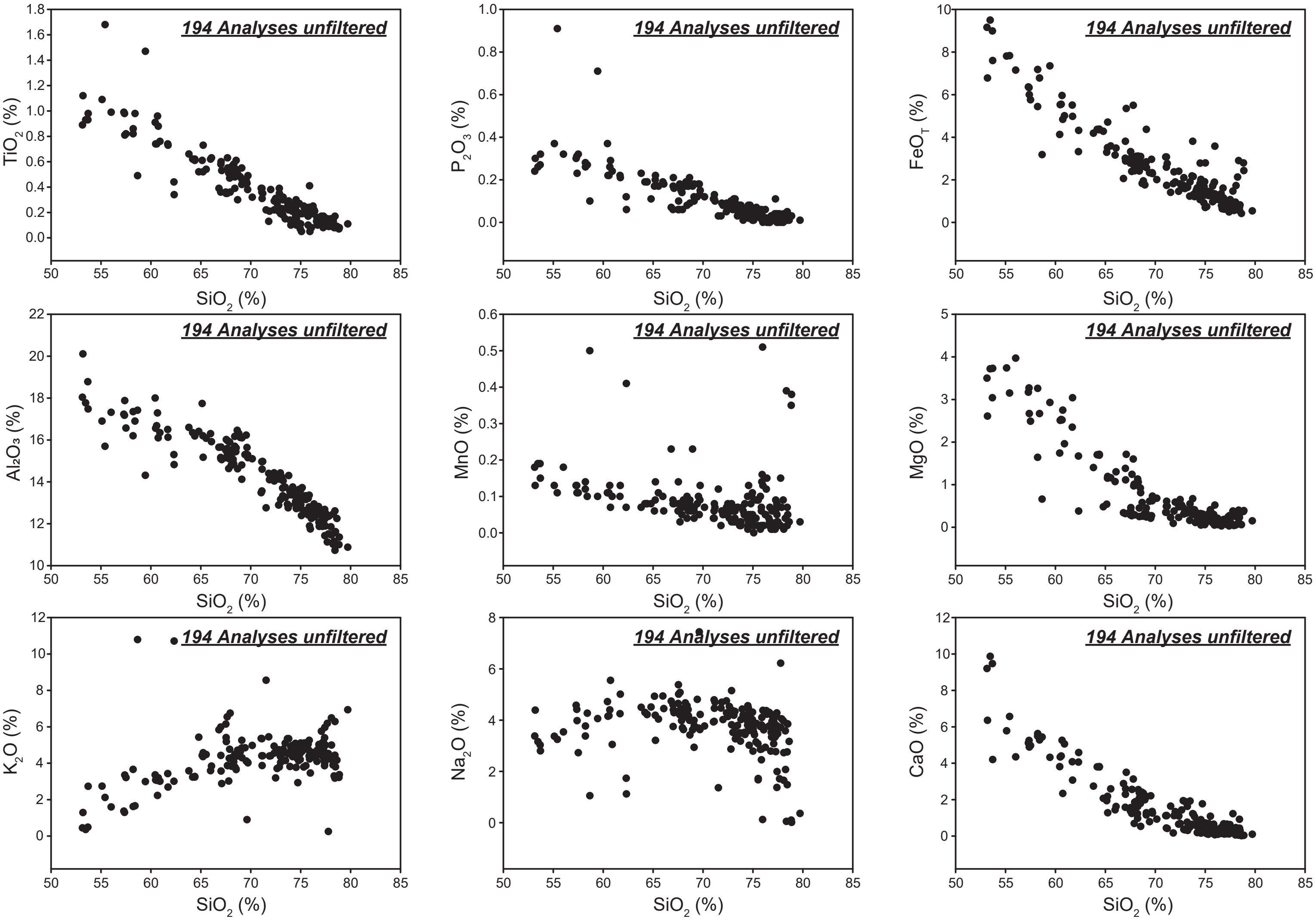

Southern Great Xing’an Range 140-130 Ma Arc Segment GAME Results

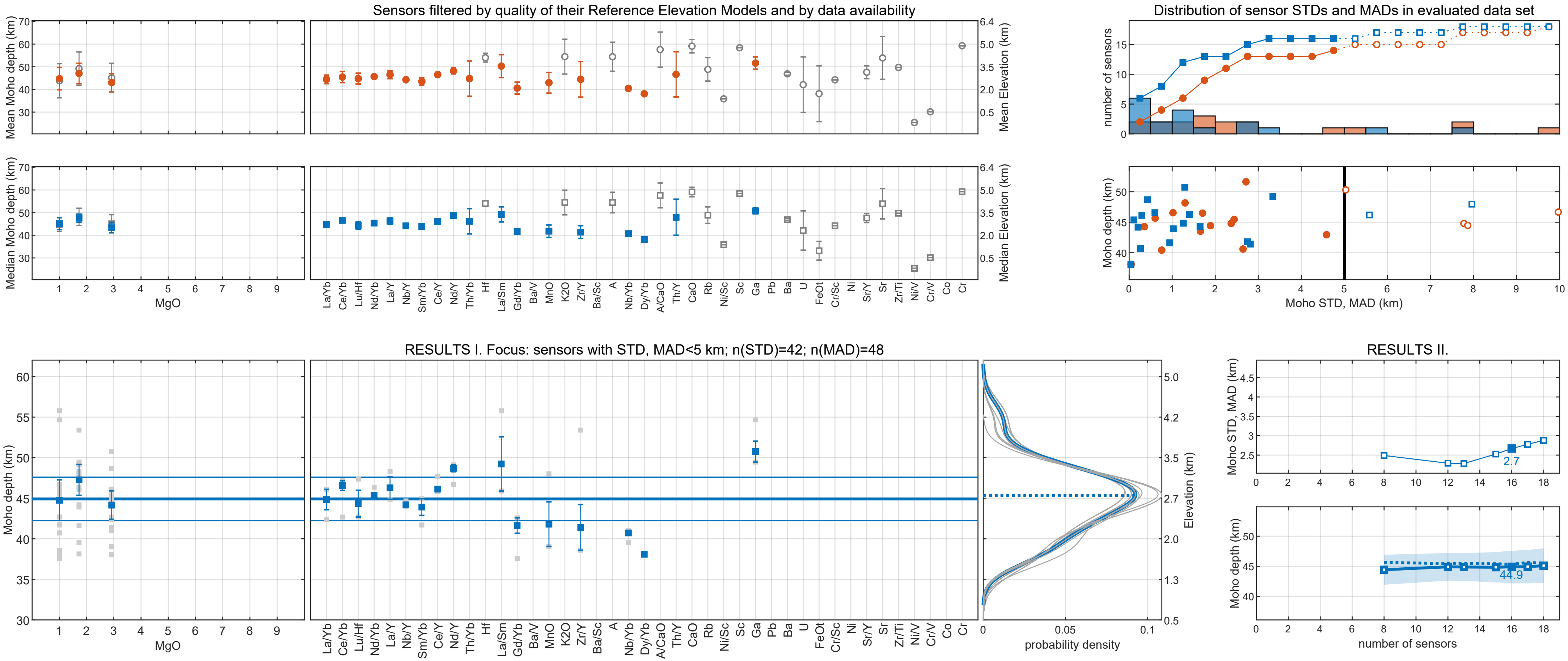

Figure S5-17 Southern Great Xing'an Range (SGXR): 130-120 Ma Magmatism

Individual Elevation vs. Age (130-120 Ma)

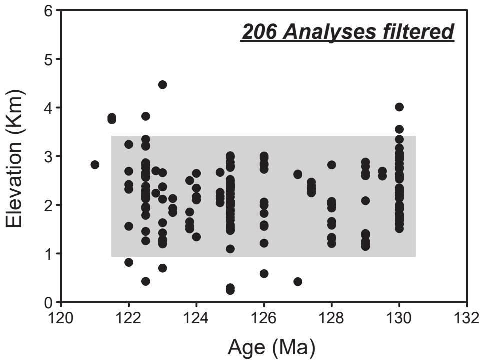

TAS diagram (130-120 Ma)

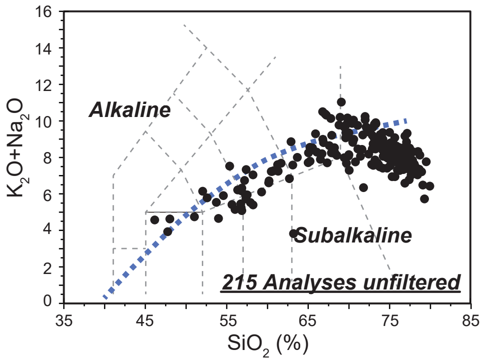

Individual Moho Depth vs. Longtitude and Latitude (140-115 Ma) and (130-120 Ma)

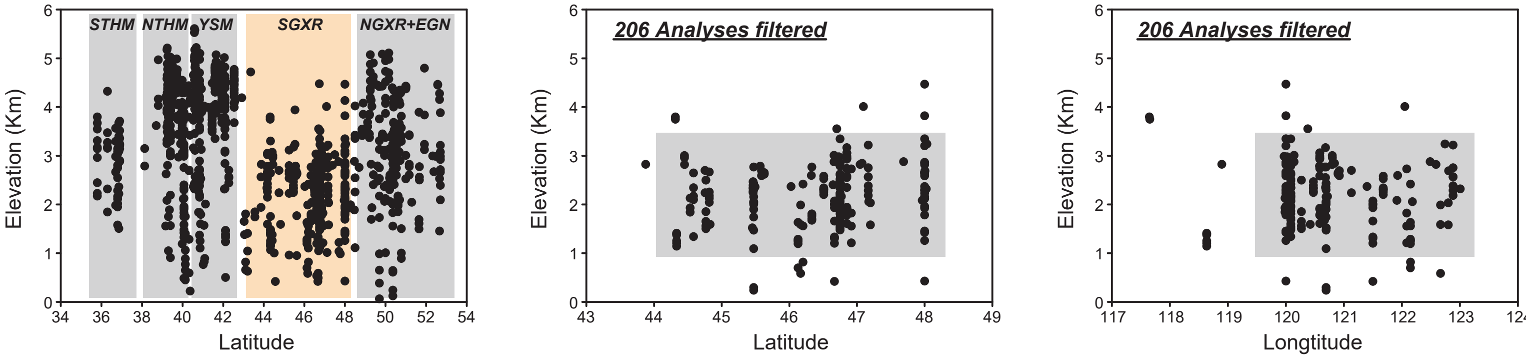

Harker Diagrams (130-120 Ma)

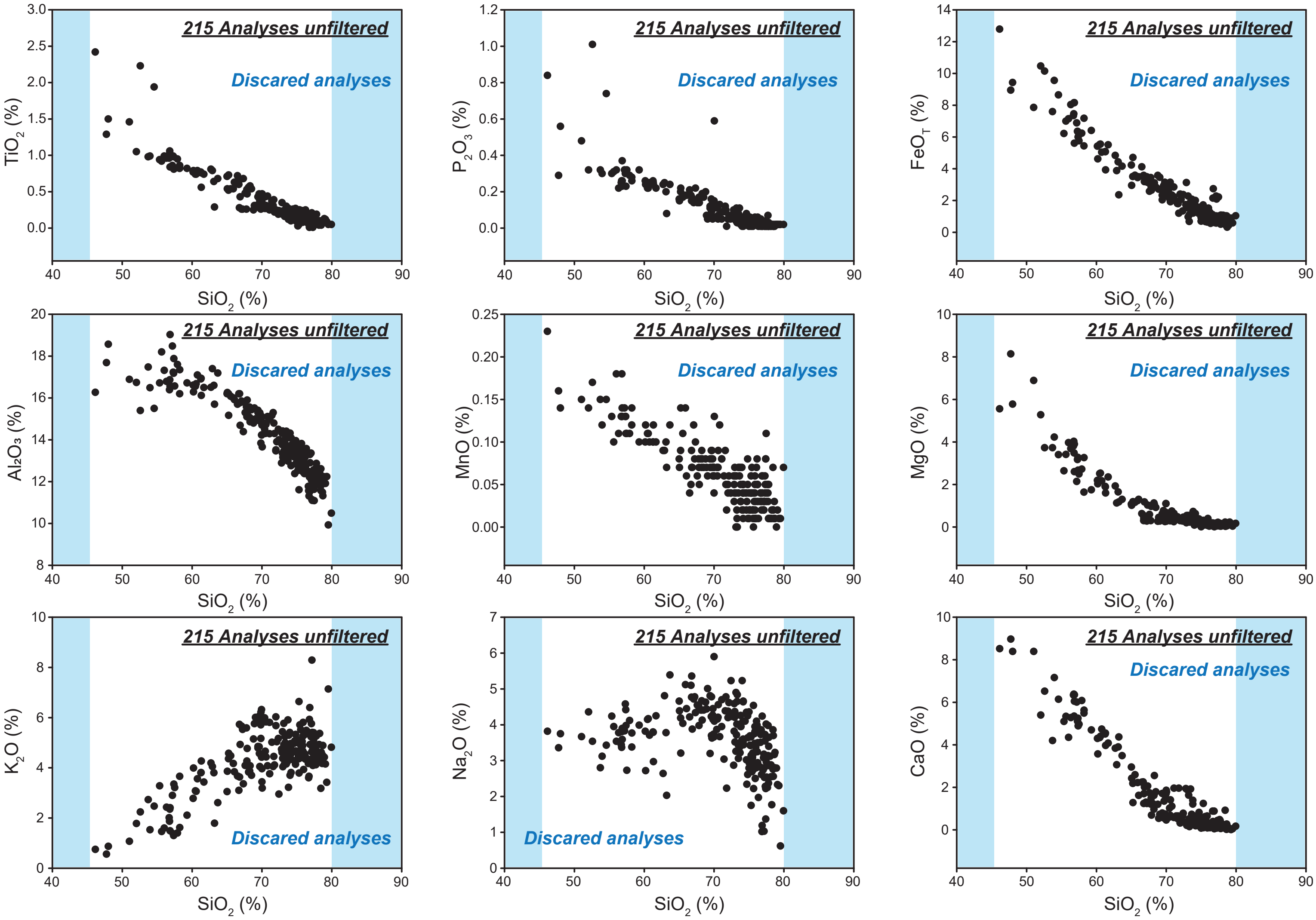

Southern Great Xing'an Range 130-120 Ma Arc Segment GAME Results

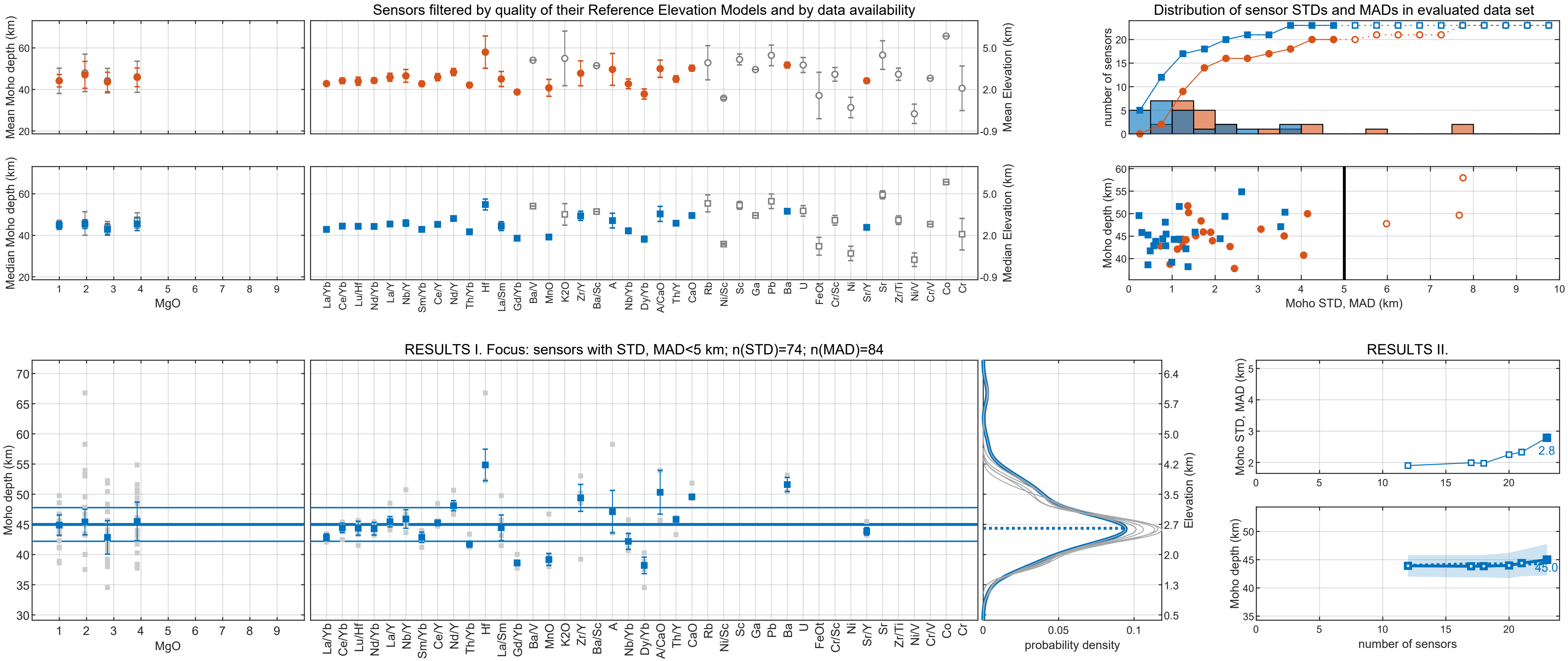

Figure S5-18 Northern Great Xing'an Range (NGXR) and Ergna Block (EGN): 206-186 Ma Magmatism

Individual Elevation vs. Age (206-160 Ma)

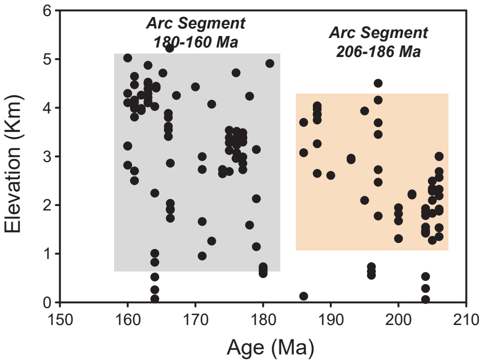

TAS diagram (206-186 Ma)

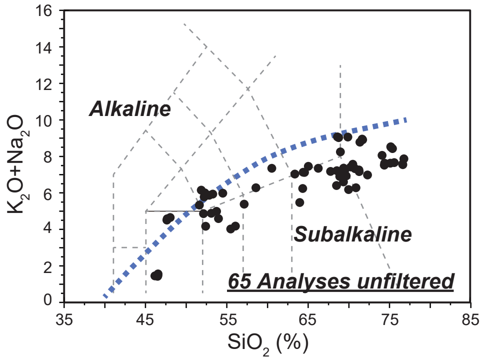

Individual Elevation vs. Longtitude and Latitude (206-186 Ma)

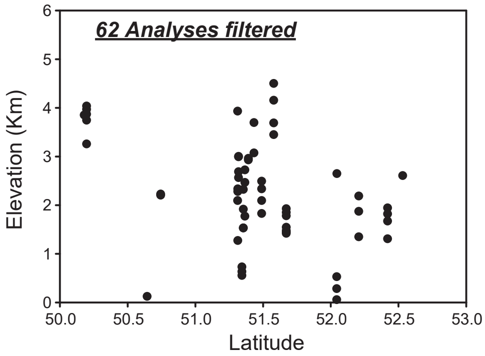

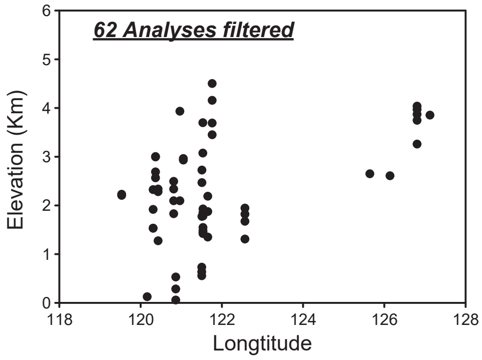

Harker Diagrams (206-186 Ma)

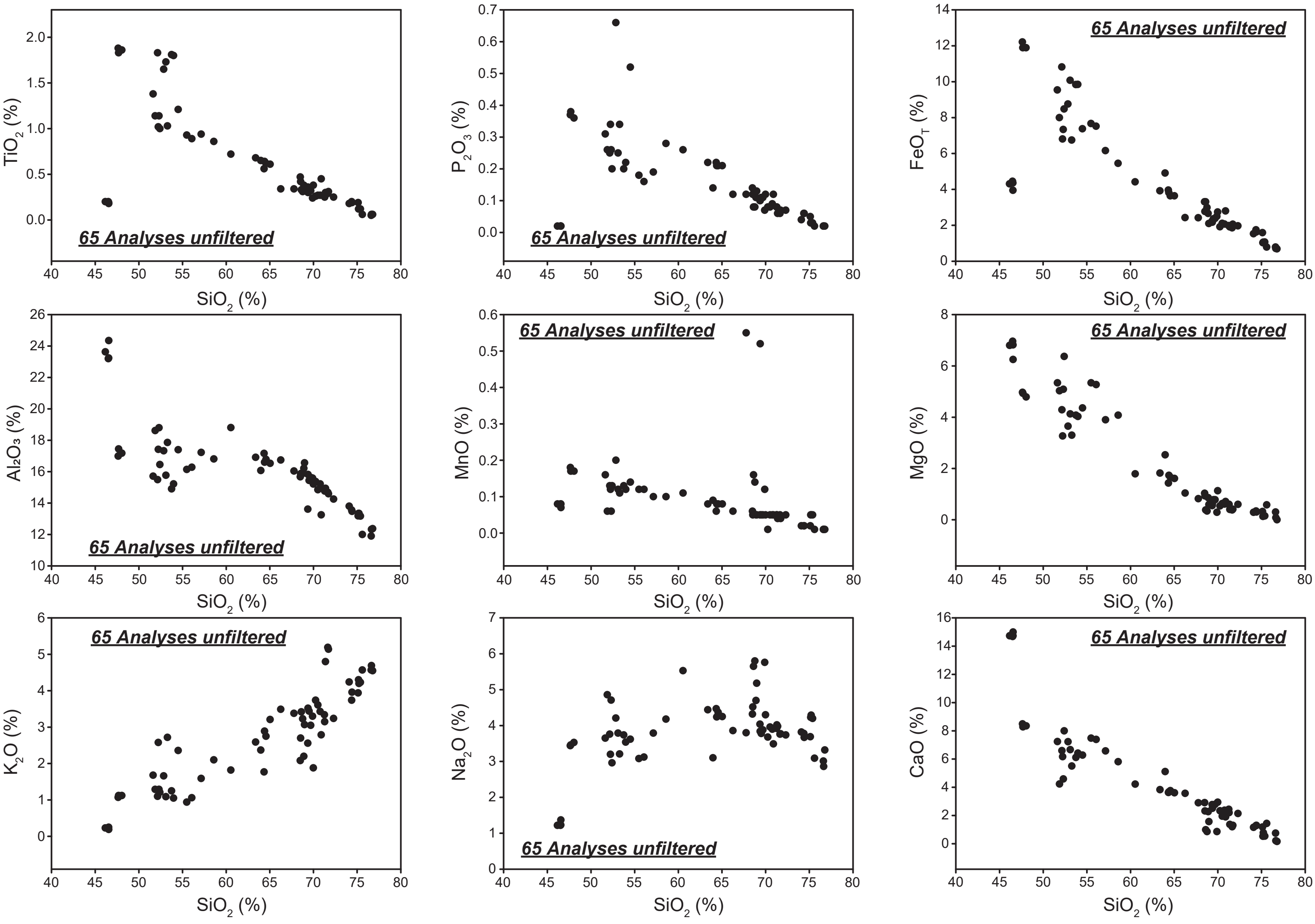

Northern Great Xing'an Range and Erguna Region 206-186 Ma Arc Segment GAME Results

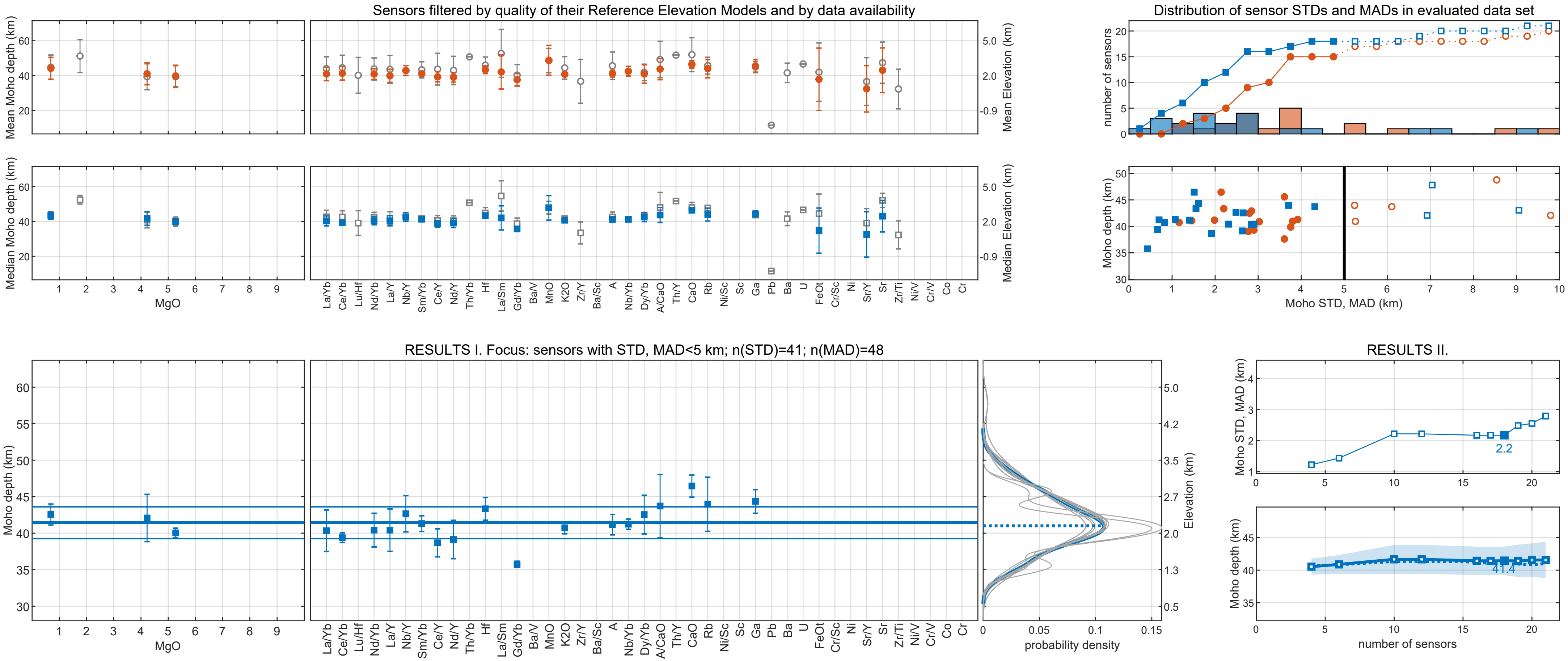

Figure S5-19 Northern Great Xing'an Range (NGXR) and Ergna Block (EGN): 180-160 Ma Magmatism

Individual Elevation vs. Latitude (150-140 Ma) and (160-150 Ma)

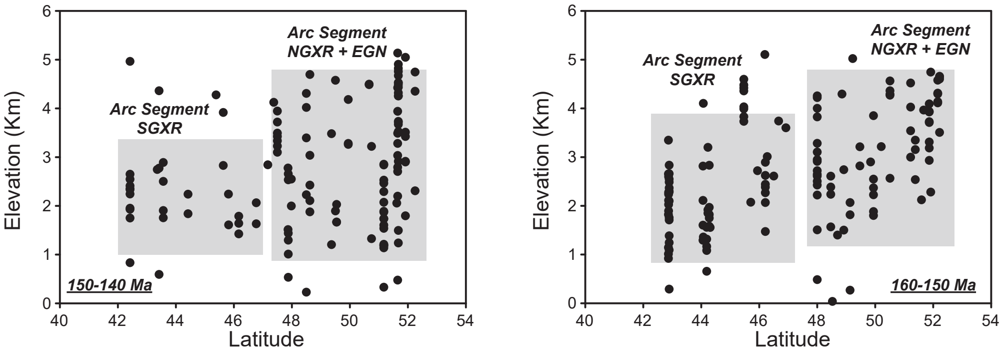

TAS diagram (180-160 Ma)

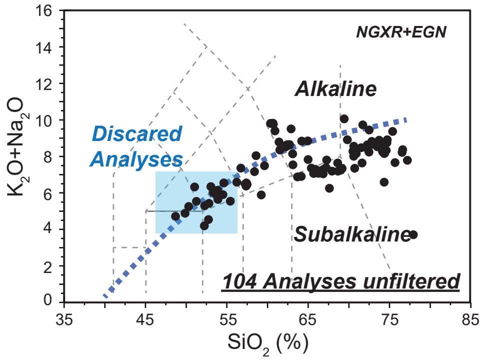

Individual Elevation vs. Latitude (170-160 Ma) and (180-170 Ma)

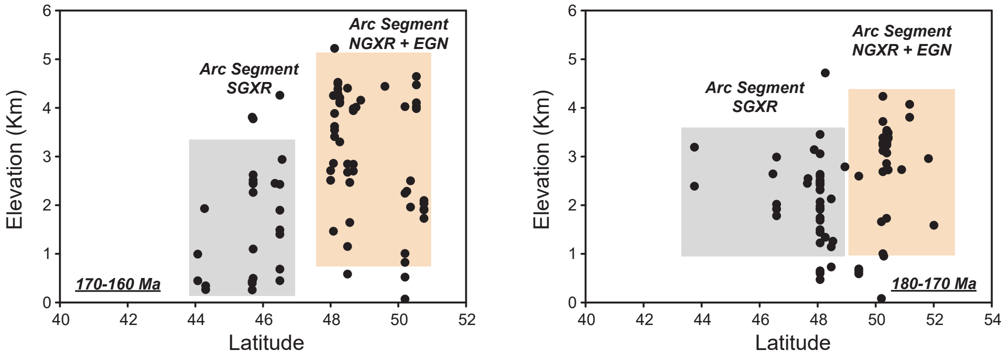

TAS diagram (180-160 Ma)

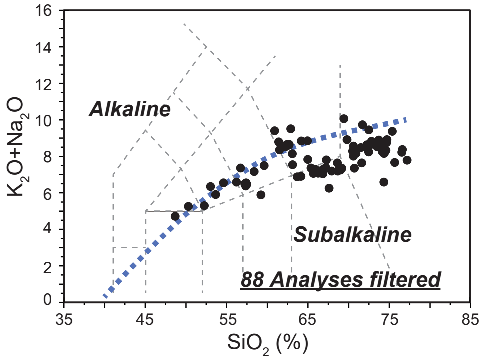

Harker Diagrams (180-160 Ma)

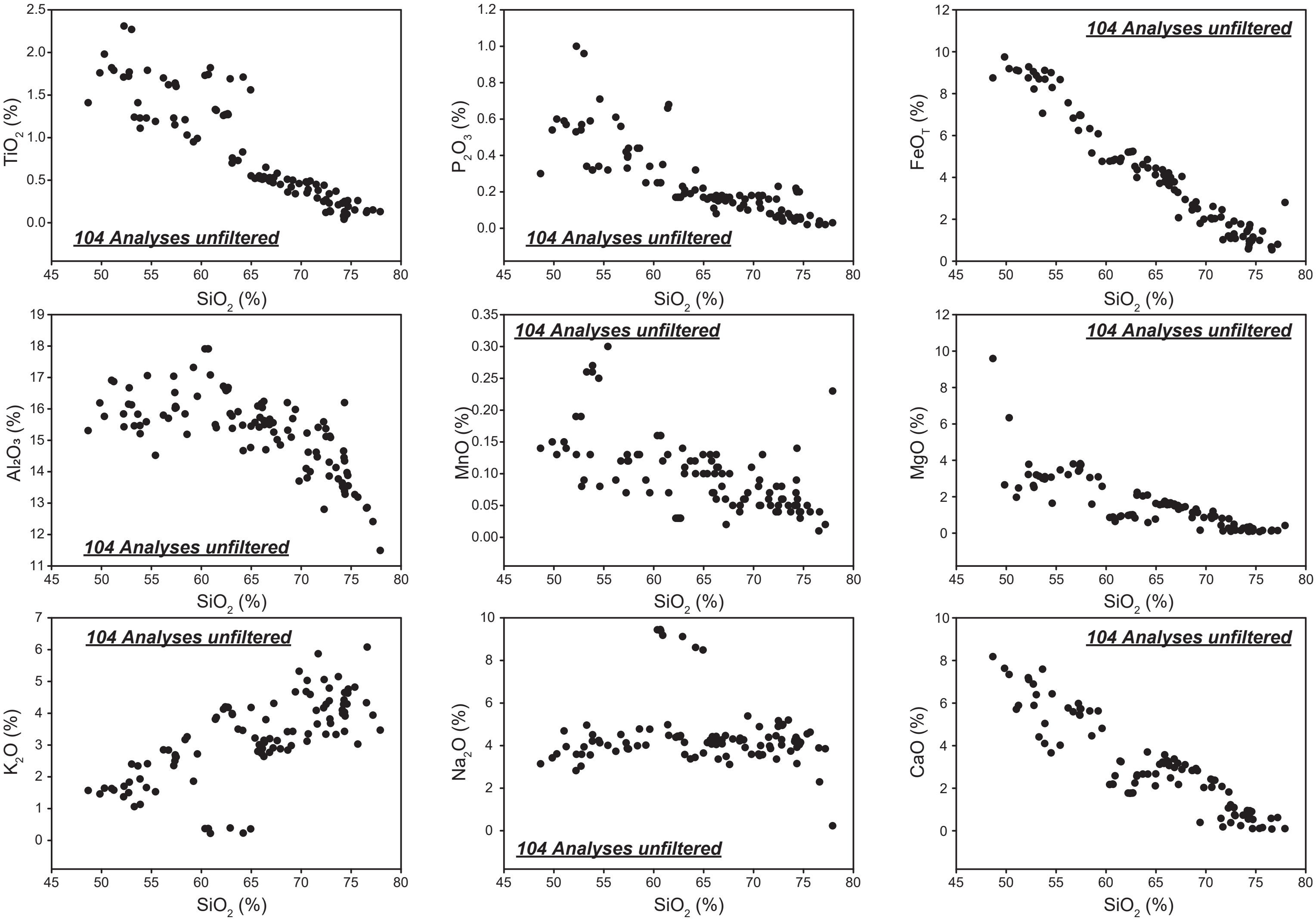

Northern Great Xing'an Range and Erguna Region 180-160 Ma Arc Segment GAME Results

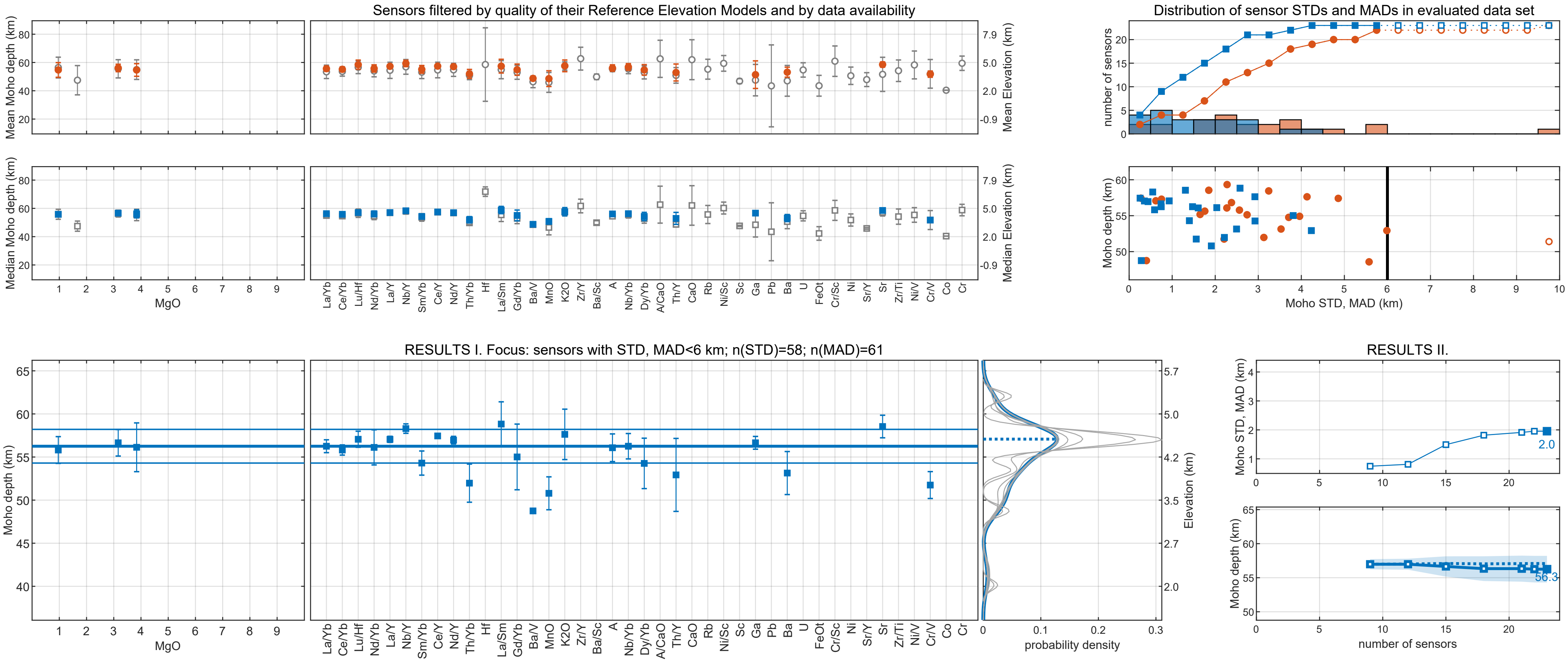

Figure S5-20 Northern Great Xing'an Range (NGXR) and Ergna Block (EGN): 143-128 Ma Magmatism

Individual Elevation vs. Age (143-112 Ma) TAS diagram (143-128 Ma)

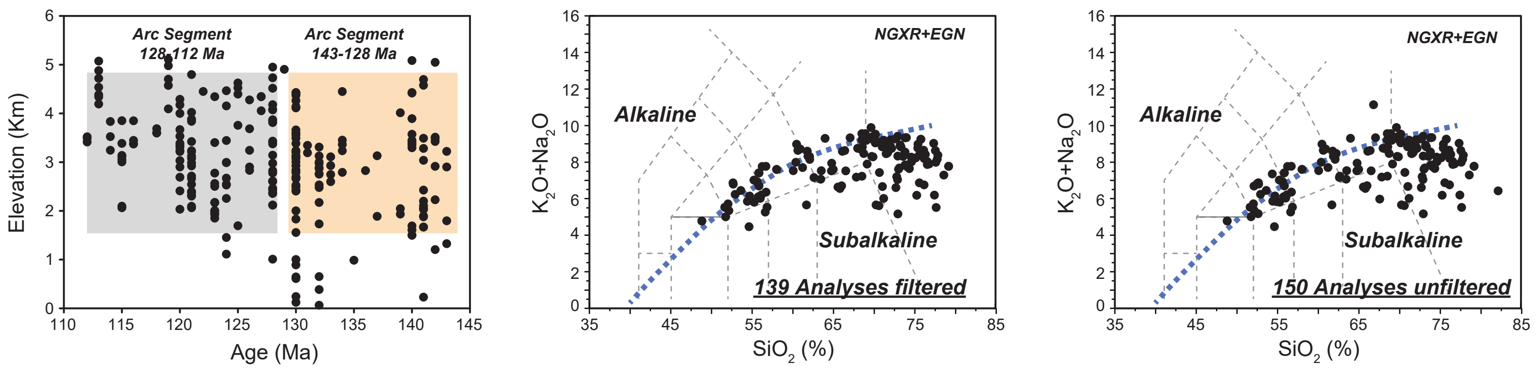

Individual Elevation vs. Longtitude and Latitude (140-115 Ma) and (143-128 Ma)

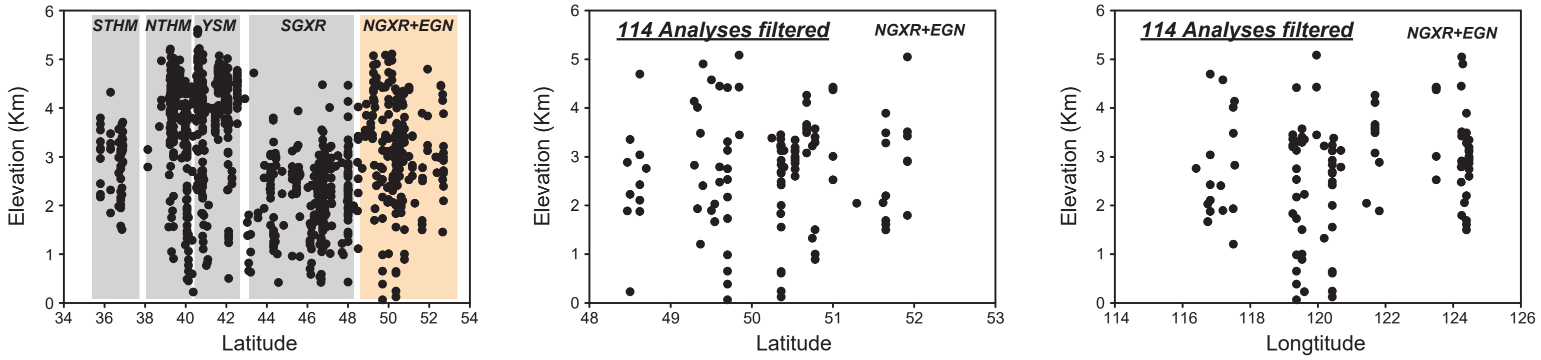

Harker Diagrams (143-128 Ma)

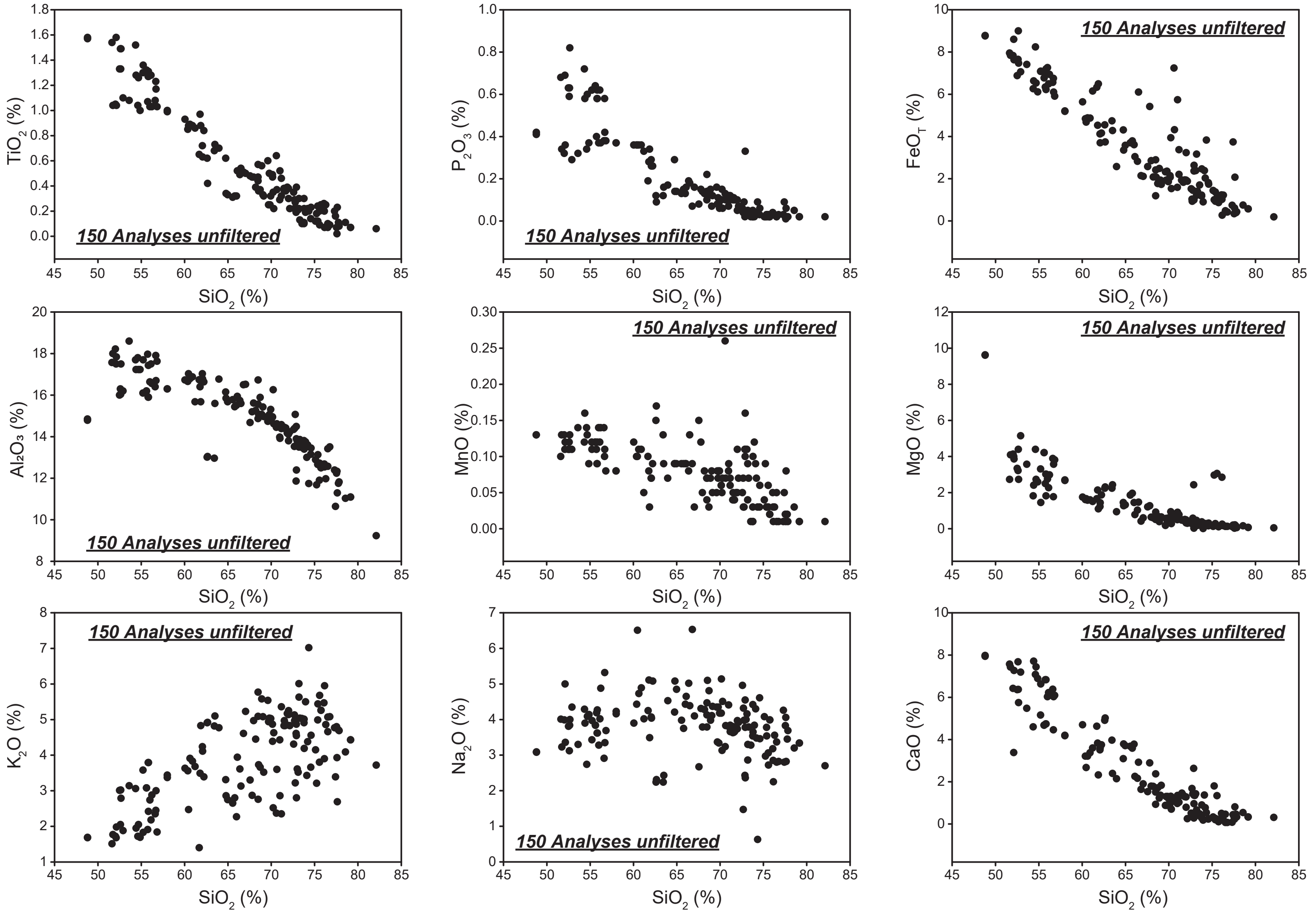

Northern Great Xing’an Range and Erguna Region 143-128 Ma Arc Segment GAME Results

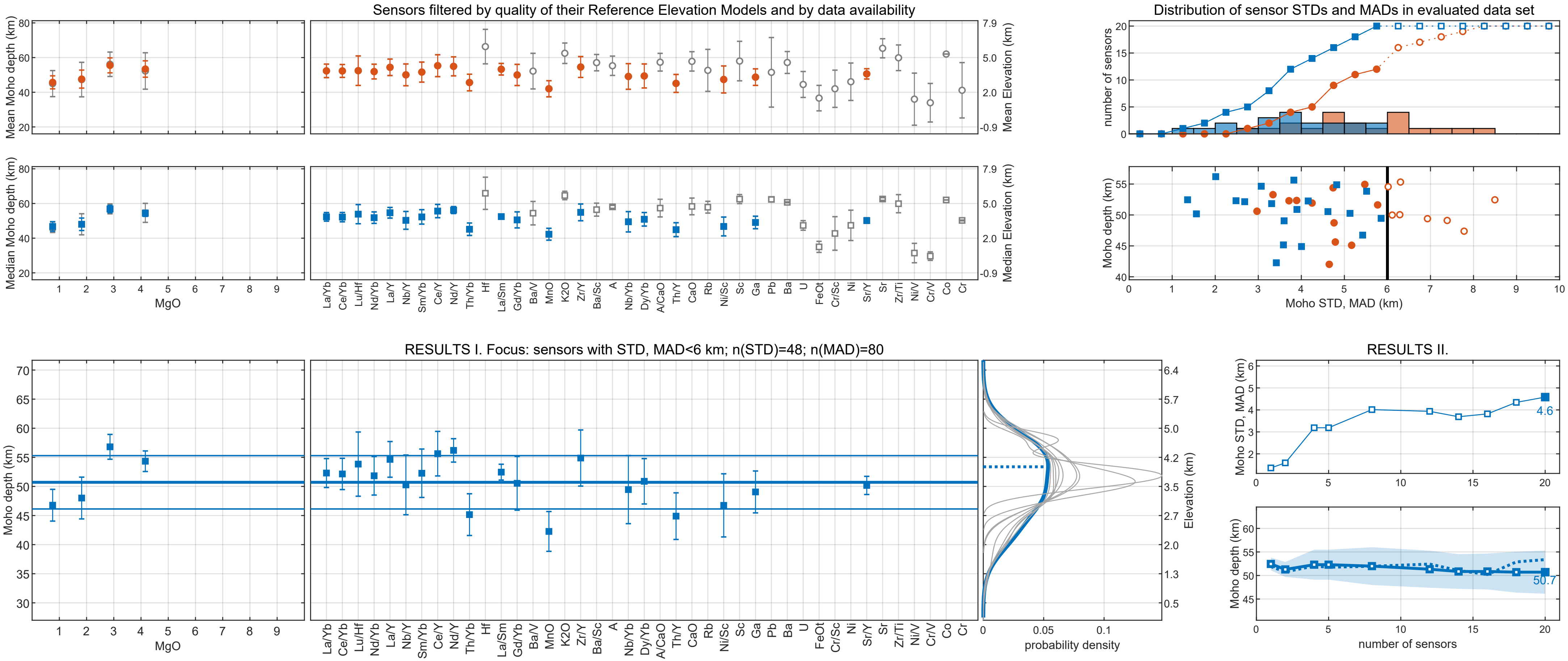

Figure S5-21 Northern Great Xing'an Range (NGXR) and Ergna Block (EGN): 128-112 Ma Magmatism

Individual Elevation vs. Age (143-112 Ma) TAS diagram (128-112 Ma)

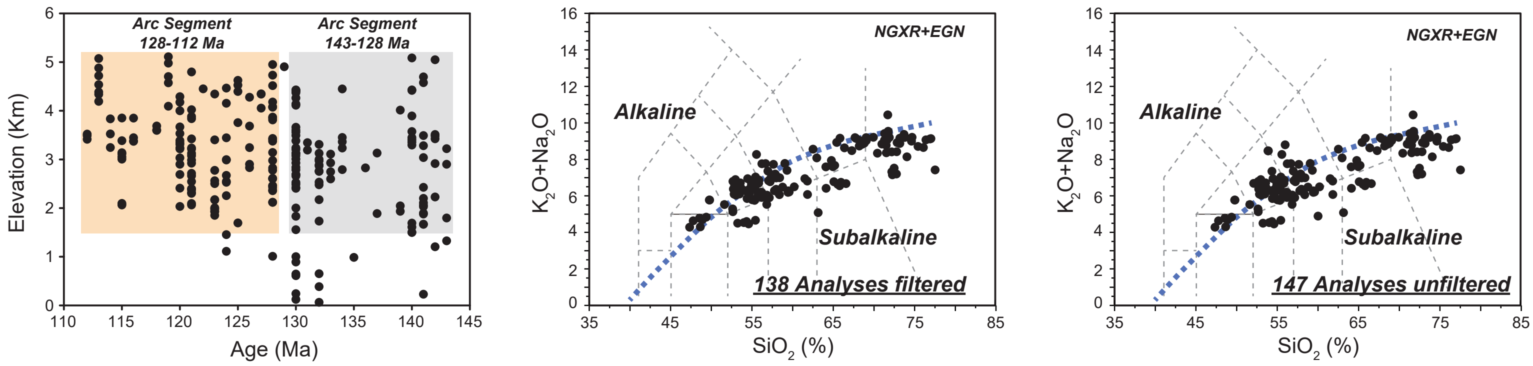

Individual Elevation vs. Longtitude and Latitude (140-115 Ma) and (128-112 Ma)

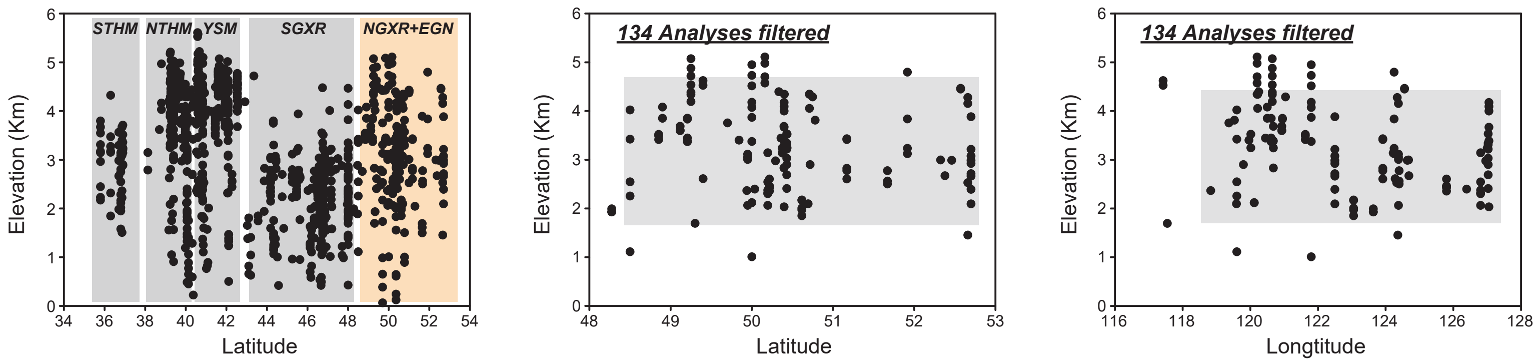

Harker Diagrams (128-112 Ma)

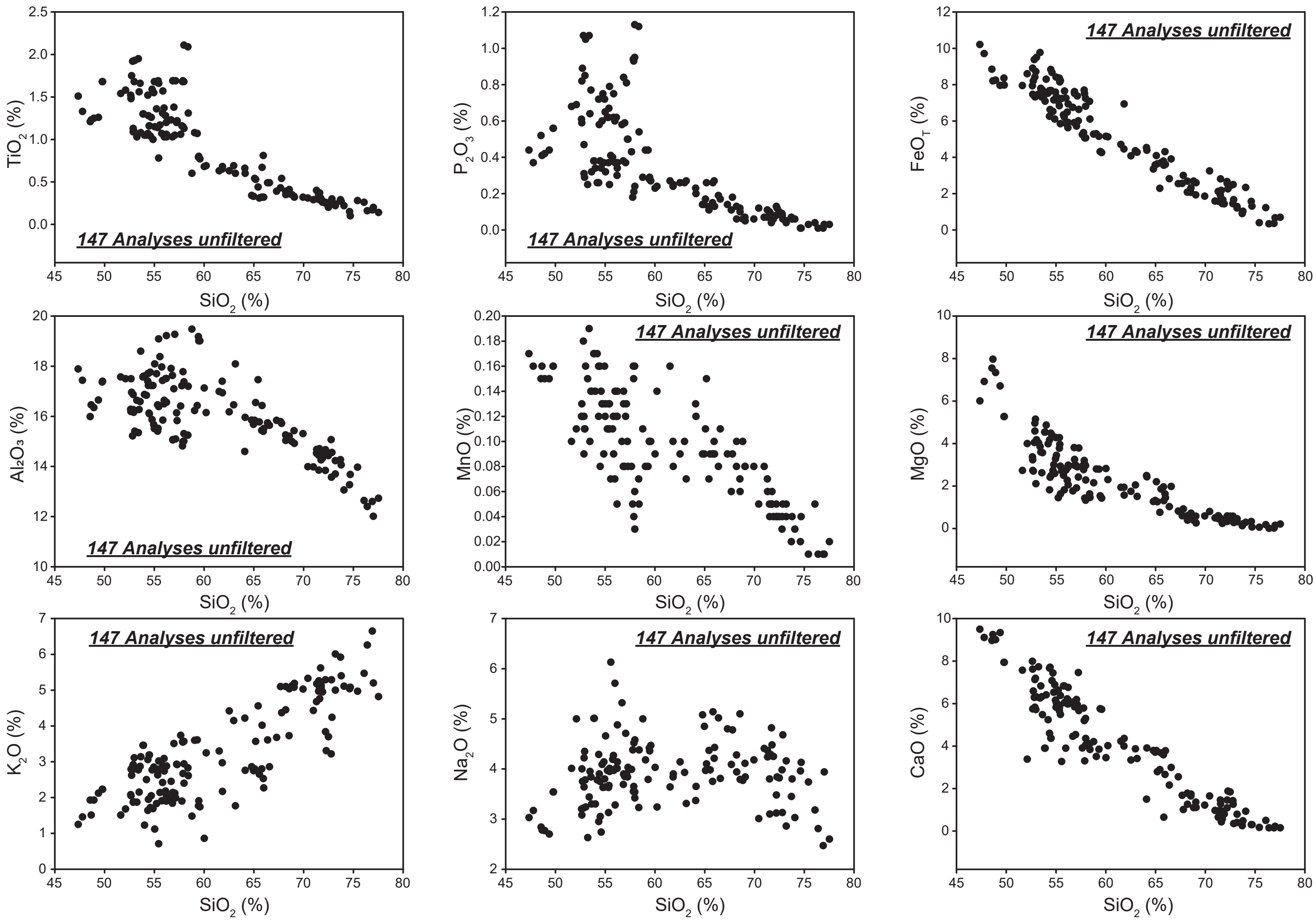

Northern Great Xing'an Range and Erguna Region 128-112 Ma Arc Segment GAME Results

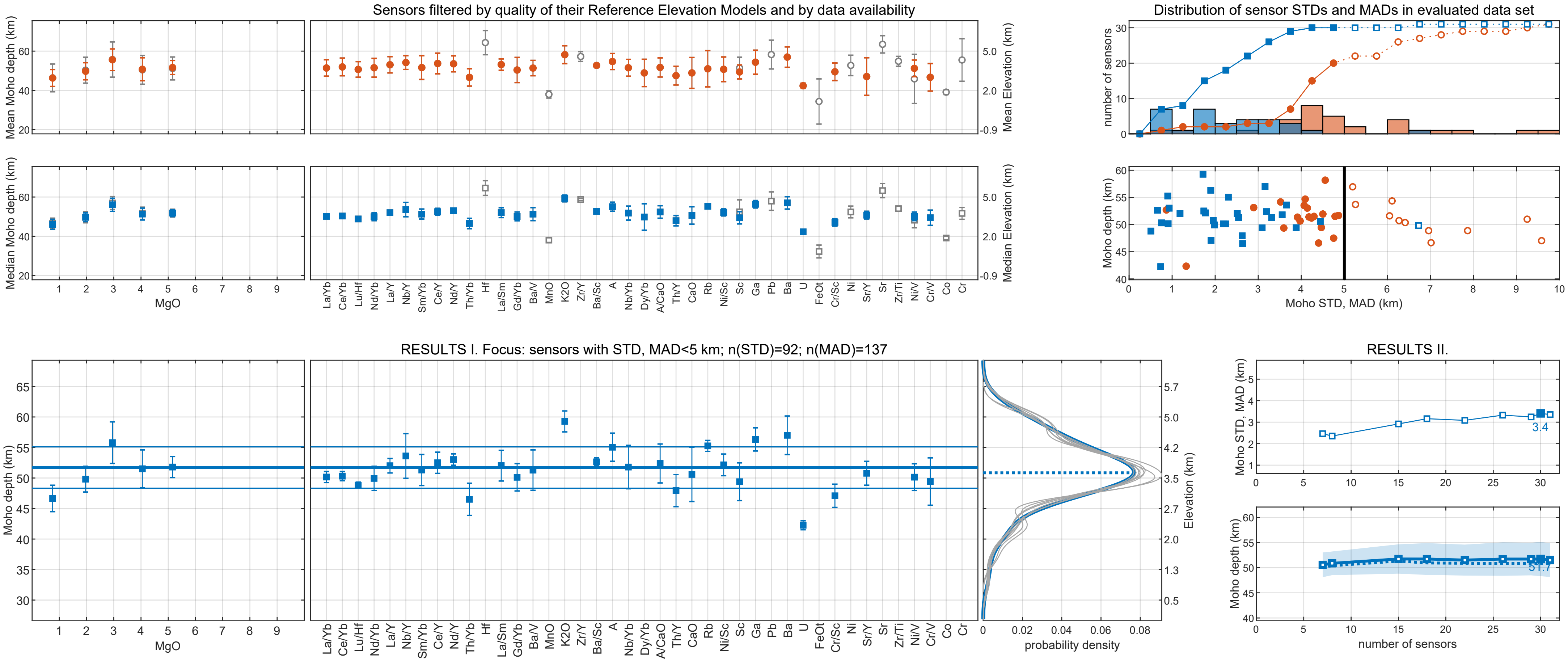

Figure S5-22 Songliao Basin (SB): 115-102 Ma Magmatism

Individual Elevation vs. Age (133-102 Ma)

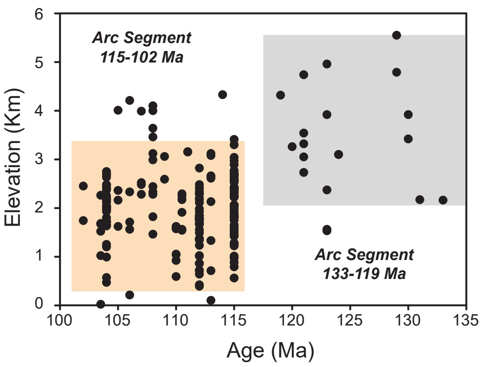

TAS diagram (115-102 Ma)

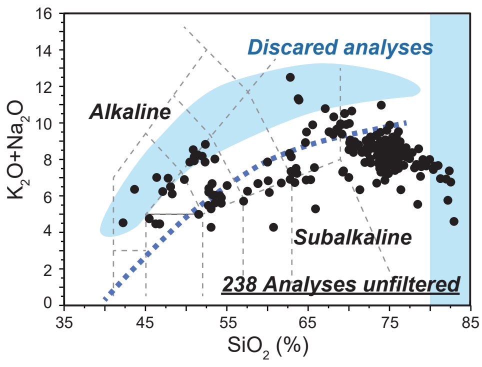

Individual Elevation vs. Longtitude and Latitude (115-102 Ma)

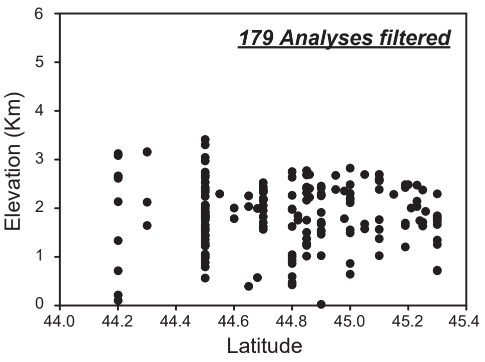

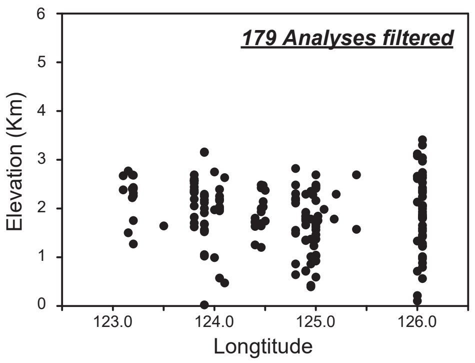

Harker Diagrams (115-102 Ma)

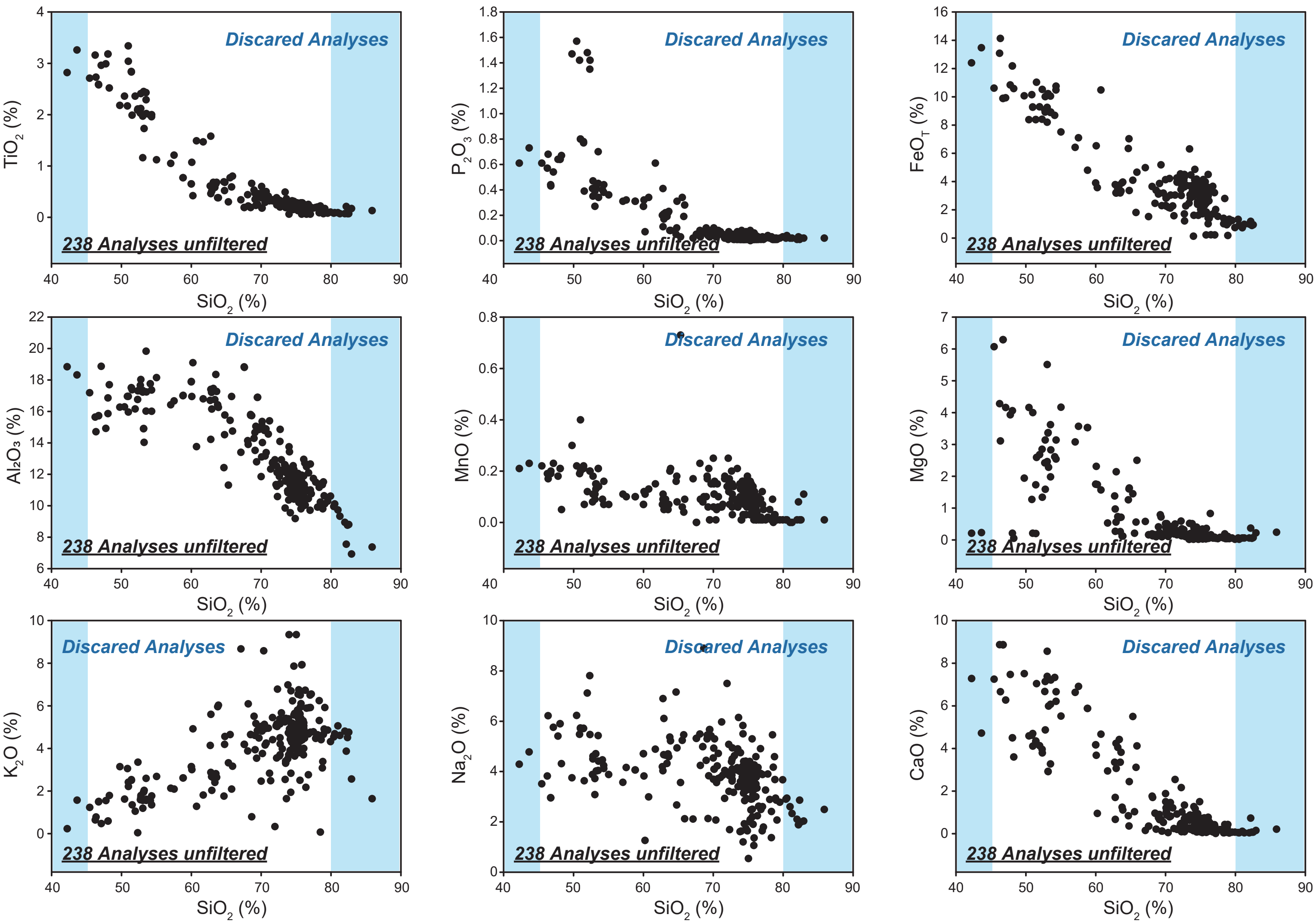

Songliao Basin 115-102 Ma Arc Segment GAME Results

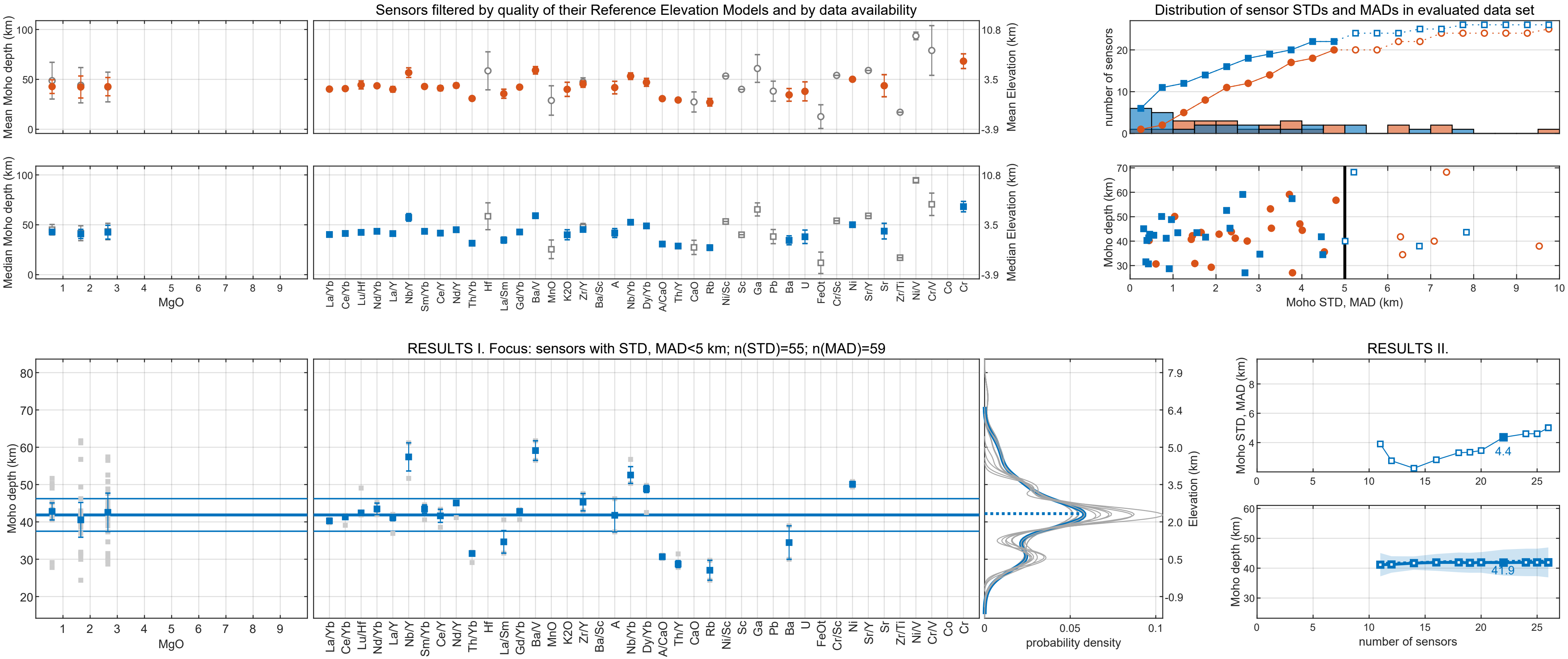

Figure S5-23 Zhangguangcai Range (ZGR) and Jiamusi Region (JR): 210-196 Ma Magmatism

Individual Elevation vs. Age (210-150 Ma) and (210-196 Ma)

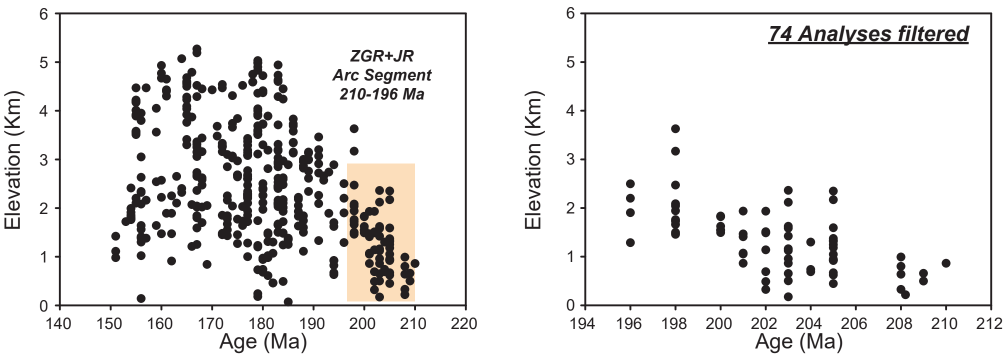

TAS diagram (210-196 Ma)

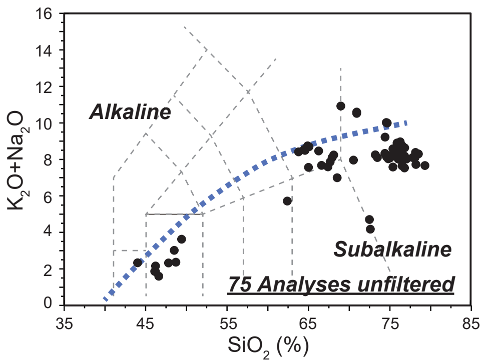

Individual Elevation vs. Latitude and Longtitude (210-196 Ma)

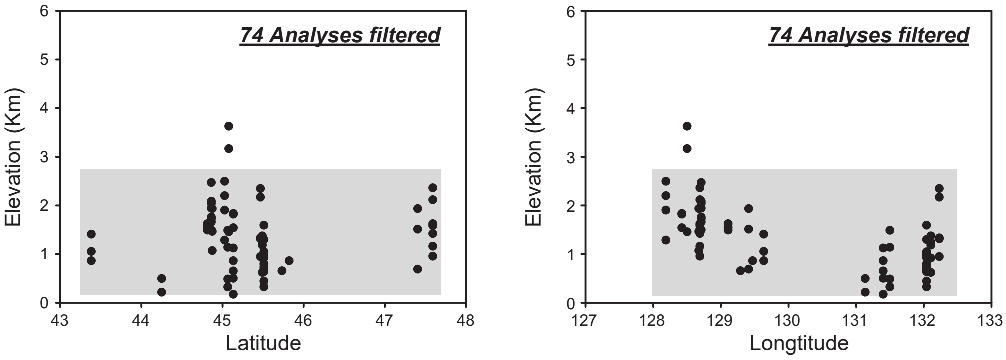

TAS diagram (210-196 Ma)

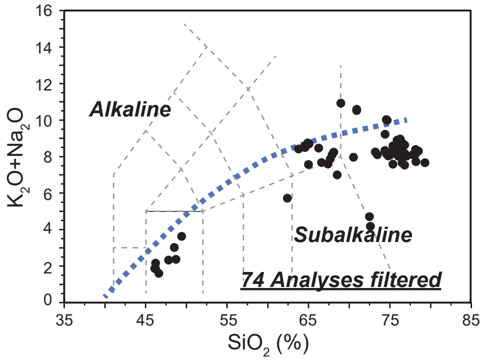

Harker Diagrams (210-196 Ma)

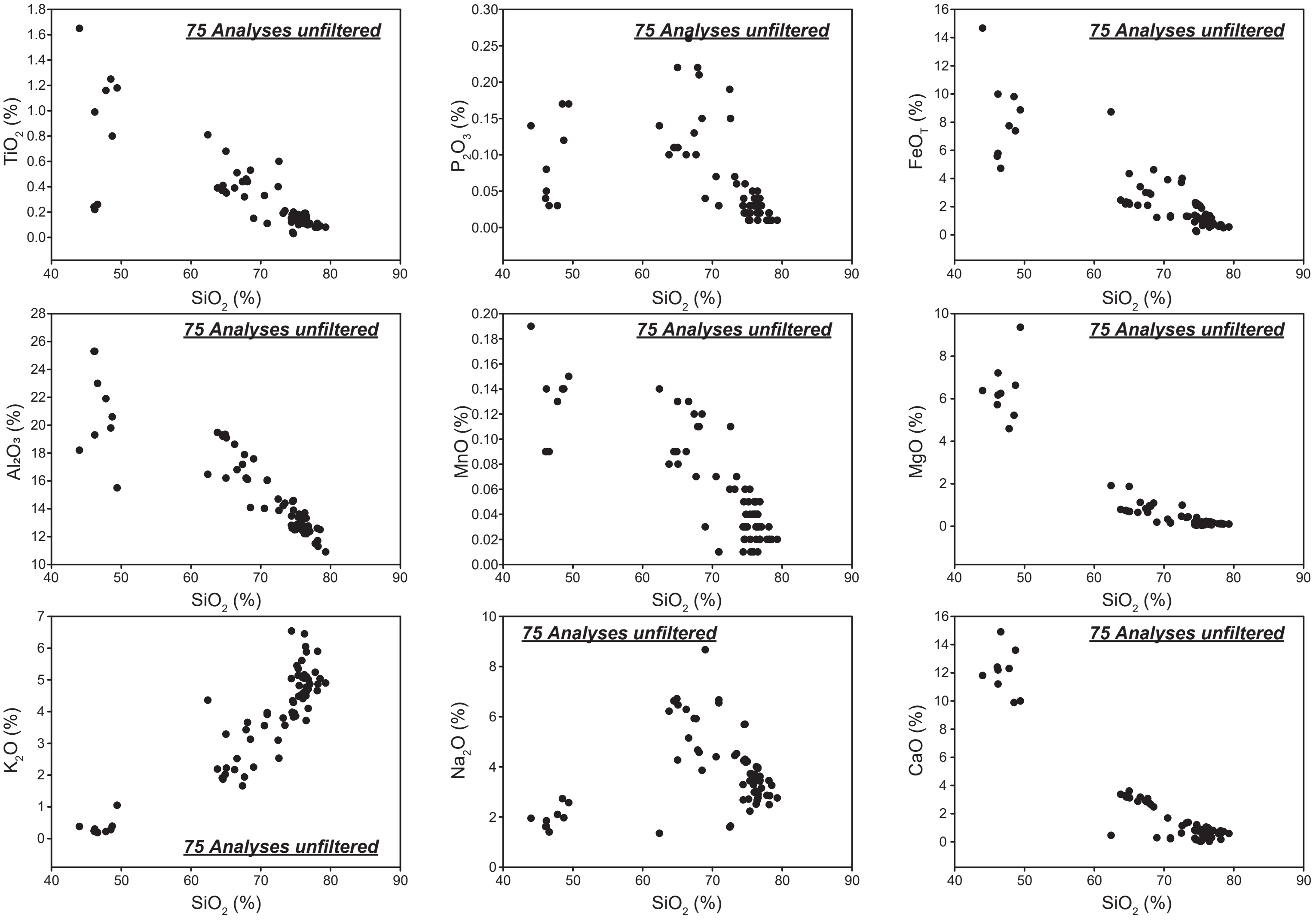

Zhangguangcai Range and Jiamusi Region 210-196 Ma Arc Segment GAME Results

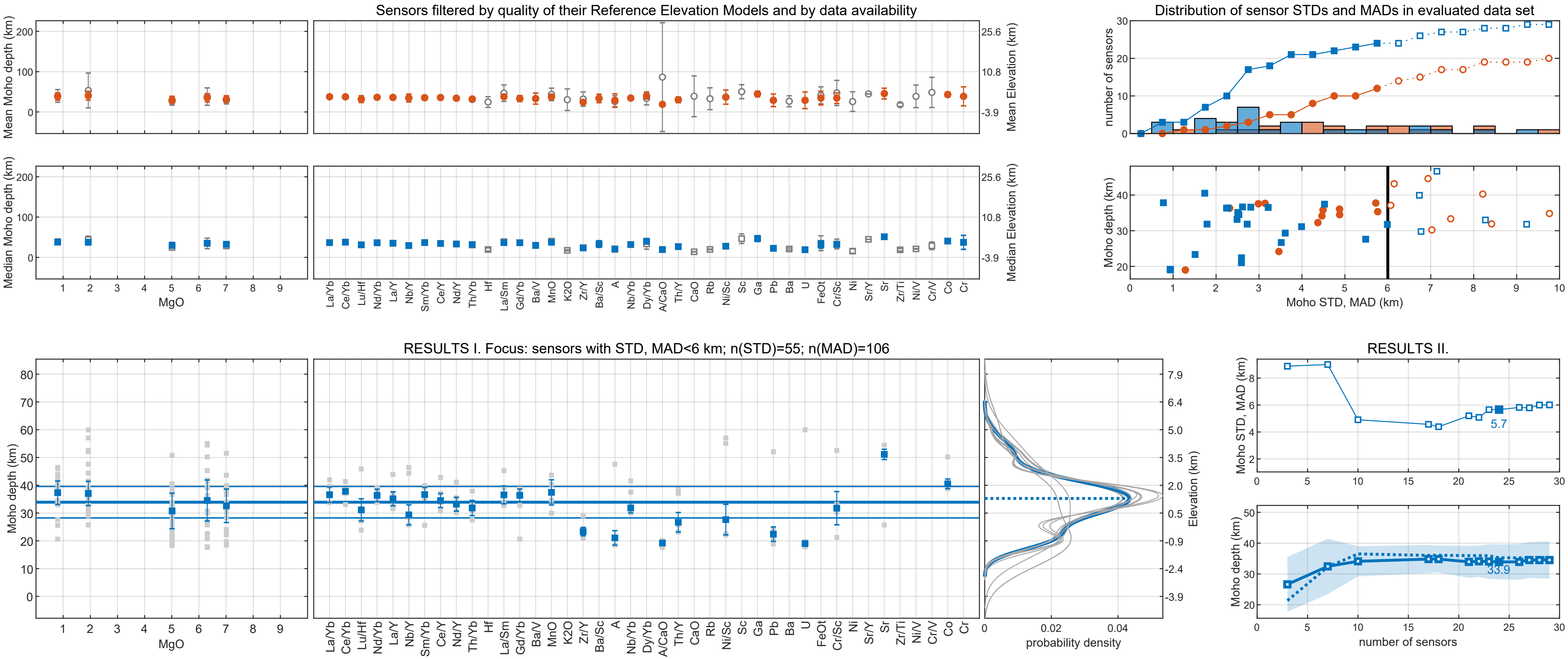

Figure S5-24 Zhangguangcai Range (ZGR) and Jiamusi Region (JR): 196-184 Ma Magmatism

Individual Elevation vs. Age (210-140 Ma) and (196-184 Ma)

TAS diagram (196-184 Ma)

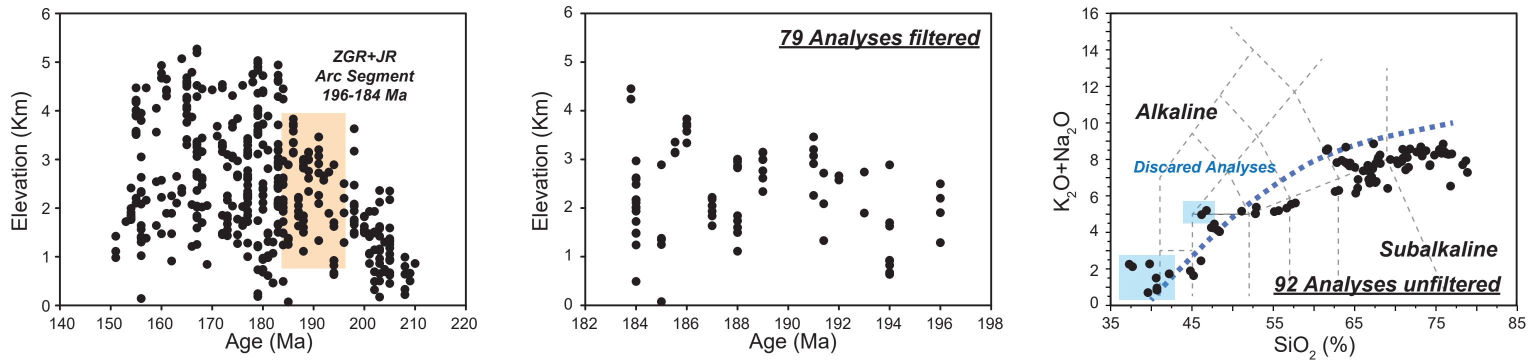

Individual Elevation vs. Latitude (196-184 Ma)

TAS diagram (196-184 Ma)

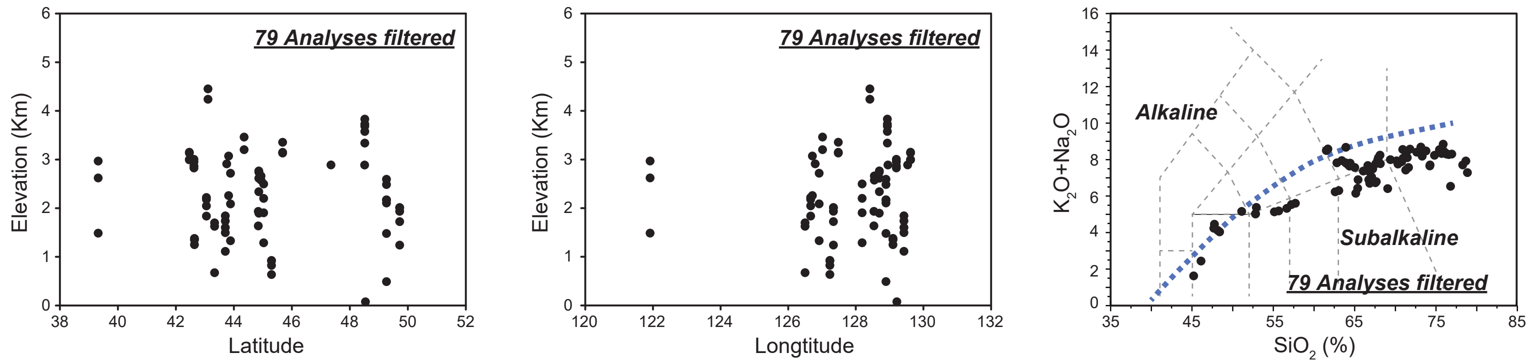

Harker Diagrams (196-184 Ma)

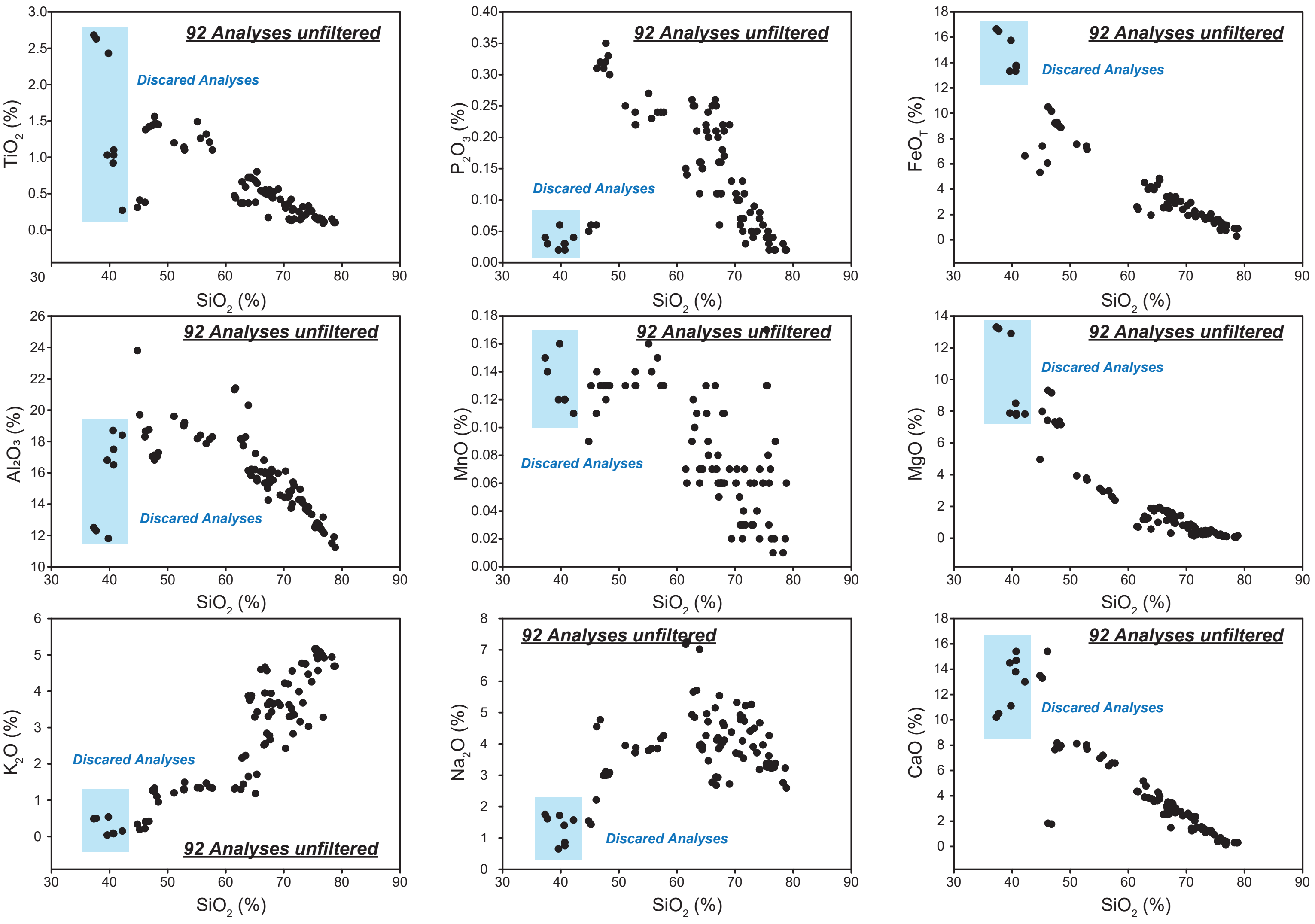

Zhangguangcai Range and Jiamusi Region 196-184 Ma Arc Segment GAME Results

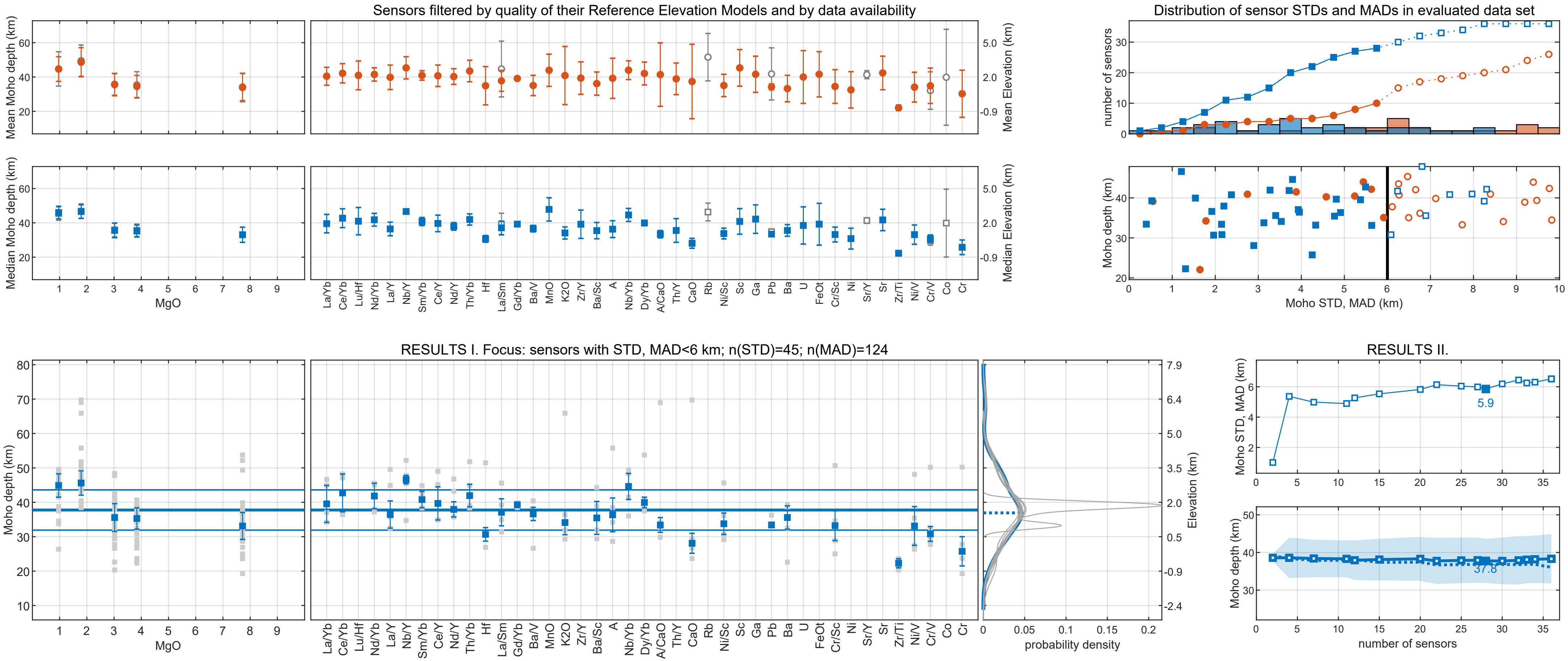

Figure S5-25 Zhangguangcai Range (ZGR) and Jiamusi Region (JR): 183-173 Ma Magmatism

Individual Elevation vs. Age (210-140 Ma) and (183-173 Ma)

TAS diagram (183-173 Ma)

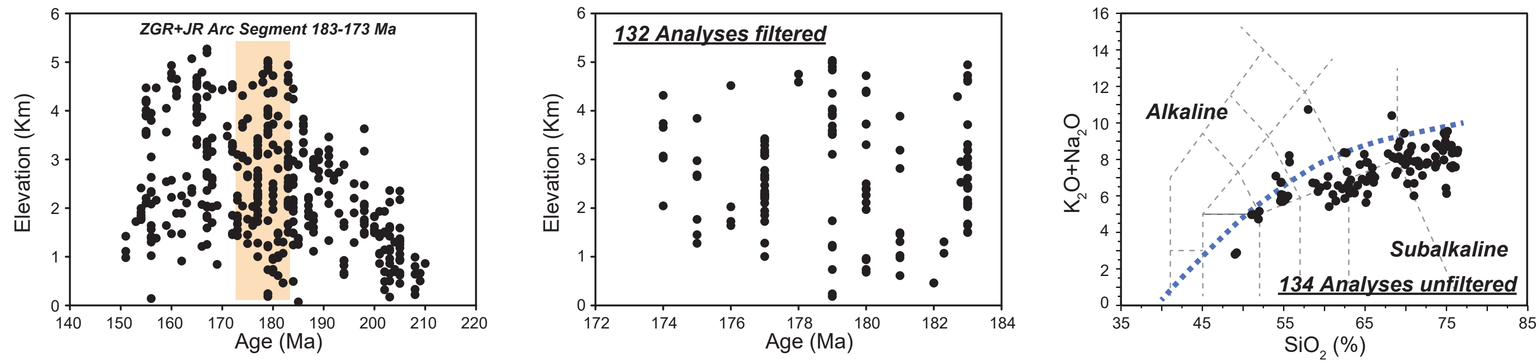

Individual Elevation vs. Latitude (183-173 Ma)

TAS diagram (183-173 Ma)

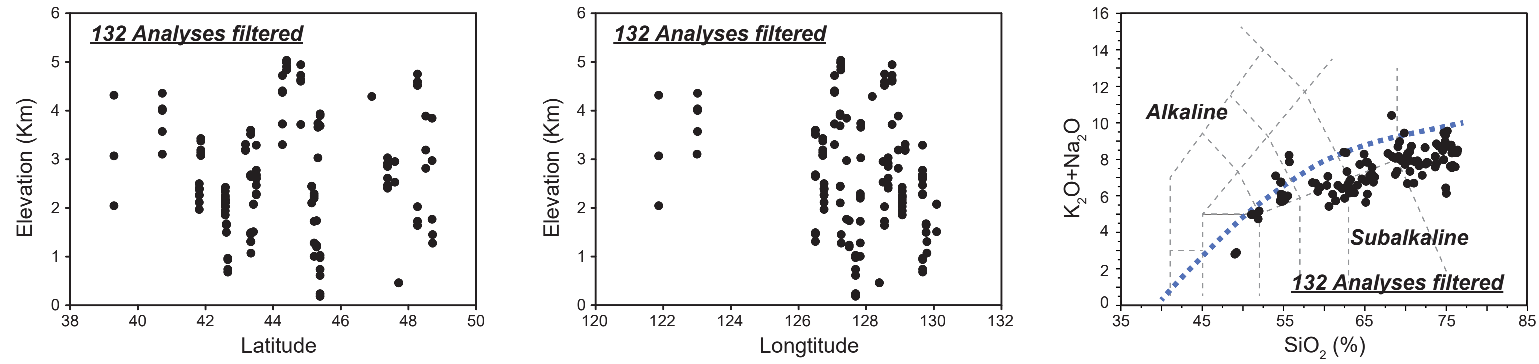

Harker Diagrams (183-173 Ma)

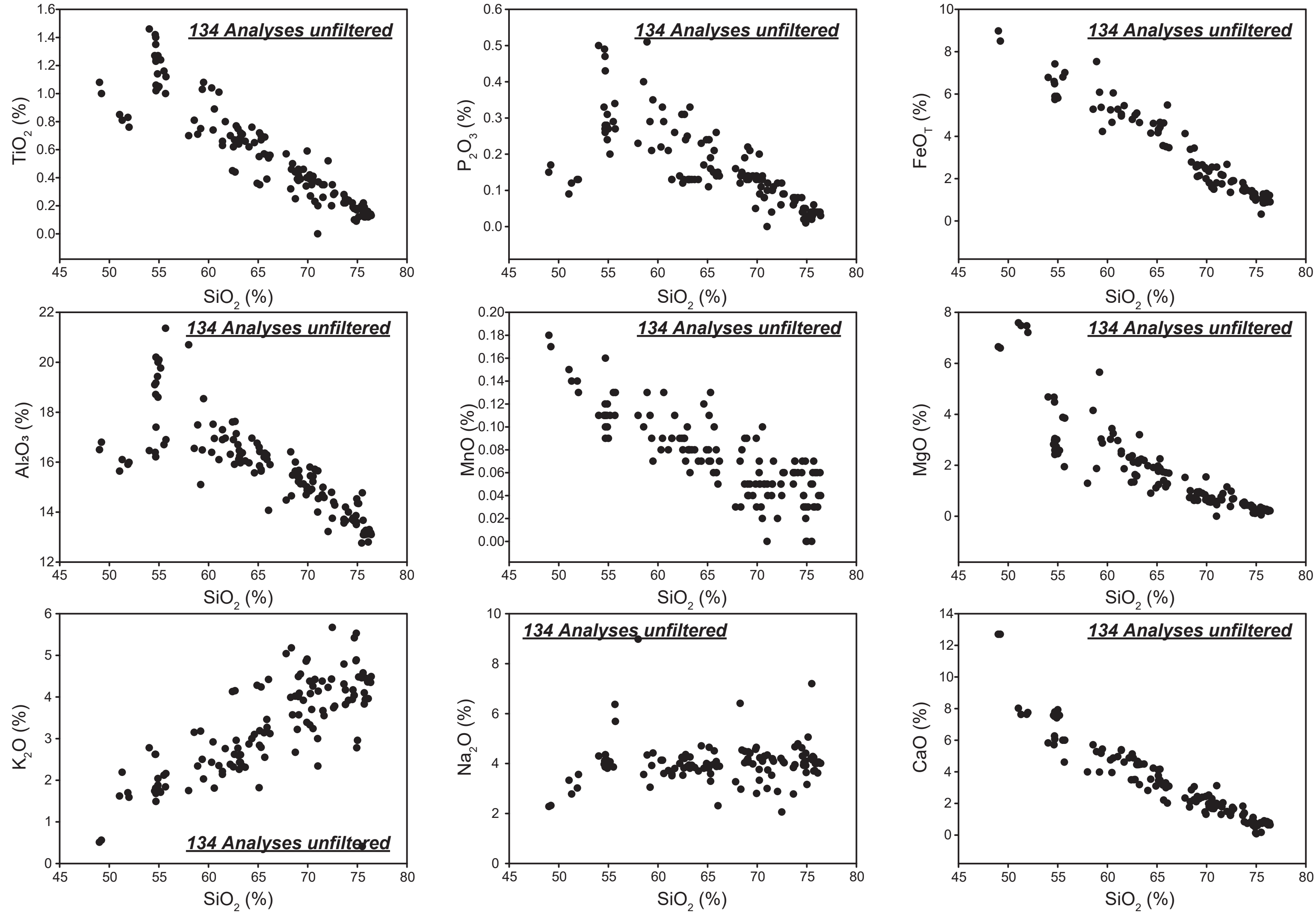

Zhangguangcai Range and Jiamusi Region 183-173 Ma Arc Segment GAME Results

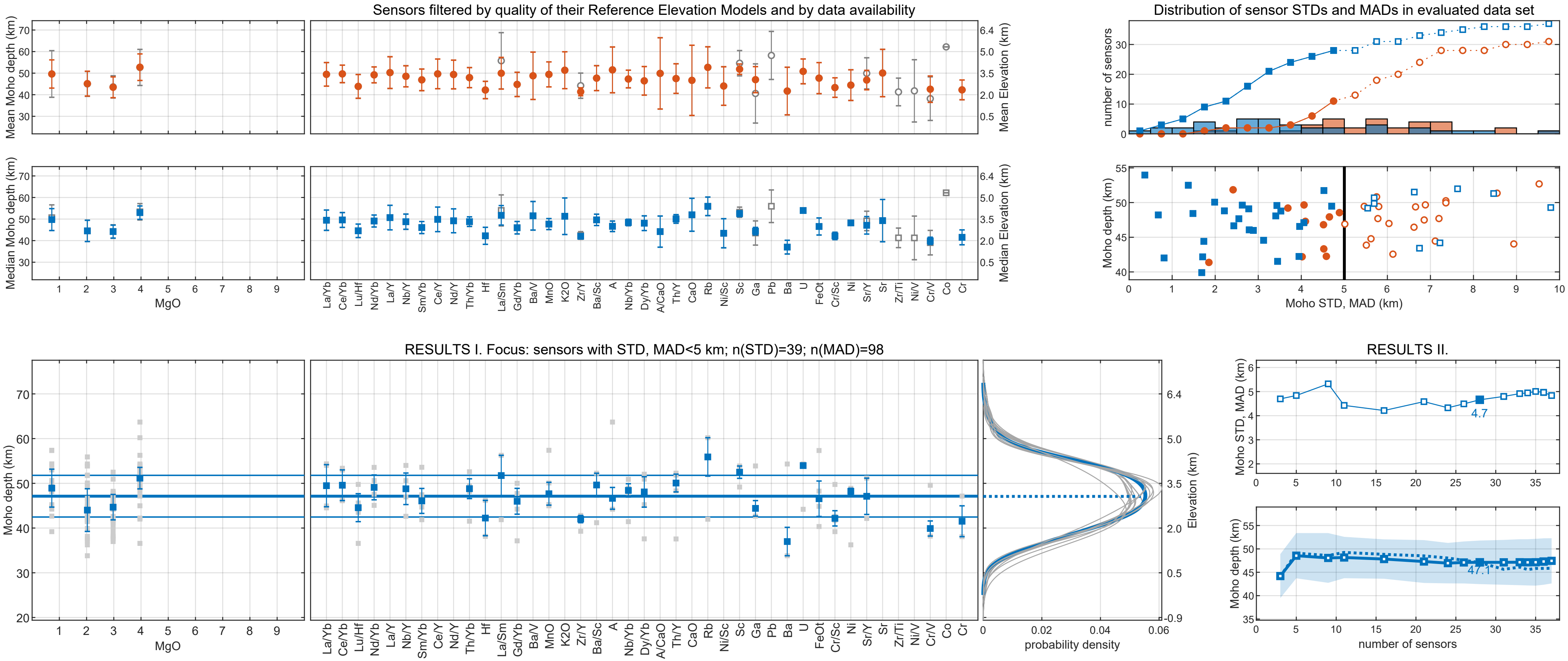

Figure S5-26 Zhangguangcai Range (ZGR) and Jiamusi Region (JR): 113-100 Ma Magmatism

Individual Elevation vs. Age (140-100 Ma)

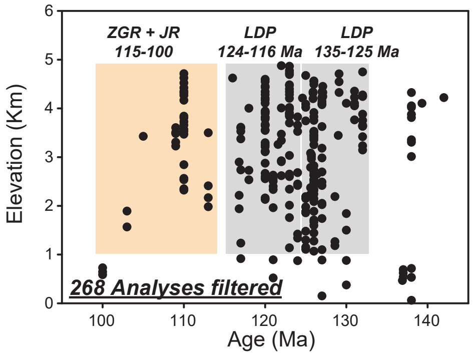

TAS diagram (113-100 Ma)

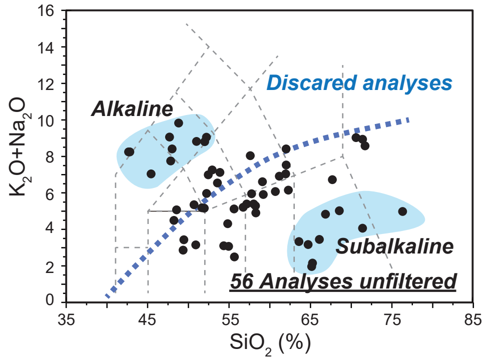

Individual Elevation vs. Latitude and Longtitude (140-100 Ma)

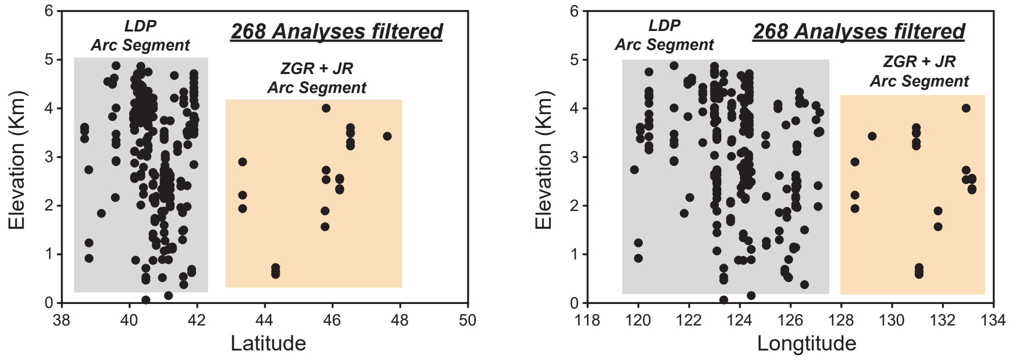

TAS diagram (113-100 Ma)

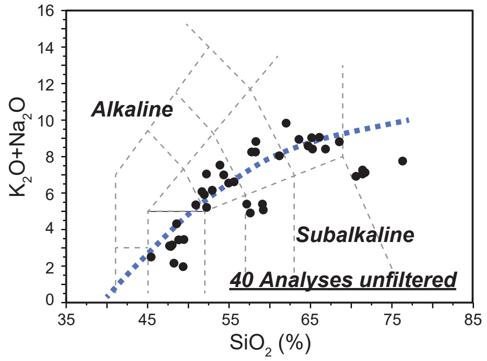

Harker Diagrams (113-100 Ma)

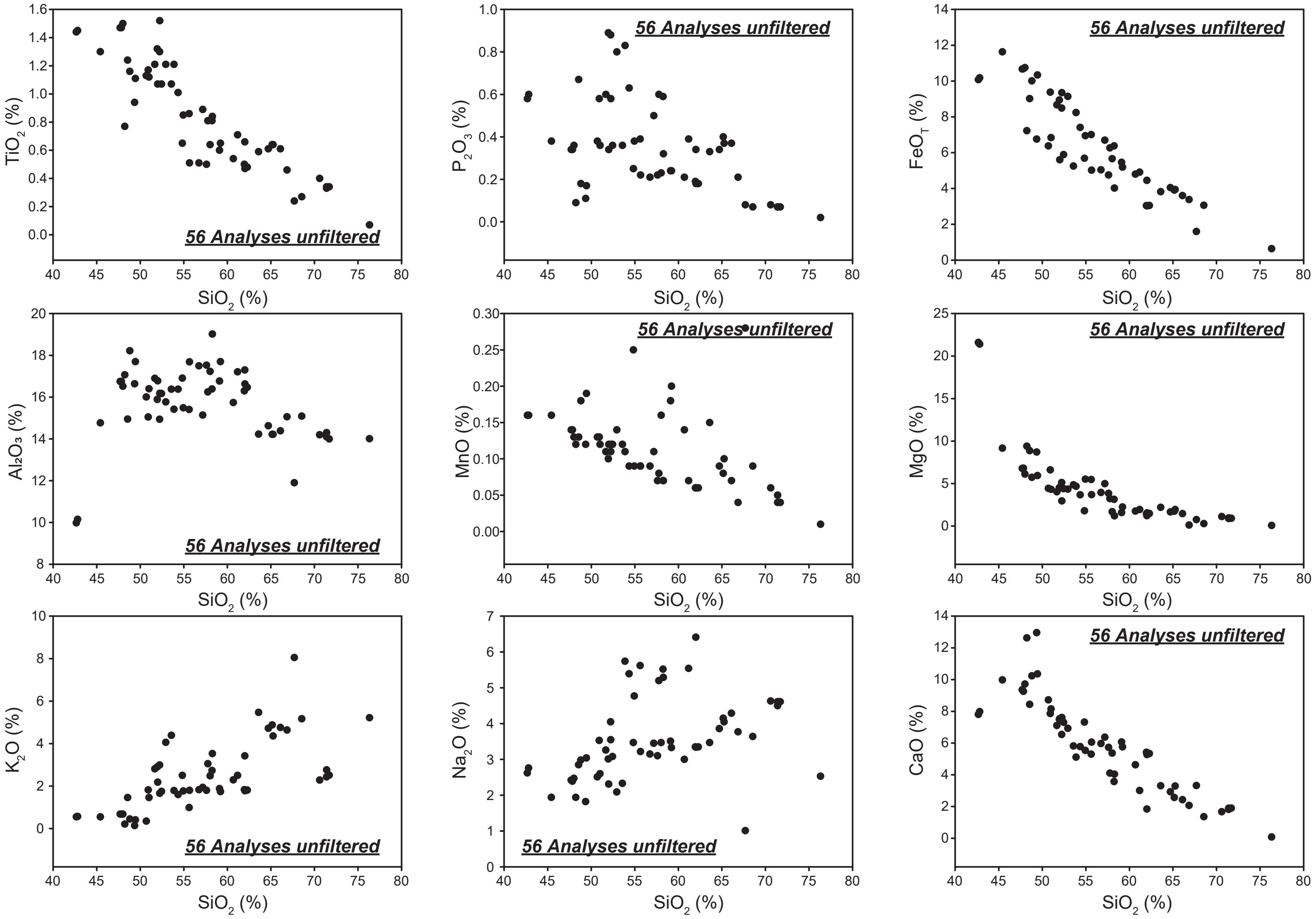

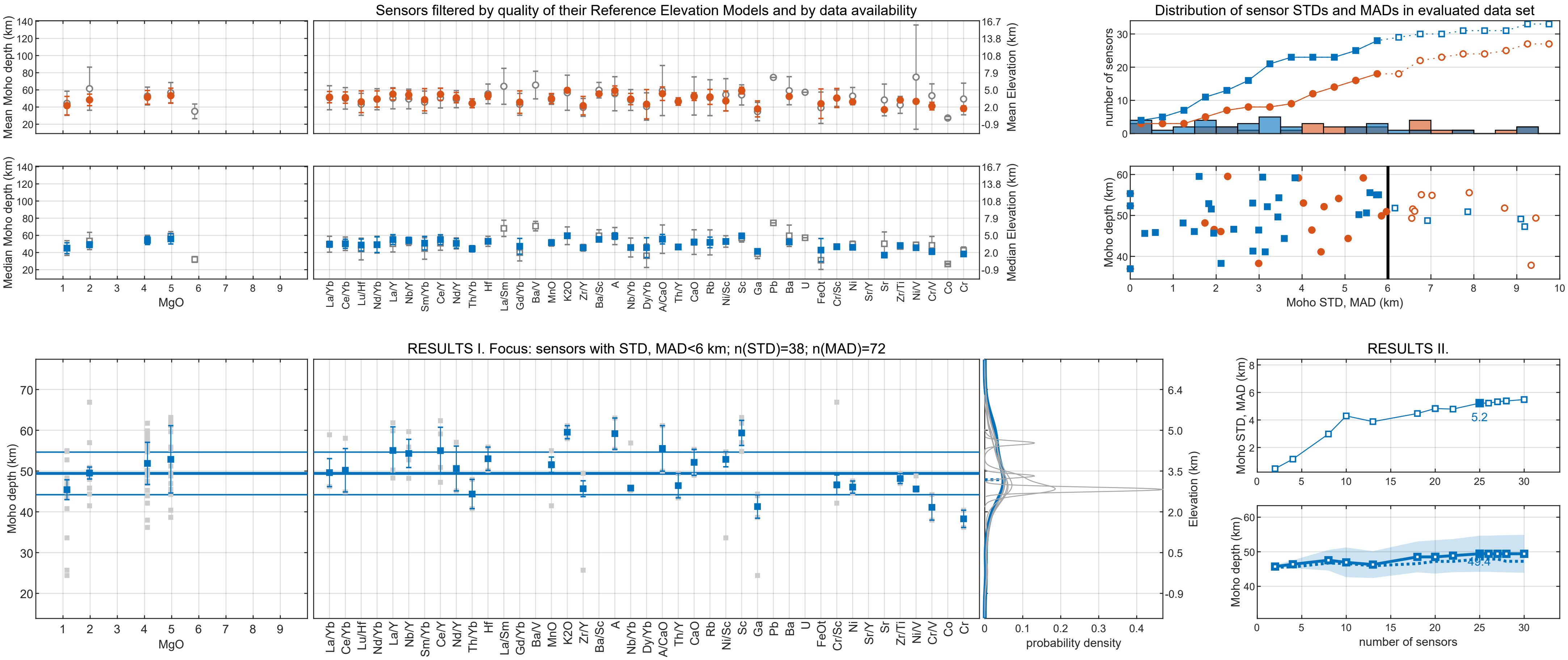

Sensors filtered by quality of their Reference Elevation Models and by data availability
Mean Moho depth (km)
Mean Elevation (km)
Median Moho depth (km)
Median Elevation (km)
MgO
La/Yb Ce/Yb Lu/Hf Nd/Yb La/Y Nb/Y Sm/Yb Ce/Y Nd/Y Th/Yb Hf La/Sm Gd/Yb Ba/V MnO K2O Zr/Y Ba/Sc A Nb/Yb Dy/Yb A/CaO Th/Y CaO Rb Ni/Sc Sc Ga Pb Ba U FeOt Cr/Sc Ni Sr/Y Sr Zr/Ti Ni/V Cr/V Co Cr
Distribution of sensor STDs and MADs in evaluated data set
number of sensors
Moho depth (km)
Moho STD, MAD (km)
RESULTS I. Focus: sensors with STD, MAD<6 km; n(STD)=38; n(MAD)=72
Moho depth (km)
Elevation (km)
probability density
RESULTS II.
Moho STD, MAD (km)
5.2
Moho depth (km)
49.4
number of sensors

Fig. S5. GAME results for each arc segment

Individual elevation versus crystallization age, individual elevation versus latitude, individual elevation versus longtitude, TAS and Harker diagrams, and GAME results for each arc segment. Individual elevations represent the median elevation estimates obtained for the quality-filtered individual samples, computed by using all applicable mohometers that satisfy the imposed εmax limitation.

Yanliao Biota Evolution and Migration

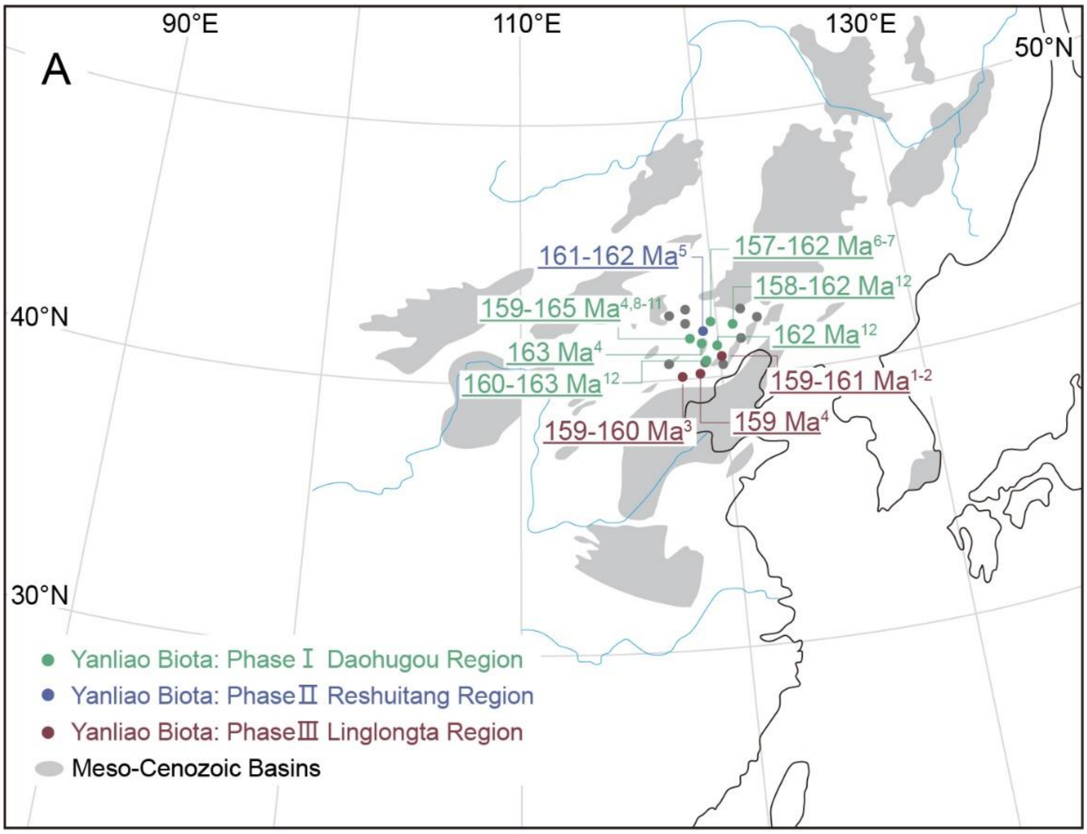

Jehol Biota Evolution and Migration

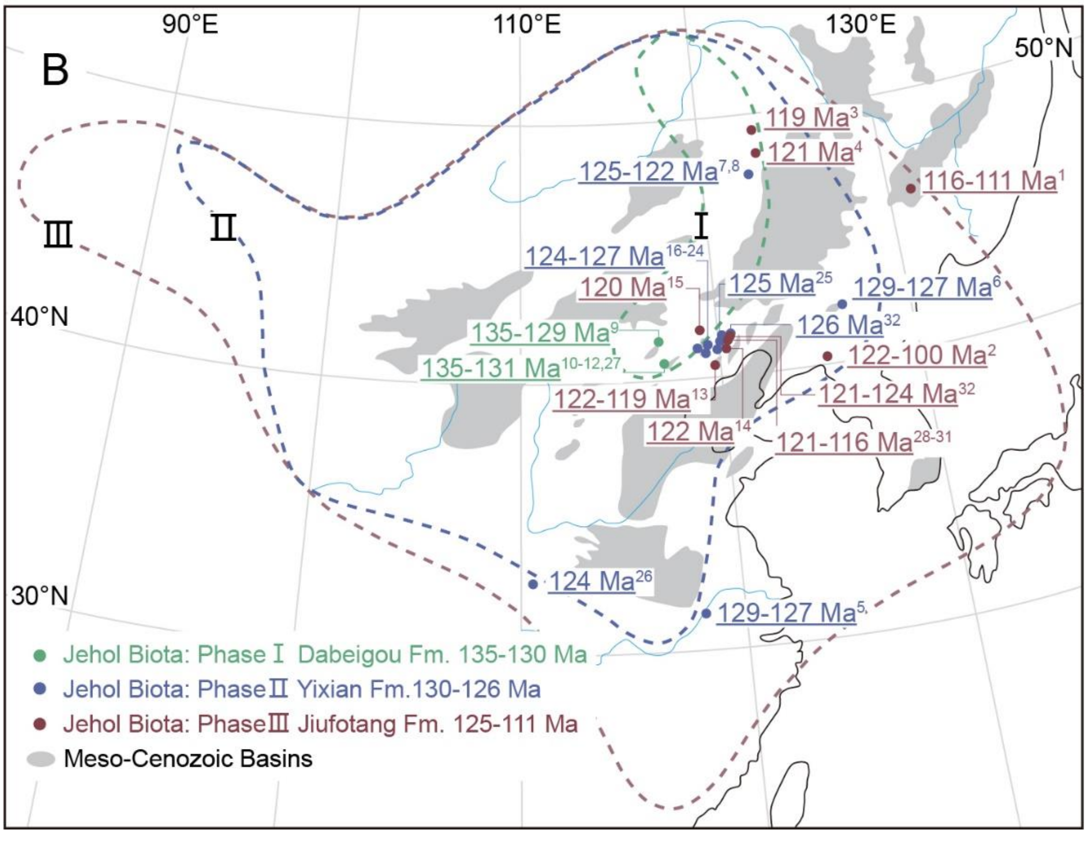

Fig. S6. Evolution and migration trends of the Yanliao and Jehol biotas

Yanliao biota data: 1 and 2-Chu et al. (1) and Wang et al. (2), 3-Yu et al. (3), 4-Yu et al. (4), 5-Li et al. (5), 6-Liu et al. (6), 7-Liu et al. (7), 8-He et al. (8), 9-Meng (9), 10-Chen et al. (10), 11-Liu et al. (11), 12-Zou et al. (12).

Jehol biota data: 1-Chen et al. (13), 2-Chang et al. (14), 3-Yu et al. (15), 4-Wang et al. (16), 5-Chang et al. (17), 6-Wang and Zhong (18), 7-Li and Reisz (19), 8-Li et al. (20), 9-Yang et al. (21), 10-Fang et al. (22), 11-Liu et al. (23), 12-Zhang et al. (24), 13-Yu et al. (25), 14-Chang et al. (26), 15-He et al. (27), 16-Chang et al. (28), 17-He et al. (29), 18-Li et al. (30), 19-Swisher et al. (31), 20-Swisher et al. (32), 21-Wang et al. (33), 22-Yang et al. (34), 23-Zhong et al. (35), 24-Zhu et al. (36), 25-MacLennan et al. (37), 26-Song et al. (38), 27-Shen et al. (39), 28-Sun et al. (40), 29-Ying et al. (41), 30-Zhang et al. (42), 31-Li et al. (43), 32-Zhong et al. (44). The distributions of the Jehol biota at different evolution stages are modified from Zhou et al. (45). The related references see in the References 02 for the Table S6.

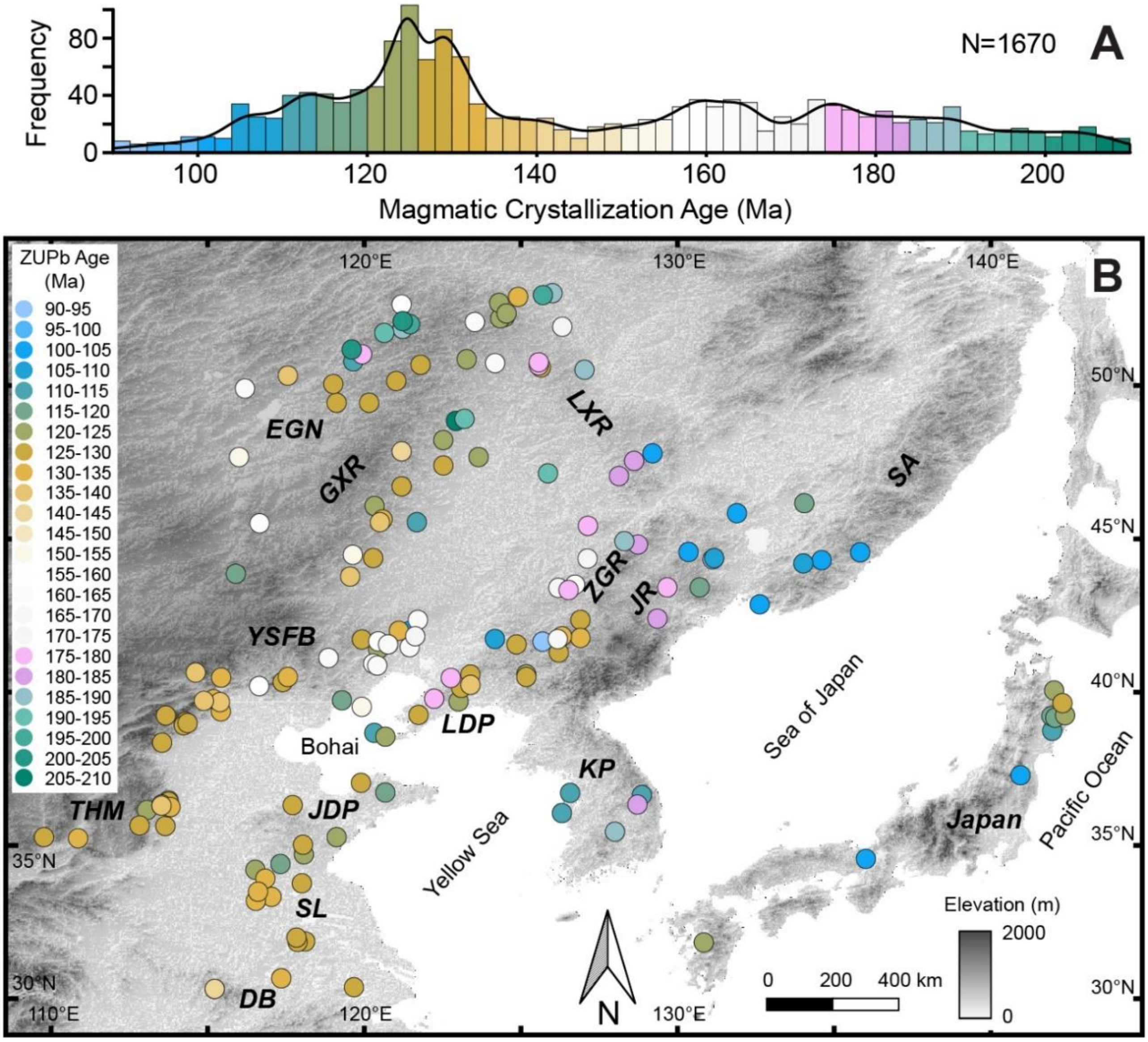

Fig. S7. Distribution of Sr/Y and $(La/Yb)_{CN}$ ratio data subsets

A: The kernel density estimate (KDE) of individual age data. Details for each data subset or individual data can be found in the Supplementary Material. B: Topographic map of the northeastern Asian, with color-coded dots representing Sr/Y and $(La/Yb)_{CN}$ ratio data subsets. These data subsets group individual analyses with similar magmatic crystallization ages and geographic locations. The abbreviations are as follows: EGN–Erguna Region; GXR–Great Xing'an Range; LXR–Lesser Xing'an Range; ZGR–Zhangguangcai Range; JR–Jiamusi Region; SA–Sikhote-Alin; YSFB–Yanshan Fold Belt; LDP–Liaodong Peninsula; JDP–Jiaodong Peninsula; SL–Sulu Region; DB–Dabie Mountain; THM–Taihangshan.

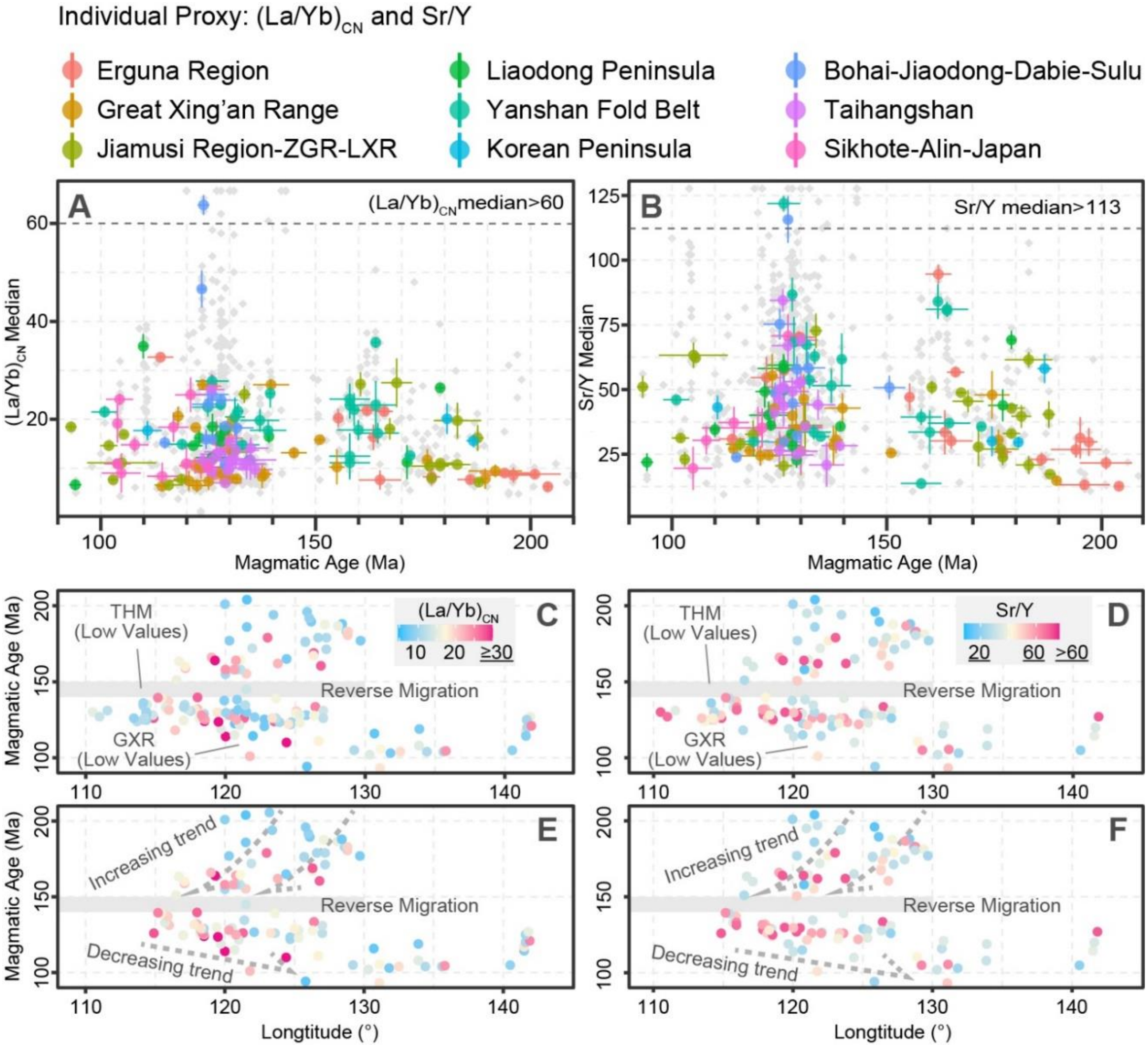

Fig. S8. Sr/Y and (La/Yb)CN ratios vs. magmatic crystallization ages

A-B: Distribution of Sr/Y and (La/Yb)CN ratios versus magmatic crystallization ages, filtering according to the principles described in Section 2. C-F: Magmatic crystallization ages plotted against longitudes, with color codes indicating Sr/Y and (La/Yb)CN ratios. In Figures 8E and 8F, the datasets with low Sr/Y and (La/Yb)CN ratios from the Great Xing'an Range and Taihangshan are excluded.

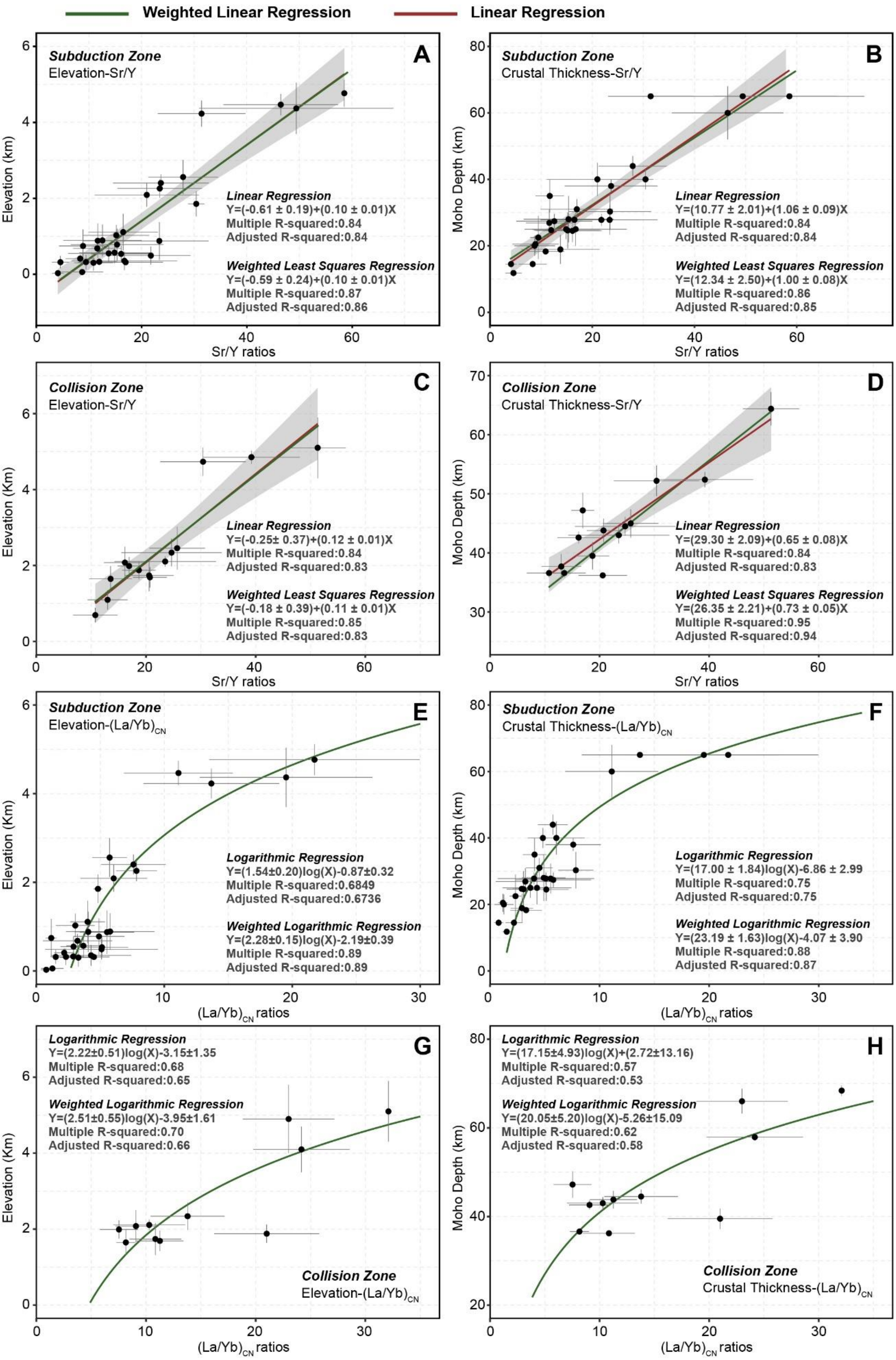

Weighted Linear Regression
Linear Regression
A
Subduction Zone
Elevation-Sr/Y
Linear Regression
Y=(-0.61 ± 0.19)+(0.10 ± 0.01)X
Multiple R-squared:0.84
Adjusted R-squared:0.84
Weighted Least Squares Regression
Y=(-0.59 ± 0.24)+(0.10 ± 0.01)X
Multiple R-squared:0.87
Adjusted R-squared:0.86
Elevation (km)
Sr/Y ratios
B
Subduction Zone
Crustal Thickness-Sr/Y
Linear Regression
Y=(10.77 ± 2.01)+(1.06 ± 0.09)X
Multiple R-squared:0.84
Adjusted R-squared:0.84
Weighted Least Squares Regression
Y=(12.34 ± 2.50)+(1.00 ± 0.08)X
Multiple R-squared:0.86
Adjusted R-squared:0.85
Moho Depth (km)
Sr/Y ratios
C
Collision Zone
Elevation-Sr/Y
Linear Regression
Y=(-0.25± 0.37)+(0.12 ± 0.01)X
Multiple R-squared:0.84
Adjusted R-squared:0.83
Weighted Least Squares Regression
Y=(-0.18 ± 0.39)+(0.11 ± 0.01)X
Multiple R-squared:0.85
Adjusted R-squared:0.83
Elevation (Km)
Sr/Y ratios
D
Collision Zone
Crustal Thickness-Sr/Y
Linear Regression
Y=(29.30 ± 2.09)+(0.65 ± 0.08)X
Multiple R-squared:0.84
Adjusted R-squared:0.83
Weighted Least Squares Regression
Y=(26.35 ± 2.21)+(0.73 ± 0.05)X
Multiple R-squared:0.95
Adjusted R-squared:0.94
Moho Depth (km)
Sr/Y ratios
E
Subduction Zone
Elevation-(La/Yb)CN
Logarithmic Regression
Y=(1.54±0.20)log(X)-0.87±0.32
Multiple R-squared:0.6849
Adjusted R-squared:0.6736
Weighted Logarithmic Regression
Y=(2.28±0.15)log(X)-2.19±0.39
Multiple R-squared:0.89
Adjusted R-squared:0.89
Elevation (Km)
(La/Yb)CN ratios
F
Sbuduction Zone
Crustal Thickness-(La/Yb)CN
Logarithmic Regression
Y=(17.00 ± 1.84)log(X)-6.86 ± 2.99
Multiple R-squared:0.75
Adjusted R-squared:0.75
Weighted Logarithmic Regression
Y=(23.19 ± 1.63)log(X)-4.07 ± 3.90
Multiple R-squared:0.88
Adjusted R-squared:0.87
Moho Depth (km)
(La/Yb)CN ratios
G
Logarithmic Regression
Y=(2.22±0.51)log(X)-3.15±1.35
Multiple R-squared:0.68
Adjusted R-squared:0.65
Weighted Logarithmic Regression
Y=(2.51±0.55)log(X)-3.95±1.61
Multiple R-squared:0.70
Adjusted R-squared:0.66
Collision Zone
Elevation-(La/Yb)CN
Elevation (Km)
(La/Yb)CN ratios
H
Logarithmic Regression
Y=(17.15±4.93)log(X)+(2.72±13.16)
Multiple R-squared:0.57
Adjusted R-squared:0.53
Weighted Logarithmic Regression
Y=(20.05±5.20)log(X)-5.26±15.09
Multiple R-squared:0.62
Adjusted R-squared:0.58
Collision Zone
Crustal Thickness-(La/Yb)CN
Moho Depth (km)
(La/Yb)CN ratios

Fig. S9. Global empirical correlations

Global empirical correlations between average elevations and Moho depths, and median Sr/Y and $(La/Yb)_{CN}$ ratios from subduction zones (A, B, E, F) and collision zones (C, D, G, H). The geochemical data, along with their corresponding Moho depths and elevations are referenced from Chapman et al. [35], Hu et al. [36], and Profeta et al. [37]. The median Sr/Y and $(La/Yb)_{CN}$ ratio data for magmatic rocks in the subduction zones are during the Pliocene-present, while those for collision zones span from the Middle–Late Miocene to the present. We performed least squares regression and weighted least squares regression through these data subsets to derive the best direct correlation equations between geochemical compositions, elevations, and crustal thicknesses. This allows us to predict the paleo-elevations and paleo-crustal thicknesses based on the Sr/Y and $(La/Yb)_{CN}$ proxies, which is achieved in R programming language.

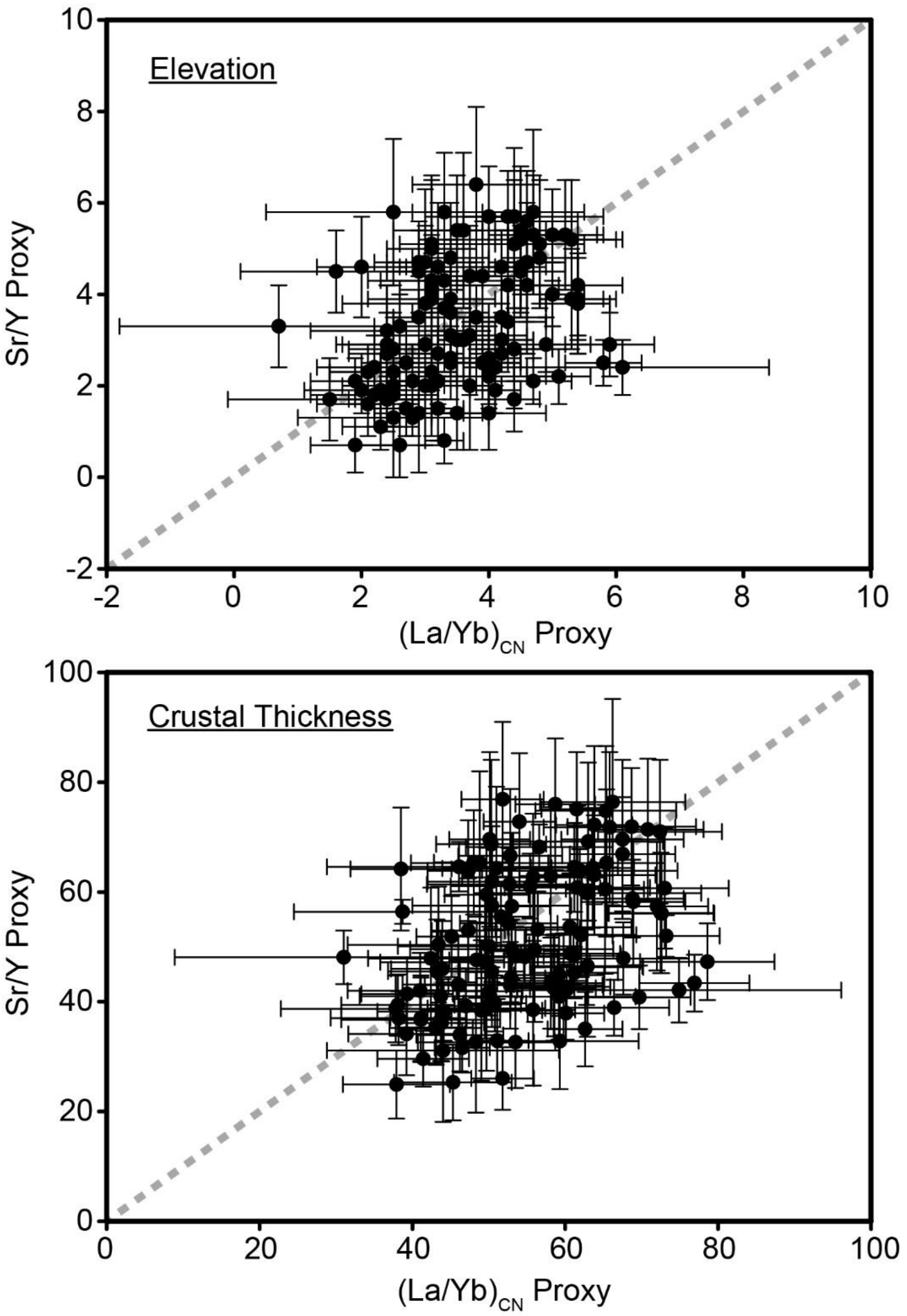

Fig. S10. Comparison of predicted paleo-elevations and Moho depths

The comparison of predicted paleo-elevations and Moho depths for Mesozoic northeastern Asia, calculated using Sr/Y and $(La/Yb)_{CN}$ proxies based on the Equations (1-8). "CN" denotes the values normalized to chondritic values of McDonough and Sun [39].

Supplementary Tables

Table S1. Zircon U-Pb datasets in Northeastern Asia

Zircon U-Pb ages published for Northeastern Asia. References and data mainly refer to Wu et al. [5], Ma and Xu [12], and Zhang et al. [25]. Some papers do not provide accurate GPS locations; therefore, we have estimated the GPS coordinates based on the geological maps included in those papers.

Table S2. Whole-rock major and trace element datasets in Northeastern Asia

Whole-rock major and trace element data (approximately 4500 samples) from Northeastern Asia, processed by GAME. Data sources are provided in Supplementary Table S2. Some papers do not publish accurate GPS locations; therefore, we have estimated the GPS locations based on the geological maps provided in those papers. Similarly, for samples lacking precise ages, we have estimated the ages according to published zircon U-Pb age data from surrounding plutons. The estimated GPS locations and zircon U-Pb ages are highlighted in red.

Table S3. Detailed GAME parameters

Detailed GAME parameters used for the different arc segments.

Table S4. Calculation results of paleo-temperatures

Calculation results of paleo-temperatures across the Jurassic–Early Cretaceous Northeastern Asia.

Table S5. Summary of tectonic, sedimentation, and exhumation events

Summary of major tectonic deformations, sedimentations, and rapid cooling and exhumation events across the Jurassic–Early Cretaceous Northeastern Asia. References are provided below.

Table S6. Summary of the timing of the Yanliao and Jehol Biotas

Summary of the timing of the Yanliao and Jehol Biotas in Northeastern Asia. References are provided below.

Table S7. Evidences of climate and elevation for the Jehol and Yanliao Biotas

Evidences of climate and elevation for the Jehol and Yanliao Biotas in Northeastern Asia. References are provided below.

Table S8. Evidences of climate and biodiversity evolution

Evidences of climate and biodiversity evolution in Late Triassic–Early Cretaceous Northeastern Asia. References are provided below.

Table S9. Sr/Y and La/Yb ratio datasets

Sr/Y and La/Yb ratio datasets, along with predicted paleo-elevations and Moho depths in Jurassic–Early Cretaceous Northeastern Asia.

REFERENCES CITED

1. Liu Y, Li W and Feng Z *et al*. A review of the Paleozoic tectonics in the eastern part of Central Asian Orogenic Belt. *Gondwana Res* 2017; **43**: 123–148.

2. Xiao W, Windley B and Sun S *et al*. A tale of amalgamation of three Permo-Triassic collage systems in Central Asia: Oroclines, sutures, and terminal accretion. *Annu Rev Earth Planet Sci* 2015; **43**: 16.1 to 16.31.

3. Wang T, Tong Y and Xiao W *et al*. Rollback, scissor-like closure of the Mongol-Okhotsk Ocean and formation of an orocline: Magmatic migration based on a large archive of age data. *Natl Sci Rev* 2022; **9**: nwab210.

4. Zhu CY, Zhao G and Sun M *et al*. Subduction between the Jiamusi and Songliao blocks: geochronological and geochemical constraints from granitoids within the Zhangguangcailing orogen, northeastern China. *Lithosphere* 2017; **9**: 515–533.

5. Wu F, Sun D and Ge W *et al*. Geochronology of the Phanerozoic granitoids in northeastern China. *J Asian Earth Sci* 2011; **41**: 1–30.

6. Lin C, Liu S and Shi X *et al*. Late Jurassic–Early Cretaceous deformation in the western Yanshan fold-thrust belt: Insights from syntectonic sedimentation in the Chicheng Basin, North China. *Tectonics* 2019; **38**: 2449–2476.

7. Ma Q, Zhong Y and Yin Q *et al*. High-resolution chronostratigraphy of late Mesozoic sequences in northern North China: Implications for the linkages among intracontinental orogeny, volcanism, Jehol Biota, and Pacific plate subduction. *Geology* 2023; **52**: 45–50.

8. Zhang F, Dilek Y and Chen H *et al*. Structural architecture and stratigraphic record of Late Mesozoic sedimentary basins in NE China: Tectonic archives of the Late Cretaceous continental margin evolution in East Asia. *Earth-Sci Rev* 2017; **171**: 598–620.

9. Lin W and Wei W. Late Mesozoic extensional tectonics in the North China Craton and its adjacent regions: A review and synthesis. *Int Geol Rev* 2020; **62**: 811–839.

10. Xu W, Pei F and Wang F *et al*. Spatial-temporal relationships of Mesozoic volcanic rocks in NE China: constraints on tectonic overprinting and transformations between multiple tectonic regimes. *J Asian Earth Sci* 2013; **74**: 167–193.

11. Wu F, Yang J and Xu Y *et al*. Destruction of the North China Craton in the Mesozoic. *Annu Rev Earth Planet Sci* 2019; **47**: 173–95.

12. Ma Q and Xu YG. Magmatic perspective on subduction of Paleo-Pacific plate and initiation of big mantle wedge in East Asia. *Earth-Sci Rev* 2021; **213**: 103473.

13. Zhao P, Xu B and Chen Y. Evolution and final closure of the Mongol-Okhotsk Ocean. *Sci China Earth Sci* 2023; **66**: 2497–2513.

14. Zhang Y, Qiu E and Dong S *et al*. Late Mesozoic intracontinental deformation and magmatism in North and NE China in response to multi-plate convergence in NE Asia: An overview and new view. *Tectonophysics* 2022; **835**: 229377.

15. Luffi P and Ducea MN. Chemical Mohometry: Assessing crustal thickness of ancient orogens using geochemical and isotopic data. *Rev Geophys* 2022; **60**: e2021RG000753.

16. Irvine TN and Baragar WRA. A guide to the chemical classification of the common volcanic rocks. *Can J Earth Sci* 1971; **8**: 523–548.

17. Guo J, Huang XL and Zhang L *et al*. Decoupling between SrPb and NdHf isotopes of Mesozoic mafic rocks in the eastern North China Craton: Implication for multi-stage modification of sub-continental lithospheric mantle. *Lithos* 2023; 442–443: 107096.

18. Koua KAD, Sun H and Li J *et al*. Petrogenesis of Early Cretaceous granitoids and mafic enclaves from the Jiaodong Peninsula, eastern China: Implications for crust-mantle interaction, tectonic evolution and gold mineralization. *J Asian Earth Sci* 2022; **228**: 105096.

19. Wu H, Liu SA and He Y *et al*. Mesozoic slab-derived magmas from mid-eastern China: Responses to a ridge-transform fault-ridge subduction system. *Chem Geol* 2023; **617**: 121259.

20. Cheong ACS, Jung MJ and Jo HJ *et al*. Lithospheric thinning and ignition of a Cordilleran magmatic flare-up: Geochemical and O-Hf isotopic constraints from Cretaceous plutons in southern Korea. *Geosci Front* 2023; **14**: 101492.

21. Choi SH and Kim JI. Emplacement ages and petrogenesis of the Sunchang and Namwon granitoids, South Korea. *Lithos* 2023; **444–445**: 107107.

22. Cheong ACS and Jo HJ. Tectonomagmatic evolution of a Jurassic Cordilleran flare-up along the Korean Peninsula: Geochronological and geochemical constraints from granitoid rocks. *Gondwana Res* 2020; **88**: 21–44.

23. Kawaguchi K, Oh CW and Jeong JW. Geochemistry, zircon UPb ages and LuHf isotopes of Triassic plutons in the eastern Gyeonggi Massif, Korean Peninsula: Magma genesis and geodynamic implications for East Asia. *Lithos* 2023; **436–437**: 106955.

24. Kim SW, Kwon S and Ko K *et al*. Geochronological and geochemical implications of Early to Middle Jurassic continental adakitic arc magmatism in the Korean Peninsula. *Lithos* 2015; **227**: 225–240.

25. Zhang S, Zhao Y and Davis GA *et al*. Temporal and spatial variations of Mesozoic magmatism and deformation in the North China Craton: Implications for lithospheric thinning and decratonization. *Earth-Sci Rev* 2014; **131**: 49–87.

26. Wang Z, Liu L and Fu Y *et al*. Multistage plate subduction controls intraplate volcanism and cratonic lithospheric thinning in Northeast Asia. *Earth-Sci Rev* 2023; **246**: 104590.

27. Huang H, Wang T and Guo L *et al*. Crustal modification influenced by multiple convergent systems: Insights from Mesozoic magmatism in northeastern China. *Earth-Sci Rev* 2024; **252**: 104737.

28. Zhao P, Appel E and Deng C *et al*. Bending of the western Mongolian blocks initiated the Late Triassic closure of the Mongol-Okhotsk Ocean and formation of the Tuva-Mongol Orocline. *Tectonics* 2023; **42**: e2022TC007475.

29. Wang P, Mattern F and Didenko NA *et al*. Tectonics and cycle system of the Cretaceous Songliao Basin: An inverted active continental margin basin. *Earth-Sci Rev* 2016; **159**: 82–102.

30. Zhang F, Chen H and Yu X *et al*. Early Cretaceous volcanism in the northern Songliao Basin, NE China, and its geodynamic implication. *Gondwana Res* 2011; **19**: 163–176.

31. Liu C, Nicotra E and Shan X *et al*. The Cretaceous volcanism of the Songliao Basin: Mantle sources, magma evolution processes and implications for the NE China geodynamics - A review. *Earth-Sci Rev* 2023; **237**: 104294.

32. Ji Z, Meng QA and Wan CB *et al*. Early Cretaceous adakitic lavas and A-type rhyolites in the Songliao Basin, NE China: Implications for the mechanism of lithospheric extension. *Gondwana Res* 2019; **71**: 28–48.

33. Zhu CY, Zhao G and Ji J *et al*. Subduction between the Jiamusi and Songliao blocks: Geological, geochronological and geochemical constraints from the Heilongjiang Complex. *Lithos* 2017; **282–283**: 128–144.

34. Long XY, Xu WL and Guo P *et al*. Opening and closure history of the Mudanjiang Ocean in the eastern Central Asian Orogenic Belt: Geochronological and geochemical constraints from early Mesozoic intrusive rocks. *Gondwana Res* 2020; **84**: 111–130.

35. Chapman JB, Ducea MN and DeCelles PG *et al*. Tracking changes in crustal thickness during orogenic evolution with Sr/Y: An example from the North American Cordillera. *Geology* 2015; **43**: 919–922.

36. Hu F, Wu F and Chapman JB *et al*. Quantitatively tracking the elevation of the Tibetan Plateau since the Cretaceous: Insights from whole-rock Sr/Y and La/Yb ratios. *Geophys Res Lett* 2020; **47**: e2020GL089202.

37. Profeta L, Ducea MN and Chapman JB *et al*. Quantifying crustal thickness over time in magmatic arcs. *Sci Rep* 2015; **5**: 1–7.

38. Hu F, Ducea MN and Liu S *et al*. Quantifying crustal thickness in continental collisional belts: Global perspective and a geologic application. *Sci Rep* 2017; **7**: 7058.

39. McDonough WF and Sun SS. The composition of the Earth. *Chem Geol* 1995; **120**: 223–253.

40. Sundell KE, Laskowski AK and Kapp P *et al*. Jurassic to Neogene quantitative crustal thickness estimates in southern Tibet. *GSA today* 2021; **31**: 4–10.

41. Tang M, Ji WQ and Chu X *et al*. Reconstructing crustal thickness evolution from europium anomalies in detrital zircons. *Geology* 2020; **49**(1): 76–80.
